# Δ^4^-dn-*iso*-OPDA, a bioactive plant hormone of *Marchantia polymorpha*

**DOI:** 10.1016/j.isci.2024.110191

**Published:** 2024-06-06

**Authors:** Takuya Kaji, Yuho Nishizato, Hidenori Yoshimatsu, Akiyoshi Yoda, Wenting Liang, Andrea Chini, Gemma Fernández-Barbero, Kei Nozawa, Junko Kyozuka, Roberto Solano, Minoru Ueda

**Affiliations:** 1 Graduate School of Science, Tohoku University, 6-3, Aramaki-Aza-Aoba, Aoba-ku, Sendai 980-8578, Japan; 2 Graduate School of Life Sciences, Tohoku University, 2-1-1, Katahira, Aoba-ku, Sendai 980-8577, Japan; 3 Plant Molecular Genetics Department, National Centre for Biotechnology (CNB), Consejo Superior de Investigaciones Cientificas (CSIC), Campus University Autonoma, 28049 Madrid, Spain; 4 Department of Molecular and Chemical Life Sciences, Graduate School of Life Sciences, Tohoku University, Sendai 980-8577, Japan

**Keywords:** Biochemistry, Plant biochemistry, Plant biology

## Abstract

Significant progress has been recently made in our understanding of the evolution of jasmonates biosynthesis and signaling. The bioactive jasmonate activating COI1-JAZ co-receptor differs in bryophytes and vascular plants. Dinor*-iso*-12-oxo-phytodienoic acid (dn-*iso*-OPDA) is the bioactive hormone in bryophytes and lycophytes. However, further studies showed that the full activation of hormone signaling in *Marchantia polymorpha* requires additional unidentified hormones. Δ^4^-dn-OPDAs were previously identified as novel bioactive jasmonates in *M. polymorpha*. In this paper, we describe the major bioactive isomer of Δ^4^-dn-OPDAs as Δ^4^-dn-*iso*-OPDA through chemical synthesis, receptor binding assay, and biological activity in *M. polymorpha*. In addition, we disclosed that Δ^4^-dn-*cis*-OPDA is a biosynthetic precursor of Δ^4^-dn-*iso*-OPDA. We demonstrated that in planta *cis*-to-*iso* conversion of Δ^4^-dn-*cis*-OPDA occurs in the biosynthesis of Δ^4^-dn-*iso*-OPDA, defining a key biosynthetic step in the chemical evolution of hormone structure. We predict that these findings will facilitate further understanding of the molecular evolution of plant hormone signaling.

## Highlights

• Δ^4^-dn-*iso*-OPDA is identified as the major bioactive isomer of Δ^4^-dn-OPDAs
• Δ^4^-dn-*iso*-OPDA is biosynthesized through isomerization of Δ^4^-dn-*cis*-OPDA
• Δ^4^-dn-OPDAs are distributed in bryophyte lineages

## Graphical abstract

**Figure:**
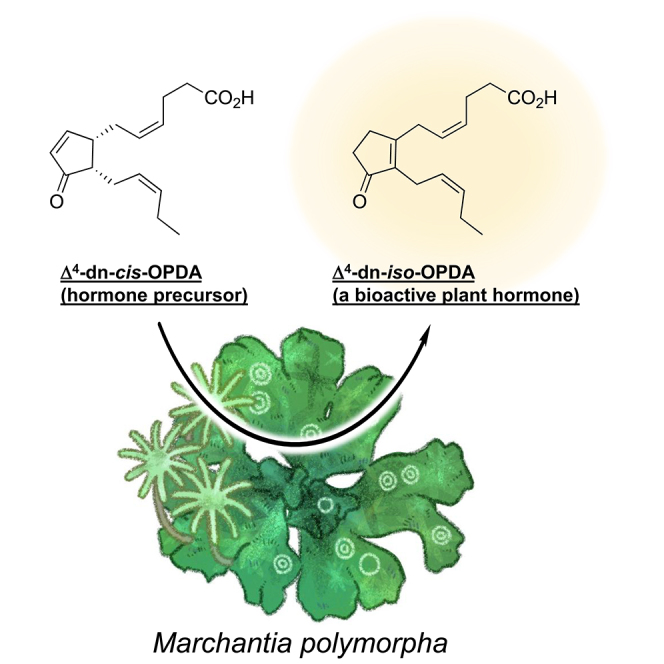

## Introduction

**Figure 1:**
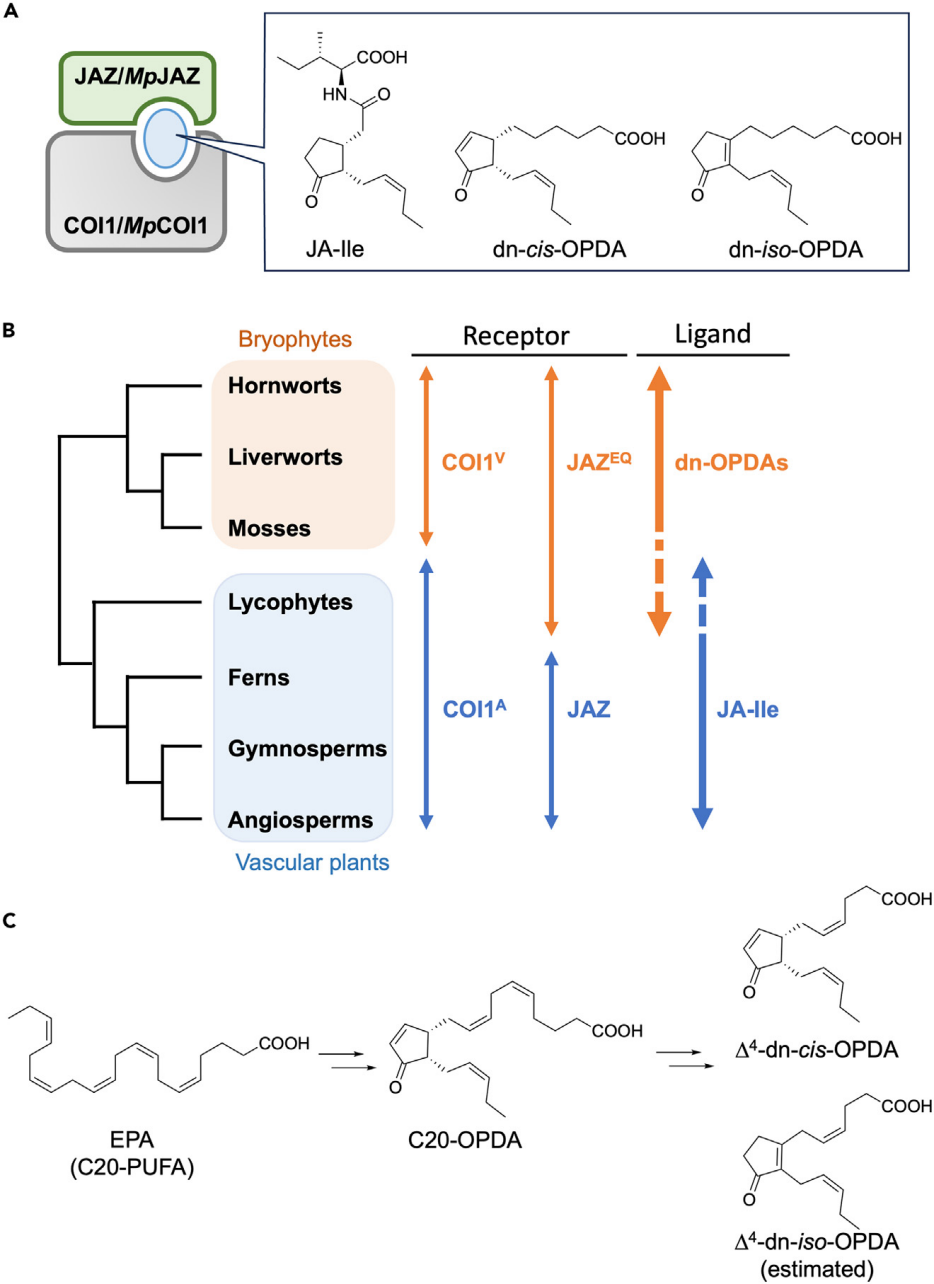
Structure, molecular evolution, and biosynthesis of jasmonates (A) Structures of JA-Ile and jasmonates dn-*cis*/*iso*-OPDA and the COI1-JAZ/MpCOI1-MpJAZ co-receptor. (B) Phylogenetic tree of land plants and coevolution of COI1/JAZ co-receptor components First, the evolution from COI1^V^ to COI1^A^, in which amino acid residue in ligand binding pocket changed from V to A, and then from JAZ^EQ^ to JAZ, in which a part of ligand interacting motif changed, occurred to induce a stepwise change of the ligand from dn-OPDAs to JA-Ile.^26^^,^^27^ Blue and orange arrows indicate the presence of conserved sequences (COI1 and JAZ) or the ligand (JA-Ile or dn-OPDAs) in the corresponding plant lineages. Dashed lines in JA-Ile or dn-OPDAs rectangles indicate that the precise usage and ligand perception within lycophytes has not been elucidated yet. (C) Structures of Δ^4^-dn-*cis*/*iso*-OPDA and their biosynthetic pathway. Δ^4^-dn-OPDAs are biosynthesized from EPA, a C20-PUFA, through β-oxidation of C20-OPDA intermediate.

Recently, significant progress has been made in our understanding of the molecular evolution of plant hormone signaling.^1^^,^^2^^,^^3^^,^^4^^,^^5^^,^^6^^,^^7^ The changes in the plant hormone system from aquatic to terrestrial life are particularly interesting. The transition of plants to terrestrial life required the acquisition of defense traits against new enemies, such as pathogens and herbivorous insects that did not exist in aquatic environment.^8^^,^^9^^,^^10^^,^^11^^,^^12^ The analyses of the model bryophyte *Marchantia polymorpha* were instrumental to study the conservation plant hormone signaling modules, including defense hormones against pathogens and herbivores.^13^^,^^14^^,^^15^^,^^16^^,^^17^^,^^18^^,^^19^ Identifying the defense hormone, an oxylipin called jasmonate,^20^ of bryophytes is essential as the first step in understanding the molecular evolution of land plant hormone signaling pathways. (+)-7-*iso*-Jasmonoyl-L-isoleucine (JA-Ile, Figure 1A) is the primary jasmonate of vascular plants.^21^^,^^22^ In angiosperms such as *Arabidopsis thaliana*, JA-Ile is biosynthesized in response to wounding or attack by enemies and is perceived by the COI1-JAZ co-receptor, which is composed of the CORONATINE INSENSITIVE 1 (COI1), a component of E3 ubiquitin ligase, and the JASMONATE ZIM domain (JAZ) transcriptional repressor protein (Figure 1A).^10^^,^^23^^,^^24^^,^^25^

Recently, Monte et al. identified the ancestor of JA-Ile, dinor-*cis*/*iso*-12-oxo-phytodienoic acid (dn-*cis*/*iso*-OPDA, Figure 1A) in the model bryophyte, *M. polymorpha*, and revealed that ligand-receptor co-evolution occurred during the evolution of land plants (Figure 1B).^14^^,^^26^^,^^27^ JA-Ile and dn-*cis*/*iso*-OPDA are biosynthesized from C18- or C16- polyunsaturated fatty acid (PUFA) upon wounding (Figure S1).^20^^,^^28^ However, the biosynthesis of dn-*cis*/*iso*-OPDAs depends on MpFAD5, an enzyme required for the biosynthesis of PUFAs in the hexadecanoid pathway (Figure S1).^29^ The accumulation of dn-*cis*/*iso*-OPDA in wounded *M. polymorpha* was almost abolished in the *Mpfad5-3* mutant line, yet the activation of the jasmonate signaling pathway still occurred, suggesting the existence of additional redundant hormones in *M. polymorpha*.^30^ Thus, we previously identified the Δ^4^-dn-OPDAs (Figure 1C) as novel bioactive jasmonate hormones of *M. polymorpha,* which are biosynthesized by wounding from eicosapentaenoic acid (EPA), a long-chain PUFA of C20, independent of Mp*FAD5* (Figure 1C).^31^ Both dn-OPDAs and Δ^4^-dn-OPDAs are required for full activation of the jasmonate signaling pathway in *M. polymorpha*. Thus, Δ^4^-dn-OPDAs may play important roles in the molecular evolution of plant jasmonate signaling pathway. Ultra-performance liquid chromatography-tandem mass spectrometry (UPLC-MS/MS) analyses revealed that two possible isomers corresponding to Δ^4^-dn-OPDAs were biosynthesized in *M. polymorpha* (Figure 1C).^31^ However, although our previous results suggested that Δ^4^-dn-*iso*-OPDA is the major active isomer of Δ^4^-dn-OPDAs, the impossibility to obtain this isomer by chemical synthesis impaired a formal demonstration. Here we developed a new method for chemical synthesis of Δ^4^-dn-*iso*-OPDA and demonstrated that it is the relevant bioactive form of Δ^4^-dn-OPDA.

## Results

### Chemical synthesis and identification of Δ^4^-dn-*iso*-OPDA

**Figure 2:**
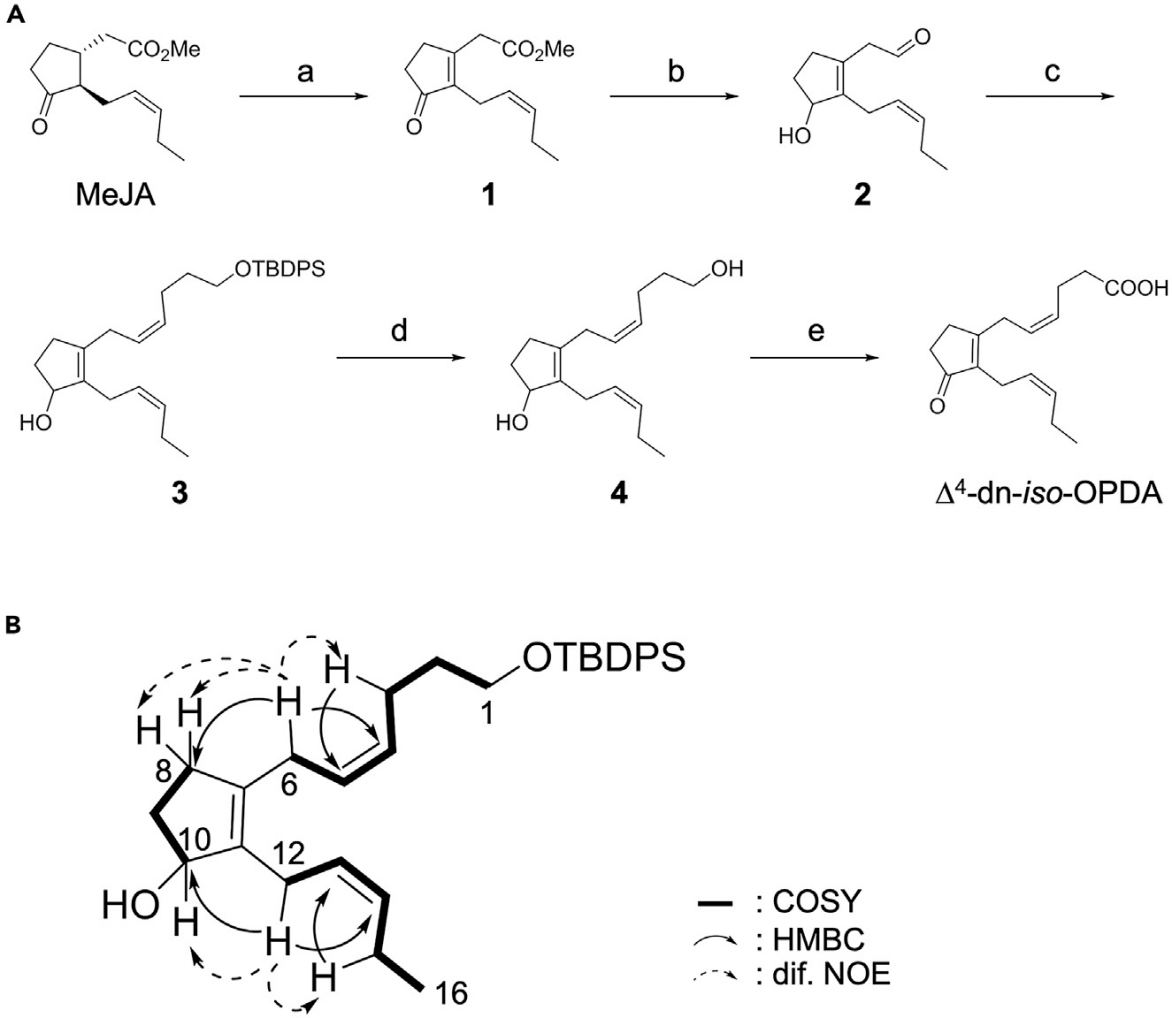
Chemical synthesis of Δ^4^-dn-*iso*-OPDA (A) Synthetic route of Δ^4^-dn-*iso*-OPDA and observed byproducts: (a) CuBr_2_, cyclohexene, Me-propionate, CHCl_3_, 80°C, 34%; (b) DIBAL, toluene, CH_2_Cl_2_, –78°C, 70%; (c) TBDPSO(CH_2_)_4_PPh_3_I, NaHMDS, THF, −78°C, 37%; (d) TBAF, THF, 85%; (e) AZADOL, PhI(OAc)_2_, CH_2_Cl_2_-pH 7 buffer, 69%. (B) Structure, selected COSY and HMBC correlations, and differential NOE in compound **3**.

The minor isomer of Δ^4^-dn-OPDAs was identified as Δ^4^-dn-*cis*-OPDA using a synthetic standard (Figure 1C).^31^ On the other hand, the major isomer of the same *m/z* value had a different retention time on UPLC-MS/MS analysis and was predicted as Δ^4^-dn-*iso*-OPDA in the previous study.^31^ Thus, we synthesized Δ^4^-dn-*iso*-OPDA to confirm that it is the major isomer identified *in vivo* (Figure 1C). The structural feature of Δ^4^-dn-*iso*-OPDA is that it has a cyclopentenone ring with two alkyl chains containing a *Z*-olefin, and the *Z*-olefin in one side chain constitutes a skipped diene with the olefin of the cyclopentenone ring.^32^^,^^33^^,^^34^ According to our previous synthesis of dn-*cis*-OPDA,^35^ we introduced a ketone in the final step of the synthesis of Δ^4^-dn-*iso*-OPDA (Figure 2A). Methyl 3,7-didehydrojasmonate (3,7-ddh-MeJA, 1) was synthesized through a previously reported single-step conversion of (±)-methyl jasmonate (MeJA) by halogenation/elimination using CuBr_2_ (34%).^36^ The reduction of the methyl ester to an aldehyde, and the ketone to a secondary alcohol by diisobutylaluminium hydride (DIBAL) afforded the aldehyde 2 in a moderate isolated yield (70%). A Wittig reaction using 2 and *tert*-butyldiphenylchlorosilanoxyl (TBDPSO)(CH_2_)_4_PPh_3_I afforded the compound 3 in 37%. The structure of 3 was confirmed from H-H correlations by correlation spectroscopy (COSY), C–H correlations by heteronuclear single quantum correlation (HSQC), long-range C–H correlations by heteronuclear multiple bond coherence (HMBC), and stereochemical correlations by differential (dif.) nuclear overhauser effect (NOE) measurements (Figures 2B and S2A–S2O): ^1^H-NMR spectrum showed that there are four olefinic protons (Figure S2A). Structures of C1-C6, C8-C10, and C12-C16 spin systems were established by COSY and HSQC analyses (Figures S2B–S2J), and the four olefinic protons were connected to C-4/C-5 and C-13/C-14 olefins (Figures S2C and S2I). ^13^C-NMR indicated the existence of a quaternary olefin (Figure S2A). Each partial structure was connected from HMBC analysis (Figures S2K–S2N) and dif. NOE analysis (Figure S2O): although direct HMBC correlation to C-7/C-11 olefinic carbon was not observed, HMBC correlation of H-6 to C-8 and H-12 to C-10 were observed (Figures 2B and S2N). In addition, the observed NOEs between H-6 and H-8/8′ and H-12 and H-10 established the structure of compound 3 (Figures 2B and S2O). The *Z*-configurations of the two olefins were confirmed by the NOE correlation between the two oppositely located allylic protons (Figures 2B and S2O). Subsequent deprotection of 3 afforded diol 4. Finally, the obtained diol 4 was converted to the desired Δ^4^-dn-*iso*-OPDA in a single step by 2-azaadamantane *N*-oxyl (AZADO) oxidation in a pH 7 buffer at room temperature in 69% yield. The total yield was 15% in 4 steps from 3,7-ddh-MeJA (5.2% from MeJA in 5 steps).

**Figure 3:**
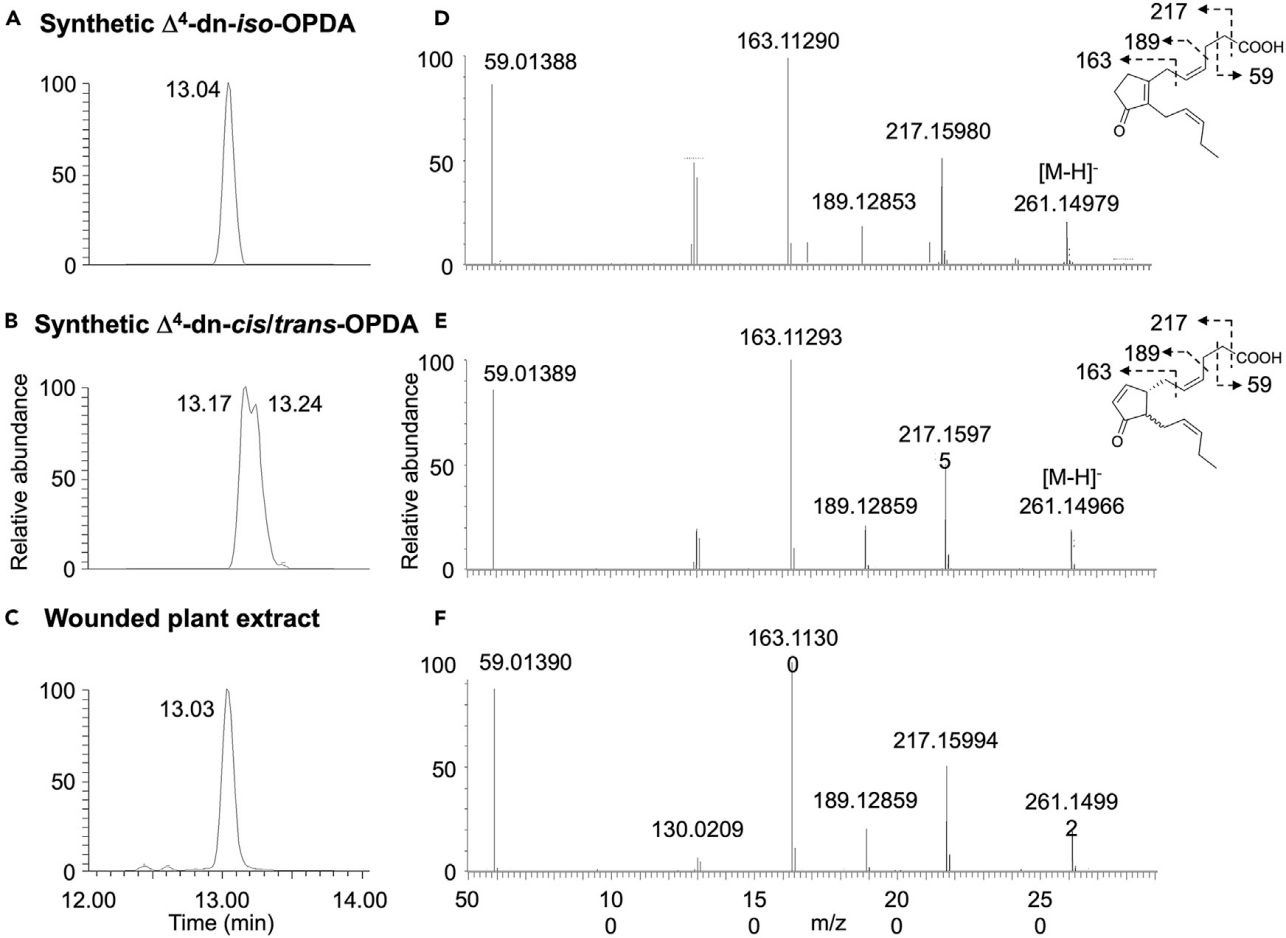
UHPLC-MS/MS analyses of wound-induced accumulation of Δ^4^-dn-OPDAs UHPLC-MS/MS chromatogram of (A) synthetic Δ^4^-dn-*iso*-OPDA in comparison with (B) synthetic Δ^4^-dn-*cis*/*trans*-OPDA and (C) the major Δ^4^-dn-OPDA isomer accumulating in *M. polymorpha* at 2 h after wounding. For detection and quantitative analyses, we used a full MS experiment with MS/MS confirmation in the negative-ion mode. Fragment ion analysis was performed on (D) synthetic Δ^4^-dn-*iso*-OPDA in comparison with (E) synthetic Δ^4^-dn-*cis*/*trans*-OPDA and (F) the major Δ^4^-dn-OPDA isomer accumulating in *M. polymorpha* after wounding.

By UPLC-MS and UPLC-MS/MS analyses, we compared the newly synthetized pure Δ^4^-dn-*iso*-OPDA (Figure 3A) and previously synthesized isomeric mixture Δ^4^-dn-c*is*/*trans*-OPDA (Figure 3B) with major Δ^4^-dn-OPDA isomer accumulated in wounded *M. polymorpha* plants (Figure 3C). The result demonstrated that Δ^4^-dn-*iso*-OPDA, eluted at R_t_ = 13.04 min, was identified as the major isomer accumulated in wounded plants (Figures 3A and 3C). Furthermore, the MS/MS analyses demonstrated that the fragmentation pattern of the synthetic Δ^4^-dn-*iso*-OPDA (Figure 3D) and wound-induced product at R_t_ = 13.04 min are identical (Figure 3F), indicating that they are the same compound.

### Bioactivity of Δ^4^-dinor-*iso*-OPDA on *M. polymorpha*

**Figure 4:**
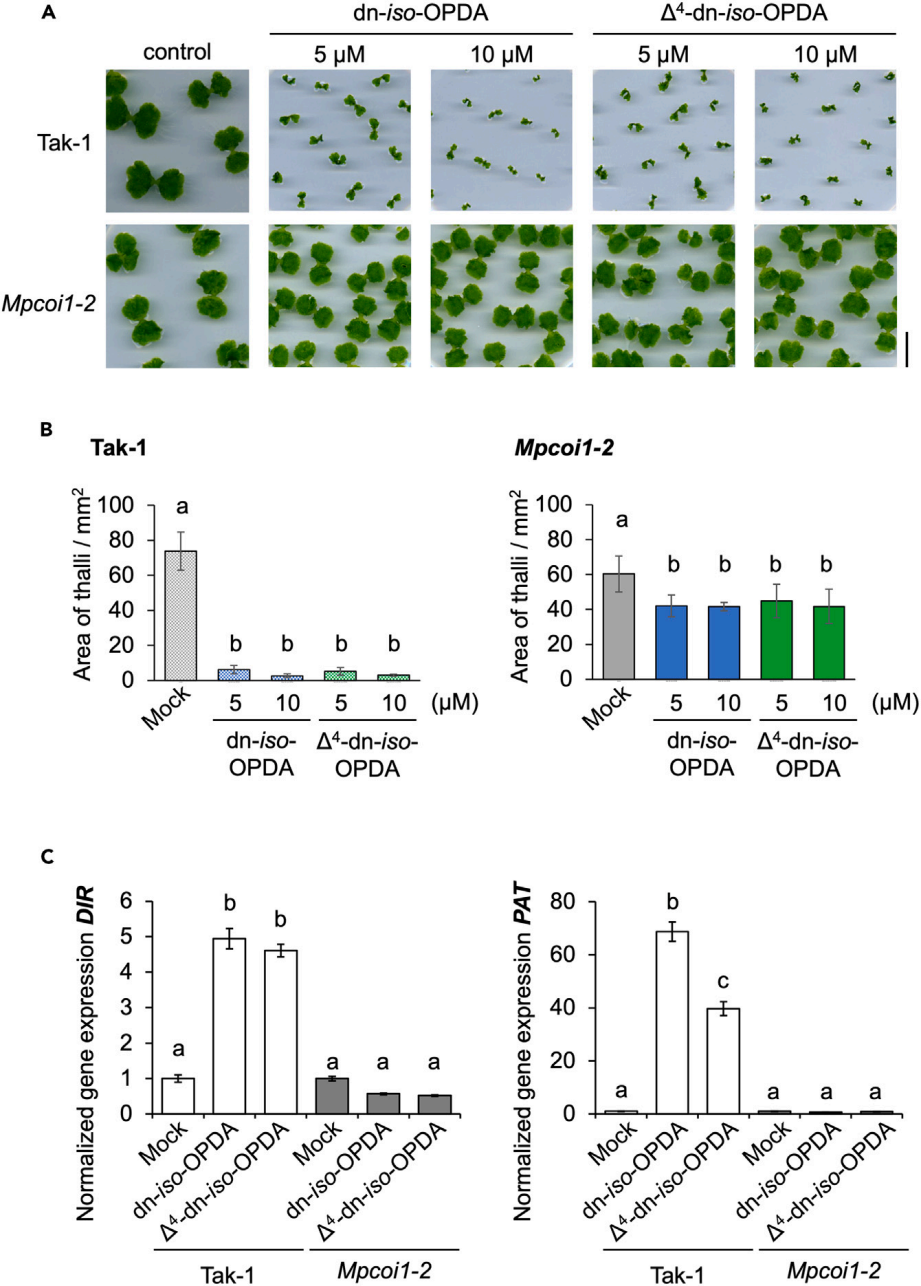
Biological activities of synthetic Δ^4^-dn-*iso*-OPDA (A) Effect of various concentrations (5, 10 μM) of dn-*iso*-OPDA and Δ^4^-dn-*iso*-OPDA on the growth of WT (Tak-1), *Mpcoi1-2* mutant line. Experiments were repeated twice with similar results. Scale bar, 1 cm. (B) Bar graph display of (A). Different letters indicate statistically significant differences between the percentage of growth in mock, dn-*iso*-OPDA, and Δ^4^-dn-*iso*-OPDA treated plants at the indicated concentration (Tukey’s HSD [honestly significant difference] ANOVA test; α = 0.05, *n* = 12–18). (C) Gene expression analysis by RT-qPCR in WT (Tak-1) and *Mpcoi1-2* with or without compounds (dn-*iso*-OPDA, Δ^4^-dn-*iso*-OPDA, 5 μM) treatment for 2 h. Error bars represent SD (*n* = 3). One-way analysis of variance (ANOVA) with post-hoc Tukey’s honestly significant difference (HSD) test (*p* < 0.05) analyses define the significant differences in gene expression.

The biological activity of Δ^4^-dn-*iso*-OPDA was examined in a growth inhibition assay, a typical jasmonate response. The induction of *Marchantia* jasmonate-signaling marker genes, such as Mp*DIR* (Dirigent-like)^37^ and Mp*PAT* (Patatin-like),^38^ were also tested. Δ^4^-dn-*iso*-OPDA and dn-*iso*-OPDA effectively inhibited the growth of *M. polymorpha* WT (Tak-1) at 5 and 10 μM, and their inhibitory effect was impaired in *Mpcoi1-2* mutant line (Figures 4A, 4B, and S3).^14^ In addition, quantitative reverse-transcription PCR (RT-qPCR) analyses of JA-marker gene (Mp*DIR* and Mp*PAT*) expression demonstrated that 5 μM of Δ^4^-dn-*iso*-OPDA and dn-*iso*-OPDA effectively upregulated their expressions. In contrast, their effects were impaired in the *Mpcoi1-2* mutant line (Figures 4C and S4).^14^ These results show that Δ^4^-dn-*iso*-OPDA activates the jasmonate-mediated responses in a MpCOI1-dependent manner, similar to the bioactive dn-*iso*-OPDA. However, as observed for dn-*iso*-OPDA in the previous studies,^14^^,^^38^ the growth inhibition by Δ^4^-dn-*iso*-OPDA was not completely abolished in *Mpcoi1-2* (Figures 4B and S3), indicating the existence of an MpCOI1-independent mode of action of Δ^4^-dn-*iso*-OPDA.

### Δ^4^-dn-*iso*-OPDA is a ligand of the MpCOI1-MpJAZ co-receptor

**Figure 5:**
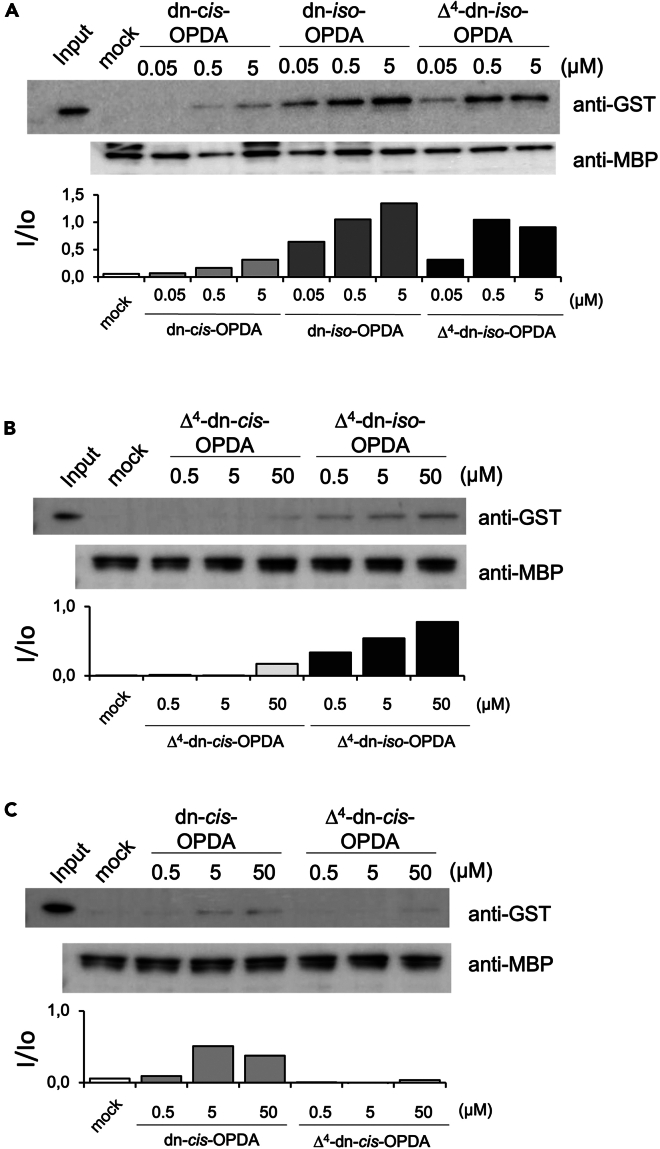
Affinity of Δ^4^-dn-OPDAs with MpCOI1-MpJAZ co-receptor GST-MpCOI1was incubated with MBP MpJAZ with (A) dn-*cis*-OPDA, dn-*iso*-OPDA, Δ^4^-dn-*iso*-OPDA, (B) Δ^4^-dn-*cis*-OPDA and Δ^4^-dn-*iso*-OPDA, and (C) dn-*cis*-OPDA and Δ^4^-dn-*cis*-OPDA in the pull-down buffer (the indicated concentrations). (Upper) Immunoblot with anti-GST-HRP antibody. (Lower) Immunoblot with anti-MBP and anti-IgG-HRP antibodies. Quantitative data of each blot were shown below the del image.

Next, we assessed the affinity of synthetic Δ^4^-dn-*iso*-OPDA for the MpCOI1-MpJAZ co-receptor using a pull-down assay with glutathione-*S*-transferase (GST)-tagged MpCOI1 and maltose binding protein (MBP)-tagged MpJAZ (Figure S5).^23^^,^^24^^,^^39^^,^^40^ As shown in Figure 5A, Δ^4^-dn-*iso*-OPDA induces the MpCOI1-MpJAZ interaction in a concentration-dependent manner (0.05–5 μM). We also compared the affinity of Δ^4^-dn-*iso*-OPDA for MpCOI1-MpJAZ with that of dn-OPDAs (dn-*iso*/*cis*-OPDAs), previously reported bioactive hormones (Figure 5A).^14^ The affinities of Δ^4^-dn-*iso*-OPDA and dn-*iso*-OPDA for MpCOI1-MpJAZ were similar (Figures 5A, S6A, and S6D). The affinity of Δ^4^-dn-*iso*-OPDA was approximately 100-fold higher than that of dn-*cis*-OPDA. In addition, we compared the two isomers, Δ^4^-dn-*iso*-OPDA and Δ^4^-dn-*cis*-OPDA: the *iso*-isomer showed >100-fold higher affinity than the *cis*-isomer (Figures 5B, S6B, and S6E). A direct comparison of dn-*cis*-OPDA and Δ^4^-dn-*cis*-OPDA suggested that the affinity of Δ^4^-dn-*cis*-OPDA was much lower than that of dn-*cis*-OPDA (Figures 5C, S6C, and S6F). Our current results define the order of affinities of the dn-OPDA series plant hormone of *Marchantia* as Δ^4^-dn-*iso*-OPDA ≈ dn-*iso*-OPDA ≫ dn-*cis*-OPDA > Δ^4^-dn-*cis*-OPDA. Overall, these results show that Δ^4^-dn-*iso*-OPDA functions as a bioactive jasmonate in *M. polymorpha*.

### Docking study of Δ^4^-dinor-*iso*/*cis*-OPDA against MpCOI1-MpJAZ co-receptor

**Figure 6:**
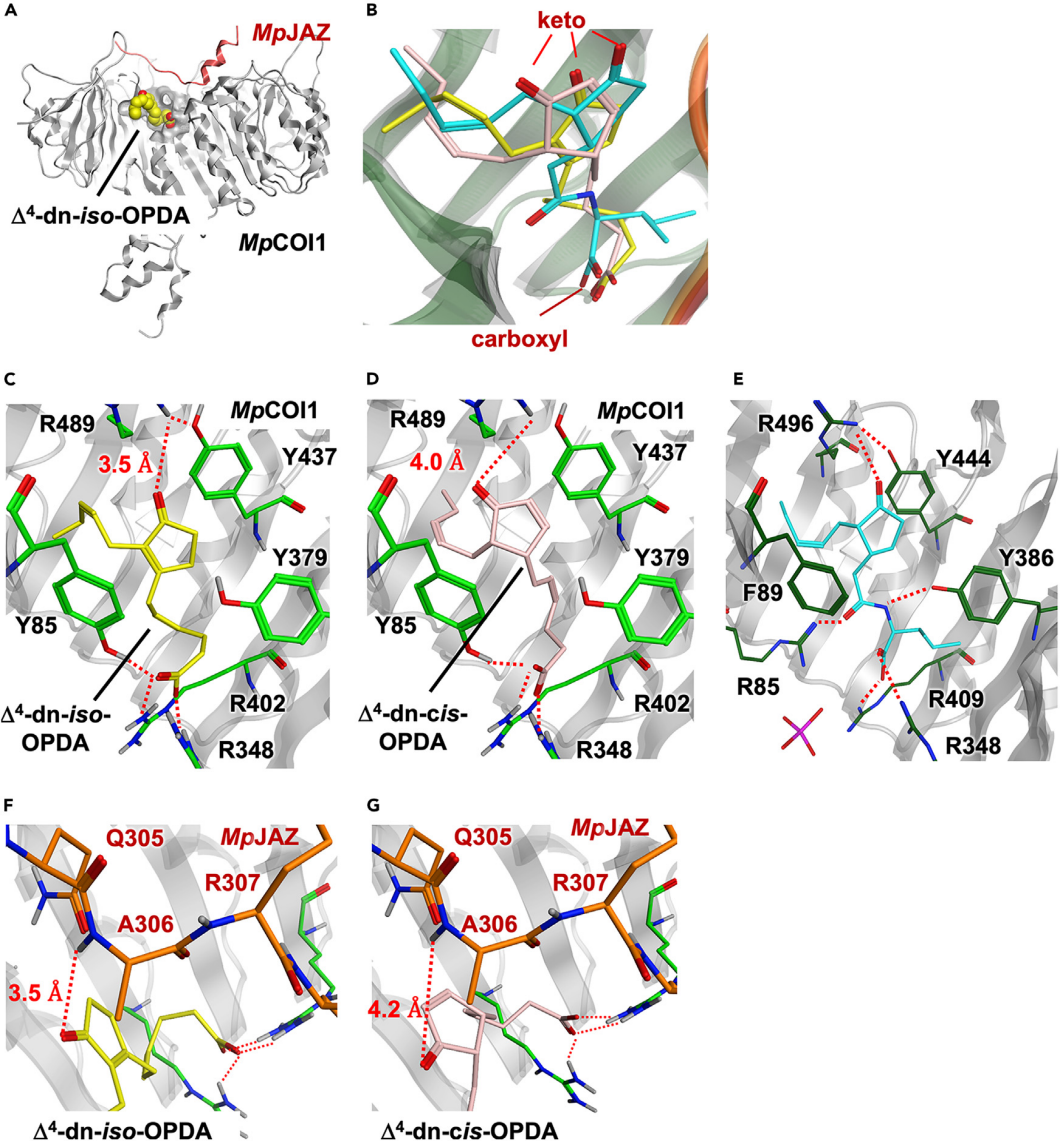
Docking structure of the MpCOI1-MpJAZ and Δ^4^-dn-*iso*-OPDA (A) Side view of the docking structure of the MpCOI1-MpJAZ (gray and red ribbons, respectively) and Δ^4^-dn-*iso*-OPDA in yellow space-filling representation. (B) Expanded views of the ligand binding sites: docking structure models of Δ^4^-dn-*iso*-OPDA (yellow) and Δ^4^-dn-*cis*-OPDA (pink) with MpCOI1-MpJAZ (gray), and crystal structures (3OGL) of JA-Ile (cyan) with AtCOI1-AtJAZ (green and orange ribbons, respectively). Expanded side view of the COI1-ligand binding interface of (C) Δ^4^-dn-*iso*-OPDA (yellow) and (D) Δ^4^-dn-*cis*-OPDA (pink) binding models in stick representation. Red dash bond indicates the potential hydrogen bond. Black letter indicates the label of amino acid residue of MpCOI1. (E) Side view of the hydrogen bonding network between JA-Ile and AtCOI1 in the crystal structure (3OGL). Expanded top view of the MpJAZ (orange)-ligand binding interface of (F) Δ^4^-dn-*iso*-OPDA (yellow) and (G) Δ^4^-dn-*cis*-OPDA (pink) binding models in stick representation. Red dash bond indicates the potential hydrogen bond. Brown letter indicates the label of amino acid residue of MpJAZ.

To understand the observed difference in ligand binding affinity of MpCOI1-MpJAZ, we performed a docking simulation of Δ^4^-dn-*iso*/*cis*-OPDA against a homology model of the MpCOI1-MpJAZ co-receptor, which was generated from the crystal structure of *Arabidopsis* COI1-JA-Ile-JAZ1 complex (PDB ID: 3OGL)^41^ (Figures 6A, S7A, and S7B). In the binding pocket of our docking model, both Δ^4^-dn-*iso*/*cis*-OPDA sit in an “upright” position with the keto group of the cyclopentenone ring at the pocket entrance and carboxyl groups bound to the bottom of the binding site, as seen in the crystal structure of COI1-JA-Ile-JAZ1 of *Arabidopsis* (Figures 6A, S7A, and S7B).

Comparing the arrangement of Δ^4^-dn-*iso*/*cis*-OPDA in our docking model with that of JA-Ile in the crystal structure of *Arabidopsis* COI1-JA-Ile-JAZ1 complex, the position of carboxyl moiety forming hydrogen bond network at the bottom-side was unchanged primarily (Figure 6B). In our docking model, the carboxyl moiety of Δ^4^-dn-*iso*/*cis*-OPDA includes a hydrogen bond network with Arg348/402 and Tyr85 (Figures 6C and 6D). A hydrogen bond network with Arg348/402 is the same pattern as seen in the crystal structure of the COI1-JA-Ile-JAZ1 complex (Figure 6E).^41^ However, in the COI1-JA-Ile-JAZ1 complex, an additional hydrogen bond between the amide moiety of JA-Ile and Tyr386 reinforces the interaction (Figure 6E). Instead, in our docking model of the MpCOI1-ligand-MpJAZ complex (Figures 6C and 6D), another hydrogen bond is formed between the carboxyl moiety of the ligands and Tyr85 which corresponds to Phe89 in AtCOI1, attracting Tyr85 close to the cyclopentenone ring of the ligand and forming the hydrophobic ligand interaction pocket surrounded by aromatic rings of Tyr85/Ty379/Tyr437 in MpCOI1 (Figures 6C and 6D).

In contrast, a significant difference was observed for the position of the keto moiety interacting with MpJAZ1 (Figure 6B). In the ligand-binding pocket of MpCOI1, the keto group of Δ^4^-dn-*iso*-OPDA forms an additional hydrogen bond (3.5 Å) with Arg489 of MpCOI1 (Figure 6C), enhancing affinity, whereas this hydrogen bond is weak (4.0 Å) in Δ^4^-dinor-*cis*-OPDA (Figure 6D). The keto moiety of Δ^4^-dn-*iso*-OPDA in the ligand binding pocket was located close enough to form a hydrogen bond with MpJAZ (3.5 Å), though the distance between Δ^4^-dinor-*cis*-OPDA and MpJAZ was expected to be more distant (4.2 Å, Figures 6F and 6G). These models may explain the stronger binding affinity of Δ^4^-dn-*iso*-OPDA compared to that of Δ^4^-dn-*cis*-OPDA.

### Δ^4^-dn-*iso*-OPDA is biosynthesized through isomerization of Δ^4^-dn-*cis*-OPDA *in M. polymorpha*

**Figure 7:**
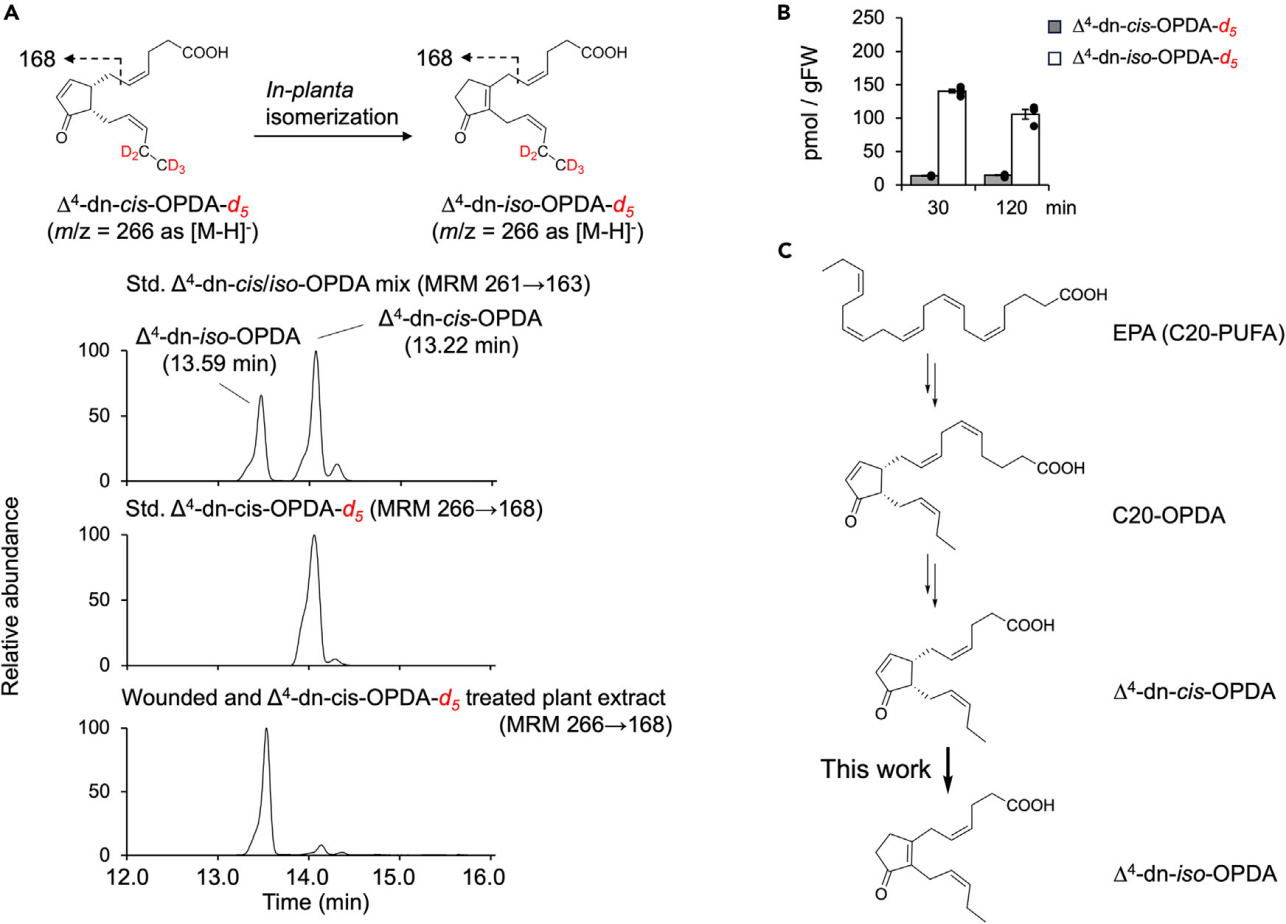
The *cis*-to-*iso* isomerization of D^4^-dn-*cis*-OPDA-*d*_*5*_ (A) UPLC-MS/MS analysis of *cis*-to-*iso* isomerization of Δ^4^-dn-*cis*-OPDA-*d*_*5*_ in *M. polymorpha.* (left) Precursor ion (*m/z*) 266 and fragment ion (*m/z*) 168 was used to monitor Δ^4^-dn-*cis*-OPDA-*d*_*5*_. (right) UPLC-MS/MS chromatogram of (top) synthetic Δ^4^-dn-*cis/iso*-OPDA standards in comparison with (middle) synthetic Δ^4^-dn-*cis*-OPDA-*d*_*5*_ and (bottom) the Δ^4^-dn-OPDA-*d*_*5*_ extracted from Δ^4^-dn-*cis*-OPDA-*d*_*5*_-treated *M. polymorpha* at 30 min after wounding. For HPLC condition, ZORBAX Eclipse Plus C18 column (1.8 μm, 2.1 × 50 mm) and aq. MeCN with 0.1% formic acid was used for eluent. For detection and quantitative analyses, we used a multiple reaction monitoring (MRM) in the negative-ion mode. (B) *In planta* conversion from Δ^4^-dn-*cis*-OPDA-*d*_*5*_ to Δ^4^-dn-*iso*-OPDA-*d*_*5*_ (30 min and 120 min after administration with wounding). (C) Proposed model for the biosynthesis of hormone in *M. polymorpha.*

The biosynthetic pathway of Δ^4^-dn-*iso*-OPDA remains unidentified. Upon wounding, *M. polymorpha* first accumulated Δ^4^-dn-*cis*-OPDA, followed by a concurrent increase of Δ^4^-dn-*iso*-OPDA and a decrease of Δ^4^-dn-*cis*-OPDA.^31^ The observed accumulation dynamic suggests the possible isomerization of Δ^4^-dn-*cis*-OPDA, which is biosynthesized from C20-PUFA, to Δ^4^-dn-*iso*-OPDA (Figure 1C). To test this hypothesis, we synthesized Δ^4^-dn-*cis*-OPDA-d_5_ (Figure S8) and examined the wound-induced isomerization *in planta*. Upon wounding, *M. polymorpha* was immediately treated with Δ^4^-dn-*cis*-OPDA-d_5_, and the plant materials were extracted 30 and 120 min after wounding. Subsequent multiple reaction monitoring (MRM) analyses on UPLC-MS/MS demonstrated that the peak of *m*/*z* = 168 from [M-H]^-^ (*m*/*z* = 266) observed at R_t_ = 12.80 min reduced after wounding, and a new peak of *m*/*z* = 168 appeared at R_t_ = 12.25 min, confirming that isomerization of Δ^4^-dn-*cis*-OPDA-d_5_ into Δ^4^-dn-*iso*-OPDA-d_5_ occurred in *M. polymorpha* (Figures 7A and S9). Our result showed that ca. 90% of Δ^4^-dn-*cis*-OPDA-d_5_ was converted into Δ^4^-dn-*iso*-OPDA-d_5_ within 30 min and reached a plateau (*cis*: *iso* = 1 : 9.6), suggesting the previously observed biological activity of Δ^4^-dn-*cis*-OPDA could be attributed to the converted Δ^4^-dn-*iso*-OPDA.^31^ Considering the weak affinity to the MpCOI1-MpJAZ co-receptor, Δ^4^-dn-*cis*-OPDA can be a biosynthetic precursor of a genuine hormone, Δ^4^-dn-*iso*-OPDA, in *M. polymorpha*.

### Distribution of Δ^4^-dn-OPDAs in bryophyte lineages

**Figure 8:**
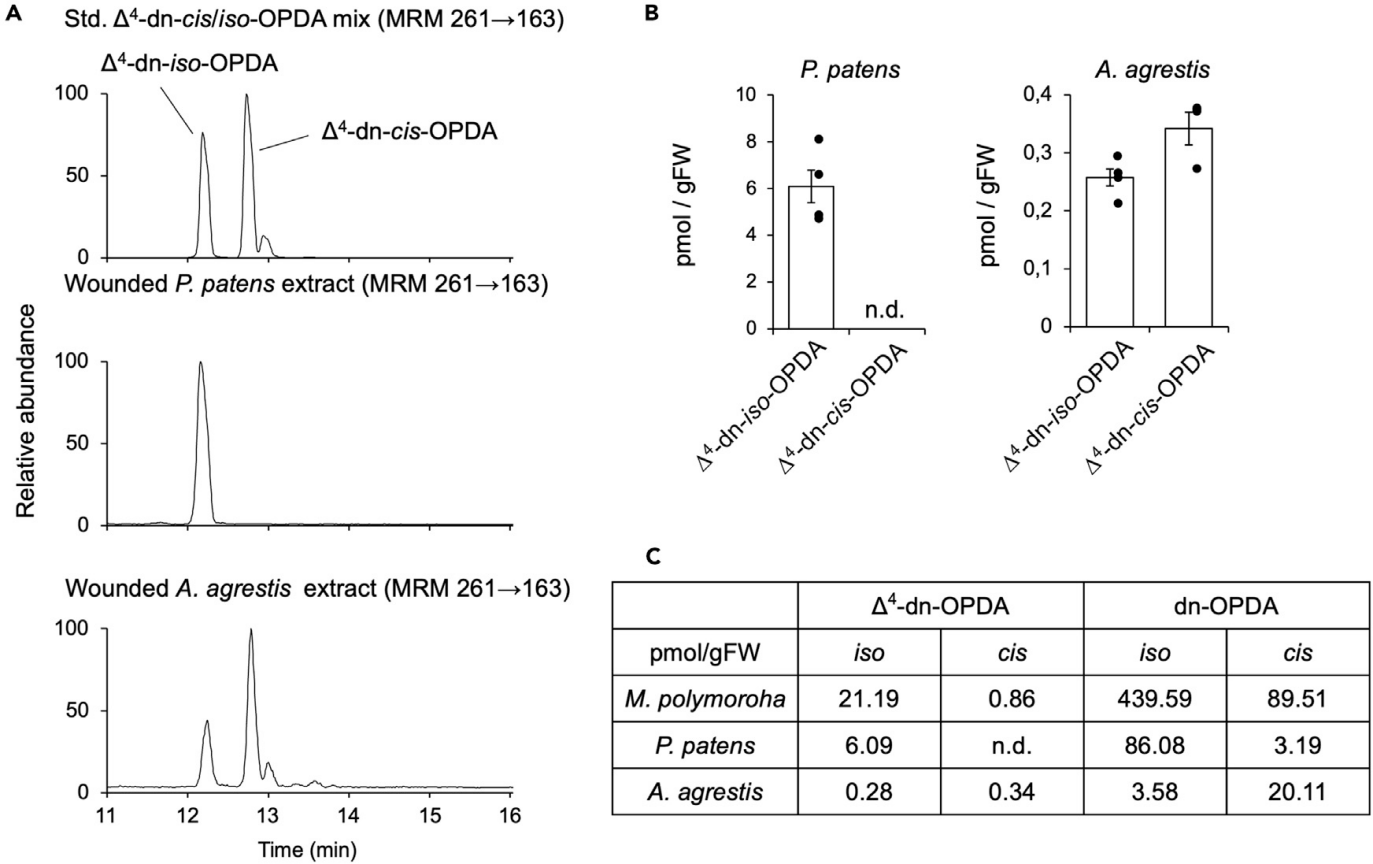
Δ^4^-dn-*cis*/*iso*-OPDA were detected in *P patens* and *A agrestis* (A) (right) UPLC-MS/MS chromatogram of (top) synthetic Δ^4^-dn-*cis*/*iso*-OPDA standards in comparison with (middle) the Δ^4^-dn-OPDA extracted from *P. patens* at 120 min after wounding and (bottom) the Δ^4^-dn-OPDA extracted *A. agrestis* at 120 min after wounding. For HPLC condition, ZORBAX Eclipse Plus C18 column (1.8 μm, 2.1 × 50 mm) and aq. MeCN with 0.1% formic acid was used for eluent. For detection and quantitative analyses, we used a multiple reaction monitoring (MRM) in the negative-ion mode. (B) The amount of Δ^4^-dn-OPDA detected in *P. patens* and *A. agrestis* (120 min after wounding). (C) The amount of Δ^4^-dn-OPDA and dn-OPDA in *M. polymorpha*, *P. patens*, and *A. agrestis* (pmol/gFW, 120 min after wounding).

Bryophytes are divided into liverworts, mosses, and hornworts;^42^^,^^43^^,^^44^ *M. polymorpha*, in which Δ^4^-dn-*cis*/*iso*-OPDA was detected, belongs to the liverworts (Figure 1B). The previous oxylipin analysis showed that the distribution of Δ^4^-dn-OPDAs in the plant lineage is limited to bryophytes, since Δ^4^-dn-OPDA were found only in *M. polymorpha* and a moss *Physcomitrium formosum*.^27^ And another non-targeted metabolome analysis of a moss *Physcomitrium patens* showed that no accumulation of C20-derived oxylipin was observed in wounded *P. patens*.^45^ Now, we have synthetic Δ^4^-dn-*cis*/*iso*-OPDA in hand, thus, we analyzed the existence of Δ^4^-dn-*cis*/*iso*-OPDA in a moss *P. patens* and a hornwort *Anthoceros agrestis* to confirm their distribution in bryophyte lineages (Figure 1B).^46^^,^^47^^,^^48^ MRM analyses on UPLC-MS/MS using a larger amount of plant samples (ca. 900 mg of *P. patens* and ca. 3000 mg of *A. agrestis*) than in previous studies showed that Δ^4^-dn-*iso*-OPDA (6.09 pmol/gFW) was detected in *P. patens* and Δ^4^-dn-*cis*/*iso*-OPDA (*cis*: 0.28 pmol/gFW, *iso*: 0.34 pmol/gFW) in *A. agrestis*, although at lower levels (Figures 8A–8C and S9). These results indicated that Δ^4^-dn-OPDAs are more widely distributed in bryophytes than previously examined.

## Discussion

Unique ligand-receptor co-evolution has been disclosed for the combination of jasmonate and the COI1-JAZ co-receptor system.^14^ The bryophyte model plant *M. polymorpha* uses dn-OPDAs, instead of JA-Ile exploited by angiosperms, as a ligand of COI1-JAZ co-receptor to induce jasmonate responses. During evolution, dn-OPDAs were replaced by JA-Ile, which accompanies mutations of amino acid residues in the ligand-interacting motif of COI1 and JAZ proteins, from Val-containing COI1^V^, such as MpCOI1^V377^ in *M. polymorpha*, to Ala-containing COI1^A^, such as AtCOI1^A384^ in *A. thaliana*, and from JAZ^EQ^, such as MpJAZ^E200Q203^ in *M. polymorpha*, to AtJAZ, such as AtJAZ3^A200L203^ in *A. thaliana* (Figure 1B).^26^^,^^27^ Based on this finding, a fascinating model for the evolution of plant hormone signaling module was proposed in which evolution occurs first in COI1 and then in JAZ, resulting in a stepwise change of the ligand from dn-*cis*/*iso*-OPDA to JA-Ile.^26^ In addition, a previous study disclosed the involvement of an additional jasmonate in *M. polymorpha*. Δ^4^-dn-*iso*-OPDA was proposed as the bioactive isomer; however, a formal demonstration was missing.

Here, we showed that the major isomer of Δ^4^-dn-OPDAs in *M. polymorpha* is Δ^4^-dn-*iso*-OPDA studying its wound-induced natural occurrence in *M. polymorpha*, its *in vitro* affinity for the COI1-JAZ co-receptor of *M. polymorpha* (MpCOI1-MpJAZ), and its induction of growth inhibition and marker gene expression on *M. polymorpha.* Previous studies showed a different dynamic of the accumulation of Δ^4^-dn-OPDA isomers: Δ^4^-dn-*cis*-OPDA was detected within 5 min after wounding, earlier than Δ^4^-dn-*iso*-OPDA. In addition, its accumulation remains much lower than that of Δ^4^-dn-*iso*-OPDA, indicating the conversion of *cis*-to-*iso* occurred in plants.^31^ Here, we showed the naturally occurring conversion of Δ^4^-dn-*cis*-OPDA into Δ^4^-dn-*iso*-OPDA in *M. polymorpha* (Figure 7). Considering the weak affinity of Δ^4^-dn-*cis*-OPDA for MpCOI1-MpJAZ co-receptor (Figure 5B), these results suggest that the main biological role of Δ^4^-dn-*cis*-OPDA as a biosynthetic precursor of a genuine bioactive hormone Δ^4^-dn-*iso*-OPDA (Figure 7B). Our current result indicated that the previously observed biological activities of Δ^4^-dn-*cis*-OPDA^31^ could be attributed to the Δ^4^-dn-*iso*-OPDA resulting from *in planta cis*-to-*iso* conversion.

Isomerization from *cis*-to *iso*-helps to increase the stability of the molecule in the cellular environment and enhances its usefulness as a hormone. The disubstituted α, β-unsaturated ketone moiety of Δ^4^-dn-*cis*-OPDA in the cyclopentenone ring functions as a Michael acceptor *in vivo*, reacting with nucleophiles, such as glutathione, resulting in inactivation.^43^^,^^44^^,^^49^ In *Arabidopsis thaliana*, the structurally similar molecule *cis*-OPDA of α, β-unsaturated ketone moiety is used as the biosynthetic precursor of jasmonic acid.^20^ *cis*-OPDA exists primarily in organelles such as plastid and peroxisome, separated from the cytosolic environment, and is rapidly reduced by 12-oxophytodienoate reductase 3 (OPR3) reductase to avoid Michael addition.^20^^,^^50^ In contrast, Δ^4^-dn-*iso*-OPDA has a tetrasubstituted α, β-unsaturated ketone moiety in the cyclopentenone ring and would be stable *in vivo* without being consumed by Michael addition. Therefore, Δ^4^-dn-*iso*-OPDA would be evolutionarily developed as a stable endogenous ligand of the MpCOI1-MpJAZ co-receptor.

Comparison between Δ^4^-dn-*iso*-OPDA and dn-*iso*-OPDA, a previously identified bioactive jasmonate,^14^ is important for understanding their biological role. Previous studies demonstrated that MpCOI1 and MpJAZ share the biological and molecular functions of their *Arabidopsis* counterparts, functioning in *M. polymorpha* through the ligand-induced formation of the MpCOI1-MpJAZ co-receptor.^14^^,^^15^ Δ^4^-dn-*iso*-OPDA bound to MpCOI1-MpJAZ co-receptor as strong as dn-*iso*-OPDA and it was as effective as dn-*iso*-OPDA for the growth inhibition and induction of JA marker gene expression (Figures 4A–4C). Bioassays with Mp*coi1* mutant lines showed that the activity of Δ^4^-dn-*iso*-OPDA was MpCOI1-dependent, but the growth inhibition caused by Δ^4^-dn-*iso*-OPDA was not completely abolished in Mp*coi1-2* (Figures 4B and S3). Since previous studies have reported that the growth inhibitory activity of dn-OPDAs is not completely abolished in Mp*coi1*,^14^^,^^38^ it is likely that there is an MpCOI1-independent mode of action for Δ^4^-dn-*iso*-OPDA and dn-OPDAs. However, considering the accumulation of Δ^4^-dn-*iso*-OPDA (ca. 120–130 pmol/g) after wounding is significantly lower than that of dn-*iso*-OPDA (ca. 4000 pmol/g),^31^ we conclude that Δ^4^-dn-*iso*-OPDA is a unique C20-PUFA-derived hormone for the defense response of *M. polymorpha* that acts subsidiarily to dn-*iso*-OPDA. As in the case of Δ^4^-dn-OPDA, dn-*iso*-OPDA has stronger co-receptor affinity and higher accumulation after wounding than dn-*cis*-OPDA, but the direct conversion from deuterium-labeled dn-*cis*-OPDA to deuterium labeled dn-*iso*-OPDA has not been investigated yet.^14^^,^^51^^,^^52^ Several previous studies have reported the PUFA content of *M. polymorpha*.^30^^,^^53^^,^^54^ While the specific contents varied across these studies, it was consistently found that the C20-PUFA content equaled or exceeded that of C16/C18-PUFA. This finding suggests that the accumulation of dn-OPDAs over Δ^4^-dn-OPDAs in *M. polymorpha* cannot be attributed to differences in the levels of their biosynthetic precursors, C20-PUFA and C16/C18-PUFA. Instead, it implies that oxylipin synthesis is severely regulated in *M. polymorpha*.

Bryophytes are divided into liverworts, mosses, and hornworts;^42^^,^^43^^,^^44^ *M. polymorpha*, in which Δ^4^-dn-*cis*/*iso*-OPDA was detected, belongs to the liverworts (Figure 1B). In this work, we demonstrated that a moss *P*. *patens* and a hornwort *A. agrestis* accumulated a small amount of Δ^4^-dn-OPDAs upon wounding (Figures 8 and S10). Our current result overrides the results in the previous two reports: non-targeted metabolome analysis of *P. patens* in which *P. patens* does not accumulate Δ^4^-dn-OPDAs upon wounding,^45^ and accumulation of Δ^4^-dn-OPDAs was not observed in *P. patens* but in *M. polymorpha* and *P. formosum*.^27^ In this experiment, we believe that current success in detecting Δ^4^-dn-OPDAs is due to two main factors. Firstly, the use of a larger amount of plant samples compared to previous experiments, and secondly, the use of synthetic standards of Δ^4^-dn-*iso*-OPDA, a major isomer, in the fine-tuned UPLC-MS/MS analysis. This result suggests that Δ^4^-dn-OPDAs may be more widespread in bryophytes than previously considered (Figure 1B), and the pathway for the biosynthesis of plant oxylipins from C-20 PUFAs may be widely distributed in bryophytes. The unique occurrence of C20-derived cyclopentenones, including Δ^4^-dn-OPDAs in bryophytes, may provide important insights into the evolution of plant hormones.

### Limitations of the study

The results shown here encourage us to identify the putative *Marchantia* isomerase responsible for the isomerization of Δ^4^-dn-*cis*-OPDA into Δ^4^-dn-*iso*-OPDA. Considering the absence of *iso*-OPDA and dn-*iso*-OPDA in *A. thaliana,*^14^^,^^55^ the isomerase could be a unique feature of bryophytes. The acquisition of isomerase catalyzing the conversion of Δ^4^-dn-*cis*-OPDA to Δ^4^-dn-*iso*-OPDA could be a critical evolutional event to use Δ^4^-dn-OPDAs as jasmonate hormones. Thus, identifying this putative isomerase may provide an essential clue for a deeper understanding of plant hormone evolution.

### Methods

All methods can be found in STAR methods and the accompanying supplemental information file.

## STAR★Methods

### Key resources table

**Table:** 

REAGENT or RESOURCE	SOURCE	IDENTIFIER
**Antibodies**		

Anti-GST HRP Conjugate	Cytiva	Cat # RPN1236; RRID: AB_771429
Anti-MBP, Monoclonal Antibody, Rat	Wako	Cat # 016-24141
goat anti-rat IgG-HRP	Santa Cruz Biotechnology	Cat # Sc-2032; RRID: AB_631755

**Bacterial and virus strains**		

*E. coli* DH10Bac	Thermo Fisher	Cat # 10361012
*E. coli* OmniMAX™	Thermo Fisher	Cat #C854003
*E. coli* BL21(DE3)	New England BioLabs	Cat #C2527

**Chemicals, peptides, and recombinant proteins**		

The list of compounds described in Figures 2A and S8.	Refer to STAR methods and supplemental information	N/A
dn-*cis*-OPDA	Wang et al., *Sci.Rep. 11*, 2033 (2021).^35^	N/A
dn-*iso*-OPDA	Wang et al., *Sci.Rep. 11*, 2033 (2021).^35^	N/A
Δ^4^-dn-*cis*-OPDA	Kneeshaw et al. *Proc.Natl.Acad.Sci. 119*, e2202930119 (2022).^31^	N/A
Recombinant GST-*Mp*COI1-*At*ASK1	Refer to STAR methods and supplemental information.	Addgene #29517 (pFB-HTB-ASK1)
Acetonitrile -Plus-	KANTO CHEMICAL	Cat # 01031-2B
Amylose Resin	BioLabs	Cat #E8021
AZADOL®	Wako	Cat # 010–24921; CAS: 1155843-79-0
1-Bromopropane-2,2,3,3,3-d5	KANTO CHEMICAL	Cat # 49122-56
Chlorotriethylsilane	TCI	Cat #T0589; CAS: 994-30-9
Chloroform	Wako	Cat # 038–02606; CAS: 67-66-3
Chromium(VI) Oxide	Wako	Cat # 037–03232; CAS: 1333-82-0
Copper (II) Bromide	TCI	Cat #C2389; CAS: 7789-45-9
Cyclohexene	TCI	Cat #C0491; CAS: 110-83-8
(Diacetoxyiodo)benzene	Sigma-Aldrich	Cat # 178721-25G; CAS: 3240-34-4
Dichloromethane, dehydrated -Super^2^-	KANTO CHEMICAL	Cat # 11338-84; CAS: 75-09-2
Diisobutylaluminum Hydride (17% in Toluene, ca. 1.0 mol/L)	TCI	Cat #D2972; CAS: 1191-15-7
Dimethyl Sulfoxide, Super Dehydrated	Wako	Cat # 048–32811; CAS: 67-68-5
D-myo-inositol-1,2,4,5,6-pentaphosphate, sodium salt	Cayman chemical	Cat # 10008452
ECL Prime Western Blotting System	Cytiva	Cat # RPN2232
ESF921 medium	Funakoshi	Cat # 96-001-01
Ethyl acetate	Nacalai	Cat # 14622-14; CAS: 141-78-6
Factor Xa protease	BioLabs	Cat #P8010
FBS	biosera	Cat # 556-33865
Formic acid (abt.99%)	Wako	Cat # 063-04192
Gamborg’s B5 Medium Salt Mixture	Wako	Cat # 399-00621
Glutathione Sepharose 4B	Cytiva	Cat # GE17-0756-01
Grace’s Insect Medium, supplemented	Thermo Fisher	Cat # 11605094
Hexane	Wako	Cat # 080-00427
Imidazole	Wako	Cat # 099-00013; CAS: 288-32-4
MagExtractor™ -PCR&Gel Clean up-	TOYOBO	Cat # NPK-601
Methanol	Japan Alcohol Corporation	CAS: 67-56-1
Methyl Jasmonate	Wako	Cat # 133–14412; CAS: 1211-29-6
Methyl Propionate	TCI	Cat #P0508; CAS: 554-12-1
*N, N*-Dimethylformamide, Super Dehydrated	Wako	Cat # 043–32361; CAS: 68-12-2
Oxalyl Chloride	TCI	Cat #O0082; CAS: 79-37-8
Potassium bis(trimethylsilyl)amide solution - 0.5 M in Toluene		
Sodium Thiosulfate Pentahydrate	Nacalai	Cat # 32005-44; CAS: 10102-17-7
Sodium bis(trimethylsilyl)amide solution – 1.0 M in THF	Sigma-Aldrich	Cat # 245585-100ML; CAS: 1070-89-9
Sulfuric Acid	Wako	Cat # 192–04696; CAS: 7664-93-9
TBDPSO(CH_2_)_4_PPh_3_I	Kang et al. *Tetrahedron Lett.36*, 5049 (1995).	N/A
Tetrabutylammonium Fluoride (ca. 1 mol/L in Tetrahydrofuran)	TCI	Cat #T1338; CAS: 429-41-4
Tetrahydrofuran, dehydrated stabilizer free -Super Plus-	KANTO CHEMICAL	Cat # 41001-85; CAS: 109-99-9
Triethylamine	Wako	Cat # 202–02646; CAS: 121-44-8
Triphenylphosphine	Wako	Cat # 204–03061; CAS: 603-35-0
ViaFect™ Transfection Reagent	Promega	Cat #E4981

**Critical commercial assays**		

baculoQUANT™ ALL-IN-ONE Virus Extraction&Tirtration Kit	Oxford Expression Technologies	Cat # 100602

**Experimental models: Cell lines**		

Sf9 insect cells	Oxford Expression Technology	Cat # 600100

**Experimental models: Organisms/strains**		

*Marchantia polymorpha* Tak-1	Monte et al. *Nat.Chem.Biol. 14*, 480 (2018).^14^	N/A
*Mpcoi1-2* mutant	Monte et al. *Nat.Chem.Biol. 14*, 480 (2018).^14^	N/A

**Recombinant DNA**		

pKM596-MpJAZ	Monte et al. *Nat.Chem.Biol. 14*, 480 (2018).^14^	N/A
pFB-GTE-MpCOI1	This paper	Prepared from Addgene #29516 (pFB-GTE-COI1), pMpGWB311-35S::MpCOI1-FLAG (Monte et al. *Nat.Chem.Biol. 14*, 480 (2018).)

**Software and algorithms**		

ChemDraw Professional 18.0	PerkinElmer	https://www.perkinelmer.com/category/chemdraw
MOE v2020.09	Chemical Computing Group	https://www.chemcomp.com/Products.htm
Delta 6.1.0	JEOL	https://nmrsupport.jeol.com/
ColabFold v1.5.3	Mirdita et al. *Nature Methods 19*, 679 (2022).^66^	https://colab.research.google.com/github/sokrypton/ColabFold/blob/v1.2.0/AlphaFold2.ipynb
Spectra Manager Version 2	JASCO	N/A
Compass 1.3 for micrOTOF – SR 1	Bruker	N/A
ALICE2 for Windows Version 5.9	JEOL	N/A
ImageJ	NIH	https://imagej.nih.gov/ij/

**Other**		

NanoPhotometer® N60	IMPLEN	https://www.implen.de/product-page/implen-nanophotometer-n60-microvolume-spectrophotometer
Amersham Imager 680 detector	Cytiva	https://www.cytivalifesciences.co.jp/catalog/43565.html
JNM-ECS-400 spectrometer	JEOL	https://www.jeol.co.jp/products/scientific/nmr/JNM-ECS.html
FT/IR-4100	JASCO	https://www.jasco.co.jp/jpn/product/FTIR/FTIR.html
microTOF II	Bruker Daltonics	N/A
JASCO P-2200 polarimeter	JASCO	https://www.jasco.co.jp/jpn/product/Polarimeter/spec.html
Isolera™ One	Biotage	https://www.biotage.co.jp/products_top/flash-purification/isolera_top/
Optima MAX-XP	BECKMAN COULTER	https://www.beckman.jp/centrifuges/ultracentrifuges/optima-max-xp#
GENE PERP STAR PI-80X	KURABO	N/A
Agilent 1290 Infinity II + Ultivo Triple Quad LC/MS	Agilent	https://www.chem-agilent.com/contents.php?id=1004748
LPH-241SP	NIPPON MEDICAL&CHEMICAL INSTRUMENTS	https://www.nihonika.co.jp/en/e/product/4422.html

### Resource availability

#### Lead contact

Further information and requests for resources and reagents should be directed to and will be fulfilled by the lead contact, Minoru Ueda (minoru.ueda.d2@tohoku.ac.jp).

#### Materials availability

This study did not generate new unique reagents.

#### Data and code availability

• All data reported in this paper will be shared by the lead contact upon request.
• This paper does not report the original code.
• Any additional information required to reanalyze the data reported in this paper is available from the lead contact upon request.

### Experimental model and subject details

#### Sf9 culture method

Sf9 cells used for GST-MpCOI1 expression were grown at 27°C with 127 rpm in ESF921 medium matrix (2% FBS, 0.5% Penicillin-Streptomycin, 0.5 ng/mL Amphotericin B).

#### *E. coli* culture method

*E. coli* BL21 (DE3) strain cells used for the expression of MBP-MpJAZ were grown at 37°C with 250 rpm in liquid LB medium supplemented with 1 μg/mL Ampicillin.

#### Plant materials and growth conditions

*Marchantia polymorpha* accession Takaragaike-1 (Tak-1; male) was used as the wild-type (WT) and Mp*coi1-2* was used as the Mp*coi1* allele in this study. *M. polymorpha* plants were grown from gemmae on sterilized 0.5 Gamborg’s B5 medium with 0.5% or 1% agar in long-day conditions (50–60 μmol m^−2^ s^−1^, 16-h) at 21°C. *Physcomitrium patens* ssp. *patens* and *Anthoceros agrestis* Bonn strain were used.^56^^,^^57^ Protonemata of *P. patens* were grown on BCD medium^58^ with 0.8% agar under continuous light (50–60 μmol m^−2^ s^−1^) at 25°C. Thalli of *A. agrestis* were grown on KNOP medium^59^ with 0.75% gellan gum under continuous light (10–15 μmol m^−2^ s^−1^) at 22°C.

### Method details

#### General materials and methods

All chemical reagents and solvents were obtained from commercial suppliers (Wako Pure Chemical Industries Co. Ltd., Nacalai Tesque Co., Ltd., Watanabe Chemical Industries Co. Ltd., Thermo Fisher Scientific K.K., GE Healthcare) and used without further purification. Reversed-phase high–performance liquid chromatography (HPLC) was carried out on a PU–4180 plus pump equipped with UV–4075 and MD–4010 detectors (JASCO, Tokyo, Japan). Both ^1^H and ^13^C NMR spectra were recorded on JNM–ECS–400 spectrometers (JEOL, Tokyo, Japan) in deuterated chloroform and using TMS as an internal standard or in deuterated pyridine using the solvent peak as an internal standard. Fourier transforms infrared (FT/IR) spectra were recorded on an FT/IR–4100 (JASCO, Tokyo, Japan). High–resolution (HR) electrospray ionization (ESI)–mass spectrometry (MS) analyses were conducted using a microTOF II (Bruker Daltonics Inc., Billerica, MA). MALDI-TOF MS analysis was performed on an Autoflex II (Bruker Daltonics Inc., MA, US). All anhydrous solvents were either dried by standard techniques and freshly distilled before use or purchased in anhydrous form. Flash chromatography was performed on the Isolera system (Biotage Ltd., North Carolina, US). TLC was performed on Silica gel F254 (0.25 mm or 0.5 mm, MERCK, Germany). All reactions were carried out under air unless stated otherwise. SDS-PAGE and Western blotting were performed using Mini-Protean III (Bio-Rad Laboratories, Inc., US), Trans-Blot Turbo (Bio-Rad Laboratories, Inc., US), and iBind Flex (Thermo Fisher Scientific K.K., CA, US) devices. Chemiluminescent images were detected using the Amersham Imager 680 detector (GE Healthcare, CA, US).

All chemical reagents and solvents were obtained from commercial suppliers (Kanto Chemical Co. Ltd., Wako Pure Chemical Industries Co. Ltd., Nacalai Tesque Co. Ltd., Tokyo Chemical Industry Co. Ltd., Sigma-Aldrich Co. LLC., GE Healthcare) and used without further purification. All anhydrous solvents were either dried by standard techniques and freshly distilled before use or purchased in anhydrous form and used as supplied. Reversed-phase high–performance liquid chromatography (HPLC) was carried out on a PU–4180 plus pump equipped with UV–4075 and MD–4010 detectors (JASCO, Tokyo, Japan). ^1^H and ^13^C NMR spectra were recorded on a JNM–ECS–400 spectrometer (JEOL, Tokyo, Japan). Chemical shifts are denoted in δ (ppm) relative to TMS or residual solvent peaks as internal standard (TMS, ^1^H δ 0.00; CDCl_3_, ^13^C δ 77.0; C_6_D_6_, ^1^H δ 7.16, ^13^C δ 128.0; pyridine-*d5*, ^1^H δ 8.74, ^13^C δ 150.4). Fourier transforms infrared (FT/IR) spectra were recorded on an FT/IR–4100 (JASCO, Tokyo, Japan). High–resolution (HR) electrospray ionization (ESI)–mass spectrometry (MS) analyses were conducted using a microTOF II (Bruker Daltonics Inc., Billerica, MA). Optical rotations were measured using a JASCO P–2200 polarimeter (JASCO, Tokyo, Japan). Medium-pressure column chromatography was performed on an Isolera system (Biotage Ltd., North Carolina, US). TLC analyses were performed on Silica gel F254 (0.25 mm or 0.5 mm, MERCK, Germany) or RP–18F254S (0.25 mm, MERCK). All reactions were carried out under air unless stated otherwise.

#### Chemical syntheses of *Marchantia* jasmonates

Preparation of dn-*iso*/*cis*-OPDA^35^ and Δ^4^-dn-*cis*-OPDA^31^ was achieved according to the previous methods. The ^1^H- and ^13^C-NMR spectra of the synthetic intermediates are involved in supplemental information.

##### Synthesis of 3,7-ddh-MeJA (1)

To a solution of MeJA (4.96 g, 22.1 mmol) and cyclohexene (22.6 mL, 223 mmol) in CHCl_3_-Me propionate (18 mL, 1:1 (v/v)) was bubbled with Ar, then added CuBr_2_ (10.0 g, 44.8 mmol) at room temperature under an argon atmosphere. After being stirred at 80°C for 22 h, the reaction mixture was quenched with saturated aqueous NH_4_Cl and extracted with EtOAc. The resulting organic layer was washed with saturated aqueous NaCl, dried over Na_2_SO_4_, and filtered. After evaporation, the residue was purified by medium-pressure column chromatography on silica gel (*n*-hexane/EtOAc = 95/5 to 60/40) to give **1**^60^ (1.68 g, 34%) as an orange oil. ^1^H NMR (400 MHz, CDCl_3_) δ_H_: 5.41 (dtt, *J* = 10.6, 7.5, 1.8 Hz, 1H), 5.20 (dtt, *J* = 10.6, 7.3, 1.7 Hz, 1H), 3.73 (s, 3H), 3.48 (s, 2H), 2.97 (d, *J* = 7.5 Hz, 2H), 2.62 (brt, *J* = 4.5 Hz, 2H), 2.42 (td, *J* = 4.5, 2.3 Hz, 2H), 2.15 (quintet, *J* = 7.3 Hz, 2H), 0.99 (t, *J* = 7.3 Hz, 3H); ^13^C NMR (100 MHz, CDCl_3_) δ_C_: 208.8, 169.7, 164.2, 142.1, 133.3, 124.6, 52.6, 36.9, 34.5, 30.2, 21.6, 20.9, 14.4; IR (neat) cm^−1^: 2960, 1740, 1701, 1437, 1173; HRMS (ESI, positive) *m/z* [M+Na]^+^ Calcd. for C_13_H_18_NaO_3_: 245.1148, Found: 245.1149.

##### Synthesis of aldehyde 2

To a solution of **1** (1.00 g, 4.49 mmol) in CH_2_Cl_2_ (76 mL) was added DIBAL (22.6 mL, 1 M in toluene, 22.6 mmol) at −78°C under an argon atmosphere. After stirring at −78°C for 1 h, the reaction mixture was quenched with 1 M aqueous HCl and extracted with EtOAc. The resulting organic layer was washed with saturated aqueous NaCl, dried over Na_2_SO_4_, and filtered. After evaporation, the residue was purified by medium-pressure column chromatography on silica gel (*n*-hexane/EtOAc = 92/8 to 34/66) to give **2** (602 mg, 70%) as a yellow oil. ^1^H NMR (400 MHz, CDCl_3_) δ_H_: 9.63 (t, *J* = 2.3 Hz, 1H), 5.43–5.50 (m, 2H), 4.74–4.76 (m, 1H), 3.19–3.29 (m, 2H), 2.84–3.00 (m, 2H), 2.41–2.53 (m, 1H), 2.29–2.33 (m, 2H), 2.13 (quintet, *J* = 7.3 Hz, 2H), 0.99 (t, *J* = 7.3 Hz, 3H); ^13^C NMR (100 MHz, CDCl_3_) δ_C_: 199.0, 142.0, 133.5, 130.8, 125.7, 79.4, 44.5, 34.3, 32.8, 24.5, 20.9, 14.5; IR (neat) cm^−1^: 3396, 2962, 2933, 1721, 1456, 1018; HRMS (ESI, positive) *m/z* [2M+Na]^+^ Calcd. for C_24_H_36_NaO_4_: 411.2506, found: 411.2501.

##### Synthesis of 3

To a suspension of [TBDPSO(CH_2_)_4_PPh_3_]^+^I^-^^61^ (631 mg, 900 μmol) in THF (3.6 mL) was added KHMDS (0.5 M in toluene, 1.7 mL, 850 μmol). The mixture was stirred for 40 min and cooled to - 78°C. To this solution was added a solution of **2** (63.3 mg, 326 μmol) in THF (3.6 mL). The reaction mixture was gradually warmed to room temperature for 2 h. Then, the reaction was quenched with saturated aqueous NH_4_Cl and extracted with *n*-hexane. The organic layer was washed with saturated aqueous NaCl, dried over Na_2_SO_4_, and filtered. After evaporation, the residue was purified by medium-pressure column chromatography on silica gel (*n*-hexane/EtOAc = 95/5 to 60/40) to give **3** (58.4 mg, 37%) as a yellow oil. ^1^H NMR (400 MHz, pyridine-d_5_) δ_H_: 7.87–7.89 (m, 4H), 7.52–7.53 (m, 6H), 5.61–5.70 (m, 1H), 5.51 (dt, *J* = 11.9, 6.5 Hz, 2H), 5.44–5.56 (m, 1H), 5.03–5.18 (m, 1H), 3.82 (t, *J* = 6.5 Hz, 2H), 3.34 (dd, *J* = 14.7, 7.6 Hz, 1H), 3.15 (dd, *J* = 14.7, 7.6 Hz, 1H), 3.02 (d, *J* = 6.5 Hz, 2H), 2.44–2.51 (m, 1H), 2.32 (q, *J* = 6.5 Hz, 2H), 2.24 (quintet, *J* = 7.5 Hz, 2H), 2.20–2.37 (m, 2H), 1.92–2.02 (m, 1H), 1.75 (quintet, *J* = 6.5 Hz, 2H), 1.18 (s, 9H), 0.98 (t, *J* = 7.5 Hz, 3H); ^13^C NMR (100 MHz, pyridine-d_5_) δ_C_:138.9, 137.3, 136.5 (4C), 134.9 (2C), 132.3, 130.8, 130.7 (2C), 128.7 (4C), 128.1, 127.8, 78.9, 64.3, 34.0, 33.9, 33.5, 27.9, 27.6 (3C), 25.0, 24.4, 21.4, 19.9, 15.1; IR (film) cm^−1^: 3355, 2959, 2931, 1472, 1428, 1111; HRMS (ESI, positive) m/z [M+Na]^+^ Calcd. for C_32_H_44_NaO_2_Si: 511.3003, found: 511.2980.

##### Synthesis of diol 4

To a THF (4 mL) solution of **3** (58.4 mg, 1.00 μmol), 1 M TBAF was added in THF (600 μL, 600 μmol) under an argon atmosphere. After being stirred at room temperature for 3 h. The reaction mixture was quenched with saturated aqueous NH_4_Cl and extracted with EtOAc. The organic layer was washed with saturated aqueous NaCl, dried over Na_2_SO_4_, and filtered. After evaporation, the residue was purified by medium-pressure column chromatography on silica gel (*n*-hexane/EtOAc = 94/6 to 50/50) to give diol **4** (25.4 mg, 85%) as a colorless oil. ^1^H NMR (400 MHz, pyridine-d_5_) δ_H_: 5.71–5.45 (m, 4H), 5.03 (brt, *J* = 6.4 Hz, 1H), 3.92 (t, *J* = 6.6 Hz, 2H), 3.33 (dd, *J* = 14.7, 7.7 Hz, 1H), 3.15 (dd, *J* = 14.7, 7.3 Hz, 1H), 3.03 (d, *J* = 7.3 Hz, 2H), 2.66–2.47 (m, 1H), 2.41 (q, *J* = 7.3 Hz, 2H), 1.97–1.82 (m, 2H), 2.23 (quintet, *J* = 7.3 Hz, 2H), 1.97–1.82 (m, 1H), 1.85 (quintet, *J* = 6.6 Hz, 2H), 0.96 (t, *J* = 7.3 Hz, 3H); ^13^C NMR (100 MHz, pyridine-d_5_) δ_C_: 138.8, 137.4, 132.3, 131.3, 127.8 (2C), 78.9, 62.0, 34.2, 33.9, 33.9, 27.9, 25.0, 24.8, 21.4, 15.0; IR (neat) cm^−1^: 3336, 3009, 2932, 1454, 1039; HRMS (ESI, positive) *m/z* [M+Na]^+^ Calcd. for C_16_H_26_NaO_2_: 273.1825, found: 273.1819.

##### Synthesis of Δ^4^-dinor-*iso*-OPDA

To a solution of **4** (17.1 mg, 68.3 μmol) in CH_2_Cl_2_/pH 7.0 phosphate buffer (500 μL, 1:1, v/v) at 0°C were sequentially added AZADOL^62^ (1.6 mg, 10.4 μmol) and PhI(OAc)_2_ (111 mg, 343 μmol). The resultant solution was stirred at room temperature for 3 h. The reaction was quenched with saturated aqueous Na_2_S_2_O_3_ solution at 0°C and extracted with EtOAc. The organic layer was washed with saturated aqueous NaCl, dried over Na_2_SO_4_, and filtered. After evaporation, the residue was purified by medium-pressure column chromatography on silica gel (*n*-hexane/EtOAc/AcOH = 84/16/0.1 to 99.9/0.1 EtOAc/AcOH) to give Δ^4^-dinor-*iso*-OPDA (12.3 mg, 69%) as a yellow oil. ^1^H NMR (400 MHz, benezene-d_6_) δ_H_: 5.39–5.48 (m, 2H), 5.23–5.30 (m, 1H), 5.12–5.19 (m, 1H), 3.05 (d, *J* = 5.5 Hz, 2H), 2.85 (d, *J* = 7.3 Hz, 2H), 2.15–2.24 (m, 2H), 2.17 (q, *J* = 6.8 Hz, 2H), 2.10 (t, *J* = 6.8 Hz, 2H), 2.00–2.29 (m, 2H), 1.85 (t, *J* = 4.4 Hz, 2H), 0.99 (t, *J* = 7.3 Hz, 3H); ^13^C NMR (100 MHz, benezene-d_6_) δ_C_: 207.9, 178.4, 170.4, 139.8, 132.9, 130.6, 126.3, 126.2, 34.5, 34.0, 29.8, 29.4, 23.1, 22.1, 21.4, 14.8; IR (neat) cm^−1^: 3011, 2963, 2923, 1731, 1699, 1629; HRMS (ESI, negative) *m/z* [M-H]^-^ Calcd. for C_16_H_21_O_3_: 261.1496, found: 261.1488.

##### Synthesis of diol 6

To a solution of **5**^63^ (51.8 mg, 0.111 mmol) and imidazole (30.4 mg, 0.446 mmol) in DMF (0.90 mL) was added TESCl (56 μL, 0.334 mmol). The mixture was stirred for 6 h, and the reaction was added MeOH (1.0 mL) and H_2_O (10 mL). The mixture was extracted with *n*-hexane. The combined organic layers were dried over Na_2_SO_4_ and concentrated under reduced pressure. The residue was purified by medium-pressure column chromatography on silica gel (*n*-hexane to *n*-hexane/EtOAc = 98/2) to give di-TES-protected intermediate S1 (74.5 mg, slightly impure) as colorless oil and a portion of the product (52.5 mg) was used for the following reaction without further purification.

To a solution of DMSO (59 μL, 757 μmol) in CH_2_Cl_2_ (2.0 mL) was added oxalyl chloride (32 μL, 379 μmol) at −78°C under an argon atmosphere. After the reaction mixture was stirred at −78°C for 15 min, a solution of S1 (52.5 mg, ca. 75.7 μmol) in CH_2_Cl_2_ (3.0 mL) was slowly added. After the reaction mixture was stirred at −65°C for 40 min, Et_3_N (115 μL, 833 μmol) was slowly added. The mixture was gradually warmed to room temperature for 30 min with stirring. The reaction mixture was quenched with saturated aqueous NH_4_Cl. The mixture was extracted with *n*-hexane. The organic layer was washed with saturated aqueous NaCl, dried over Na_2_SO_4_, and filtered. The reaction mixture was concentrated under reduced pressure to afford an aldehyde intermediate, which was used for the following reaction without further purification. To a suspension of [Ph_3_PCH_2_CD_2_CD_3_]^+^Br^−^^64^ (59.1 mg, 151 μmol) in THF (1.5 mL) was added NaHMDS (1 M in THF, 151 μL, 151 μmol) at 0°C. The mixture was stirred at 0°C for 40 min and cooled to −78°C. To this solution was added a solution of the crude aldehyde in THF (2 mL). The reaction mixture was gradually warmed to room temperature for 1 h. Then, the reaction was quenched with saturated aqueous NH_4_Cl and extracted with *n*-hexane. The combined organic layers were washed with saturated aqueous NaCl and dried over Na_2_SO_4_. After evaporation, the residue was purified by a silica gel short pass to give S2 (26 mg, mixture) as pale yellow oil. The crude product was used for the following reaction without further purification. To a solution of S2 (26 mg, mixture) in THF (2 mL) was added 1 M TBAF in THF (215 μL, 215 μmol). After being stirred at reflux temperature for 1 h, the solvent was removed under reduced pressure. The residue was purified by medium-pressure column chromatography on silica gel (*n*-hexane/EtOAc = 93/7 to 60/40) to give **6** (4.7 mg, 24% in 4 steps from **5**) as a colorless oil. ^1^H NMR (400 MHz, CDCl_3_) δ_H_: 6.14 (dd, *J* = 5.5, 2.8 Hz, 1H), 5.97 (ddd, *J* = 5.5, 2.8, 1.4 Hz, 1H), 5.51–5.37 (m, 4H), 4.51 (dd, *J* = 5.5, 2.8 Hz, 1H), 3.62 (t, *J* = 6.0 Hz, 2H), 2.67–2.58 (m, 1H), 2.40–1.93 (m, 7H), 1.74–1.47 (m, 2H); ^13^C NMR (100 MHz, CDCl_3_) δ_C_: 141.3, 132.5, 132.3, 129.9, 128.9, 127.8, 76.5, 61.6, 46.0, 45.9, 32.2, 30.5, 23.4, 23.1; HRMS (ESI, positive) m/z [M+Na]^+^ Calcd. for C_16_H_21_D_5_NaO_2_: 278.2139, Found: 278.2133.

##### Synthesis of Δ^4^-dinor-*cis*-OPDA-d_5_

To a solution of **6** (3.0 mg, 11.7 μmol) in acetone (1.0 mL) was added Jones reagent (4.0 M solution) at −20°C until the orange color of the reagent persisted (4 drops). After 30 min of stirring at −10°C, *i*-PrOH was added to quench the remaining reagent. Then, EtOAc/*n*-hexane (1/1, 10 mL) and H_2_O (10 mL) were added, and the water layer was extracted with EtOAc. The combined organic layers were washed with saturated aqueous NaCl, dried over Na_2_SO_4,_ and concentrated under reduced pressure. The residue was purified by medium-pressure column chromatography on silica gel (*n*-hexane/EtOAc/AcOH = 88/12/0.1 to EtOAc/AcOH = 99.9/0.1) to give Δ^4^-dinor-*cis*-OPDA-d_5_ (2.8 mg, 89%) as a colorless oil. [α]_D_^30^ + 138.9 (*c* 0.15, CHCl_3_). ^1^H NMR (400 MHz, CDCl_3_) δ_H_: 7.66 (dd, *J* = 5.8, 2.8 Hz, 1H), 6.20 (d, *J* = 5.8 Hz, 1H), 5.55–5.34 (m, 4H), 3.10–3.00 (m, 1H), 2.60–2.26 (m, 7H), 2.23–2.11 (m, 1H), 2.04–1.92 (m, 1H); ^13^C NMR (100 MHz, CDCl_3_) δ_C_: 210.7, 178.3, 166.9, 133.2, 132.7, 129.5, 128.1, 126.8, 49.4, 44.2, 33.6, 28.5, 23.9, 22.5; HRMS (ESI, negative) *m/z* [M-H]^-^ Calcd. for C_16_H_16_D_5_O_3_: 266.1810, Found: 266.1800.

#### UPLC-MS/MS analyses of wound-induced Δ^4^-dn-OPDAs and *in-planta* isomerization from *cis*-to *iso*-form

*Marchantia polymorpha* accession Takaragaike-1 (Tak-1; male) was used in this study as the wild-type (WT). *M. polymorpha* plants were grown on half Gamborg’s B5 1% agar plates for two weeks under continuous light at 22°C. Mechanical wounding was performed with tweezers on 2-week-old plants as previously describe*d.*^31^ Plants were transferred to 500 μL of liquid half Gamborg’s B5 media containing mock or ca. 1 μL Δ^4^-dn-*cis*-OPDA-*d*_*5*_ and incubated under normal light and temperature conditions for 30 min and 2 h. Plants were washed with dist. H_2_O to remove the medium. Following the above treatments, each plant was collected and weighed, then was flash frozen in liquid nitrogen and ground using a bead crusher with temporal cooling in liquid nitrogen. Extraction was performed: each plant powder (*n* = 1) was added 1 mL of 90% aq. MeOH with 1% formic acid and incubated for 1 h vortex mixing. Following centrifugation at 20,000 g for 10 min, the supernatant was collected, and added 500 μL of 90% aq. MeOH with 1% formic acid and incubated for 20 min vortex mixing. Following centrifugation at 20,000 g, for 10 min, the supernatant was colected. The eluent solution was evaporated to dryness at 30°C under reduced pressure and freeze-drying. Samples were then re-suspended in 100 μL of 100% MeOH.

*P. patens* plants were grown on BCD solid medium for four weeks under continuous light at 25°C. *P. patens* wounding was performed by scraping off about 900 mg fresh gametophores from agar plate cultures and squashed with a plastic pestle. Subsequently, the sample was incubated under light for 120 min. The wounded sample was then frozen in liquid nitrogen. Extraction was performed: each plant powder (*n* = 1) was added 9 mL of 90% aq. MeOH with 1% formic acid and incubated for 1 h vortex mixing. Following centrifugation at 15,000 g for 10 min, the supernatant was transferred to another tube, added 7.5 mL of 90% aq. MeOH with 1% formic acid and incubated for 20 min vortex mixing, and additional centrifugation at 15,000 g for 10 min was performed. The eluent solution was evaporated to dryness at 30°C under reduced pressure and freeze-drying. Samples were then re-suspended in 100 μL of 100% MeOH.

*A. agrestis* plants were grown on KNOP solid medium for four weeks under continuous light at 22°C. *A. agrestis* wounding was performed with tweezers. Subsequently, the sample was incubated under light for 120 min. The wounded sample was then frozen in liquid nitrogen. Extraction was performed: each plant powder (*n* = 1) was added 15 mL of 90% aq. MeOH with 1% formic acid and incubated for 1 h vortex mixing. Following centrifugation at 15,000 g for 10 min, the supernatant was transferred to another tube, added 7.5 mL of 90% aq. MeOH with 1% formic acid and incubated for 20 min vortex mixing, and additional centrifugation at 15,000 g for 10 min was performed. The eluent solution was evaporated to dryness at 30°C under reduced pressure and freeze-drying. Samples were then re-suspended in 100 μL of 100% MeOH.

10 μL of sample was subjected to Ultra-performance liquid chromatography coupled with triple quad mass spectrometry (UPLC-MS/MS) analysis on an Agilent 1290 Infinity II (Agilent Technologies, USA) associated with an Ultivo Triple Quad LC/MS (Agilent Technologies, USA), equipped with ZORBAX Eclipse Plus C18 column (1.8 μm, 2.1 × 50 mm; Agilent Technologies). UPLC condition was as follows; mobile phases: A, water with 0.1% (v/v) formic acid; B, acetonitrile with 0.1% (v/v) formic acid: the gradient program: 0.0 to 5.0 min, isocratic 15% B; 5.0 to 15.0 min, linear gradient 15 to 45% B; 15.0 to 15.1 min, linear gradient 45 to 100% B; 15.1 to 18.0 min, linear gradient 100 to 10% B; 18.0 to 18.1 min, linear gradient 10 to 15% B; 18.1 to 20.0 min, isocratic 15% B, flow rate: 0.40 mL min^−1^. The mass spectrometry analysis was carried out on a negative mode under the following conditions: the capillary voltage = 3500 V, the nebulizer gas pressure = 1.38 bar, the desolvation gas flow = 13.0 L min^−1^, the gas temperature = 340°C, the Sheath gas flow = 12.0 L min^−1^, the Sheath gas temperature = 380°C. Multiple Reaction Monitoring (MRM) mode, using the following precursors and fragments:

Δ^4^-dn-OPDA-d5: precursor ion 266, fragment ion 168.

Δ^4^-dn-OPDA: precursor ion 261, fragment ion 163.

#### Preparation of GST-MpCOI1 protein

Standard methods for cloning were used, and PCR-amplified DNA fragments were sequenced after cloning into the vectors. The plasmids of GST-fused AtCOI or AtASK1 (pFB-GTE-COI1 and pFB-HTB-ASK1) were obtained from Addgene (https://www.addgene.org/). The full-length CDS of MpCOI1 was obtained from pMpGWB311-35S::MpCOI1-flag and cloned into pFB-GTE-COI1 to prepare the plasmid of GST-fused MpCOI1(GST-MpCOI1). The heterologous expression system of GST-MpCOI1 was then designed by Bac-to-Bac system according to the manufacturer’s procedure using DH10Bac competent cells and bacmid transfection using ViaFect reagent.

#### Pull-down experiments using full-length MBP-JAZ proteins

Pulldown assays were performed based on the previously described method^39^ using recombinant GST-MpCOI1 proteins co-expressed with AtASK1 and recombinant MBP-MpJAZ protein. Experiments were performed using the previous method.^65^ Anti-GST-HRP (RPN1236, GE Healthcare), anti-MBP antibody (016–24141, Wako), and anti-rat-IgG-HRP (sc-2032, Santa Cruz Biotechnology) were used for detection.

#### *In silico* docking simulation of Δ^4^-dn-*iso*/*cis*-OPDA with a model structure of MpCOI1-MpJAZ

The crystallographic structure of the AtCOI1-JA-Ile-AtJAZ complex (PDB ID: 3OGL) was used to obtain the models of MpCOI1 and MpJAZ degron peptide. The model structure of MpCOI1 was constructed by ColabFold.^66^ The homology model of the MpJAZ degron peptide was constructed by the molecular operating environment (MOE) from the Chemical Computing Group. The obtained model structures were merged on MOE2021.09 to form the MpCOI1/MpJAZ co-receptor model. A docking simulation of the ligand was then conducted against the MpCOI1/MpJAZ co-receptor model by MOE2021.09.

#### Growth inhibition assays

Gemmae were placed in sterilized 0.5 Gamborg’s B5 medium with 1% agar in long-day conditions (50–60 μmol m^−2^ s^−1^, 16-h) containing different concentrations of dn-*iso*-OPDA and Δ4 -dn-*iso*-OPDA at 21°C. Images of two-week-old plants were acquired using NIKON D1-x digital camera. ImageJ software was used to measure the area of each plant. 12 to 18 plants were analyzed in each experiment. The assay was repeated twice with similar results. Statistical significance was determined by one-way ANOVA with post-hoc Tukey’s honestly significant difference (HSD) test (*p* < 0.05) (www.statskingdom.com/).

#### RT-qPCR analysis of JA marker genes in *Marchantia polymorpha*

Two-week-old Marchantia plants (*N* = 3 to 5) grown in half Gamborg’s B5 0.5% agar medium were transferred to half Gamborg’s B5 liquid medium with different concentrations of dn-*iso*-OPDA and Δ4-dn-*iso*-OPDA and soaked for 2 h. Samples were frozen in liquid nitrogen, and RNA was extracted using FavorPrep Plant Total RNA Purification Mini Kit. cDNA was obtained using AppliedBiosystems MultiScribe Reverse Transcriptase. qPCRs were operated in the Quantstudio 5 Real-Time PCR System from Applied Biosystems^TM,^ and data results were obtained by the Δct method. The assay was repeated three times with similar results.

### Quantification and statistical analysis

#### Statistical analyses

Statistical significance was determined by one-way ANOVA with post-hoc Tukey’s honestly significant difference (HSD) test (*p* < 0.05) (www.statskingdom.com/).

## Supplemental information

Document S1. Figures S1–S10, Table S1, and Datas S1–S7
