# Supplementary material for: Δ4-dn-iso-OPDA, a bioactive plant hormone of Marchantia polymorpha
Source: iScience. 2024 Jun 6;27(7):110191. doi: 10.1016/j.isci.2024.110191 (PMC11225365; doi:10.1016/j.isci.2024.110191)
Supplement: Document S1. Figures S1–S10, Table S1, and Datas S1–S7 [file mmc1.pdf]

iScience, Volume *27*

Supplemental information

Δ^4-dn-*iso*-OPDA, a bioactive plant hormone of *Marchantia polymorpha*

Takuya Kaji, Yuho Nishizato, Hidenori Yoshimatsu, Akiyoshi Yoda, Wenting Liang, Andrea Chini, Gemma Fernández-Barbero, Kei Nozawa, Junko Kyozuka, Roberto Solano, and Minoru Ueda

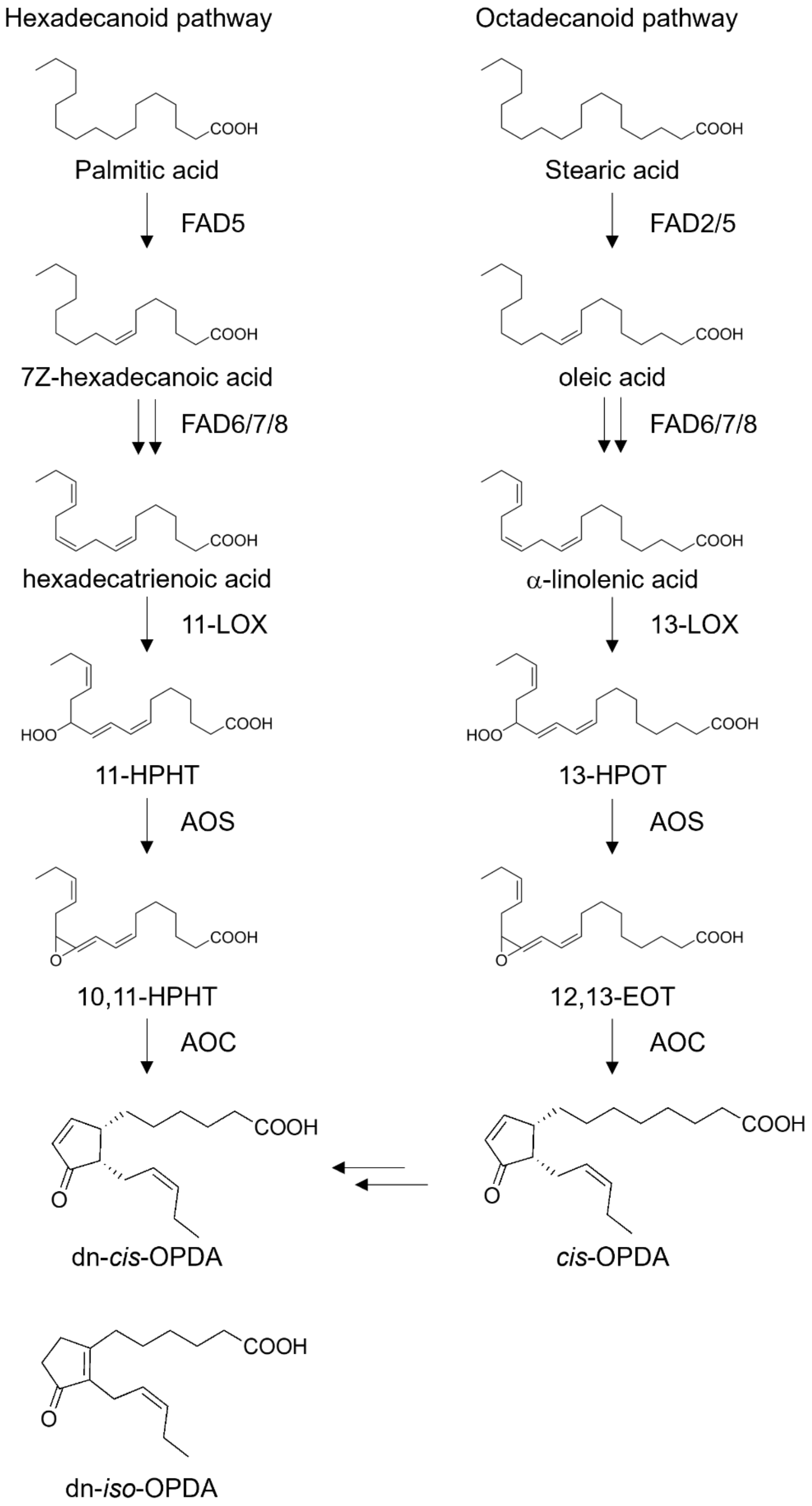

Figure S1. Biosynthetic pathway of dn-OPDAs; hexadecanoid and octadecanoid pathways, related to Figure 1.

A.

no.	^{1}H	^{13}C
1	3.82, t (6.5)	64.28, CH_2
2	1.75, quintet (6.5)	33.44, CH_2
3	2.32, q (6.5)	24.38, CH_2
4	5.44-5.56, m	130.77, CH
5	5.44-5.56, m	128.09, CH
6	3.02, d (6.5)	27.88, CH_2
7	-	138.86 or 137.30, C
8	2.44-2.51, m	33.91, CH_2
8'	2.20-2.37, m	33.91, CH_2
9	2.20-2.37, m	33.91, CH_2
9'	1.92-2.02, m	33.91, CH_2
10	5.03-5.18, m	78.89, CH
11	-	138.86 or 137.30, C
12	3.44, dd (14.7, 7.6)	24.94, CH_2
12'	3.15, dd (14.7, 7.6)	24.94, CH_2
13	5.61-5.77, m	127.82, CH
14	5.44-5.56, m	132.34, CH
15	2.24, quintet (7.5)	21.36, CH_2
16	0.98, t (7.5)	14.99, CH_3

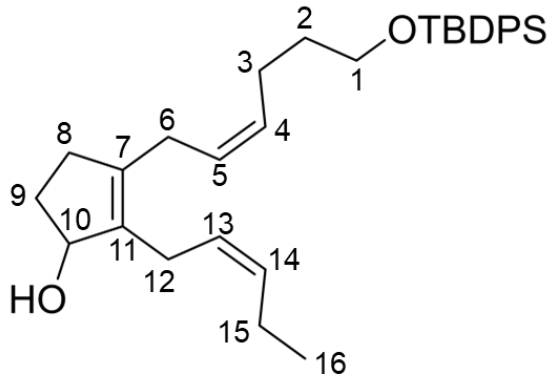

Figure S2. Structure determination of Compound 3, related to Figure 2. (A) ^{1}HNMR and ^{13}C NMR Spectroscopic Data for Compound **3** in pyridine-d$_5$ (δ in ppm and J in Hz).

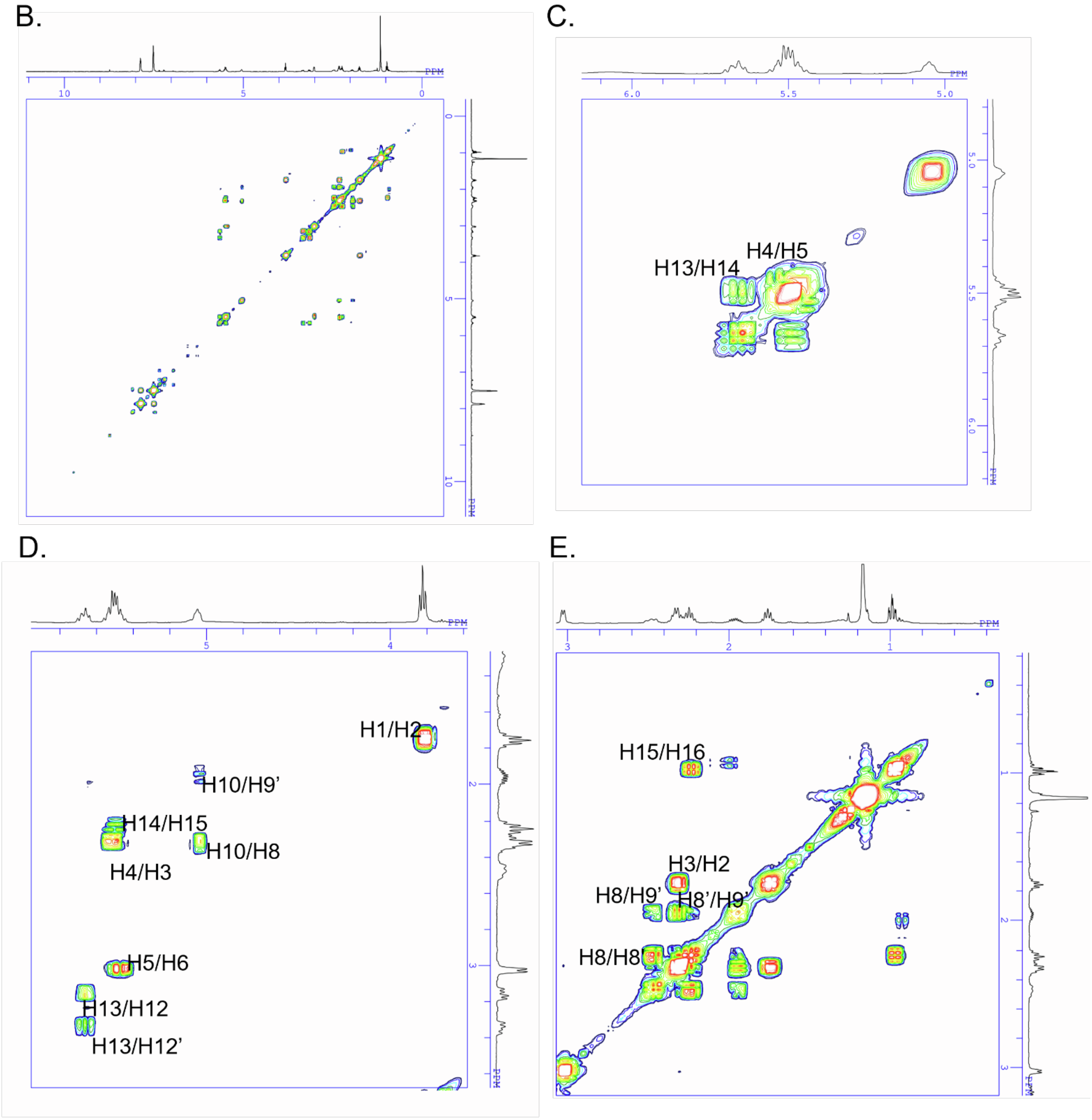

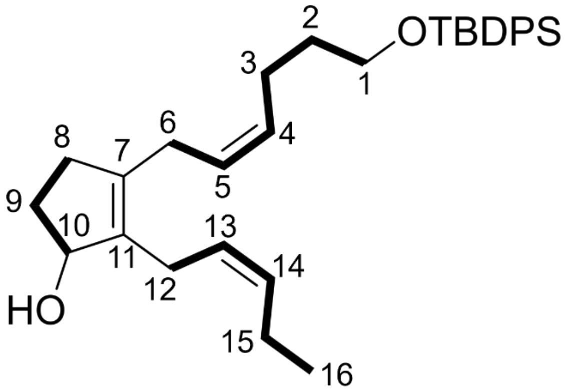

Figure S2 (continued). (B-E) Whole and expanded views of COSY of **3**.

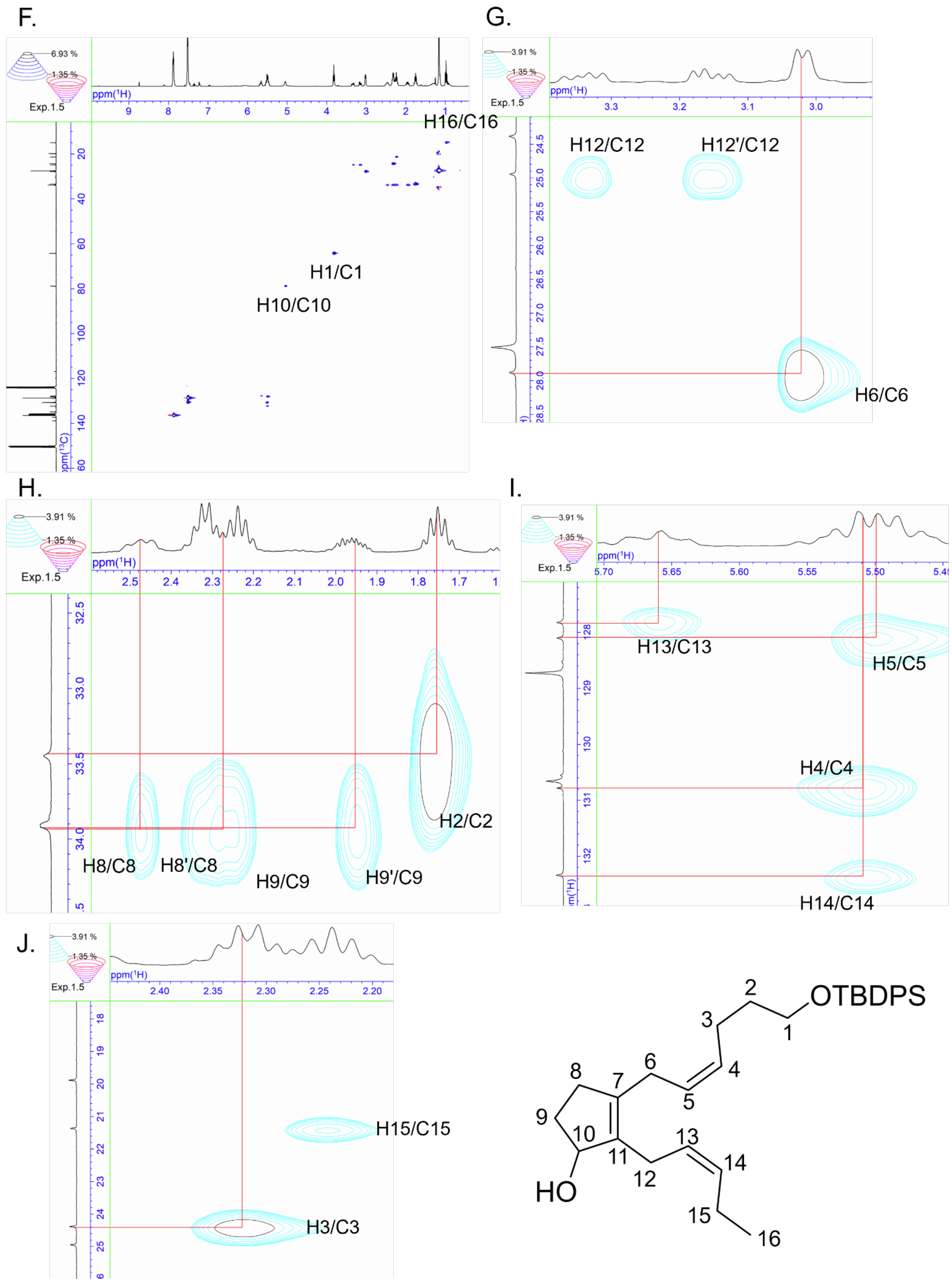

Figure S2 (continued). (F-J) Whole and expanded views of HSQC of **3**.

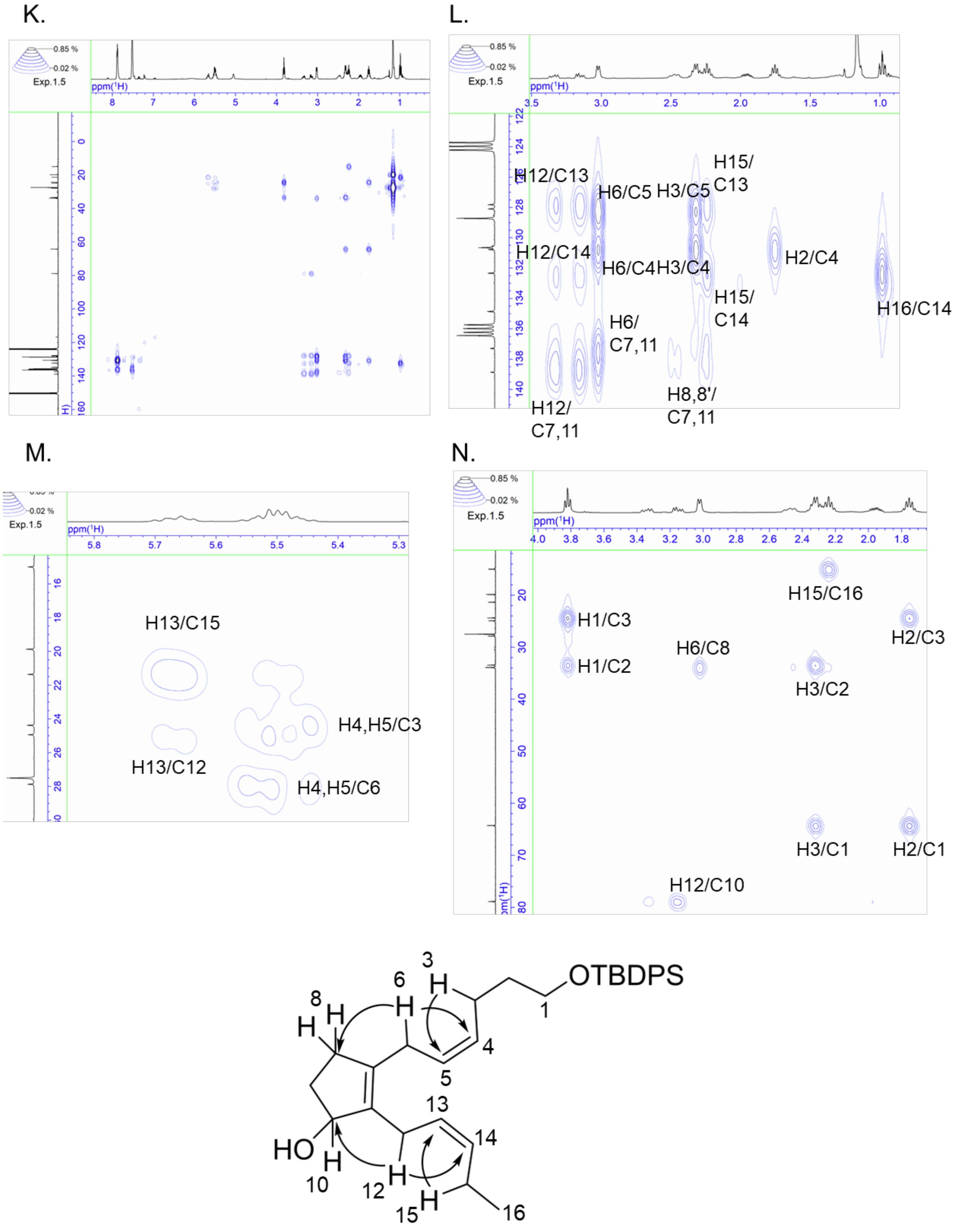

Figure S2 (continued). (K-N) Whole and expanded views of HMBC of **3**.

O.

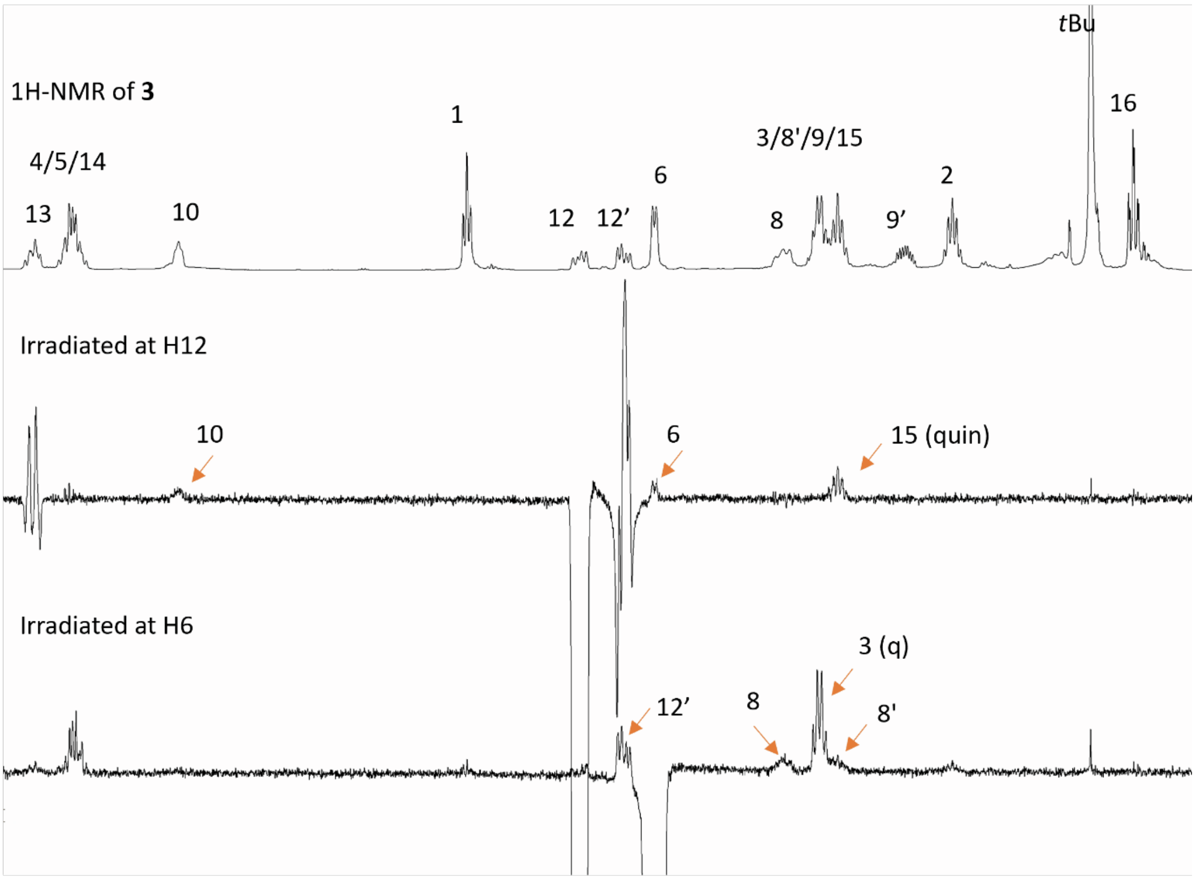

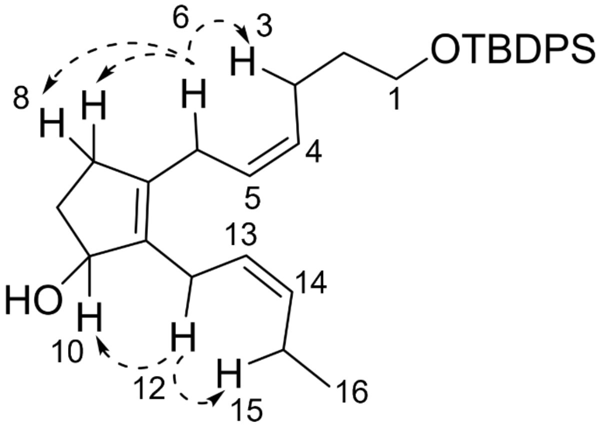

Figure S2 (continued). O. Dif. NOE measurement of **3** at H6 and H12 protons.

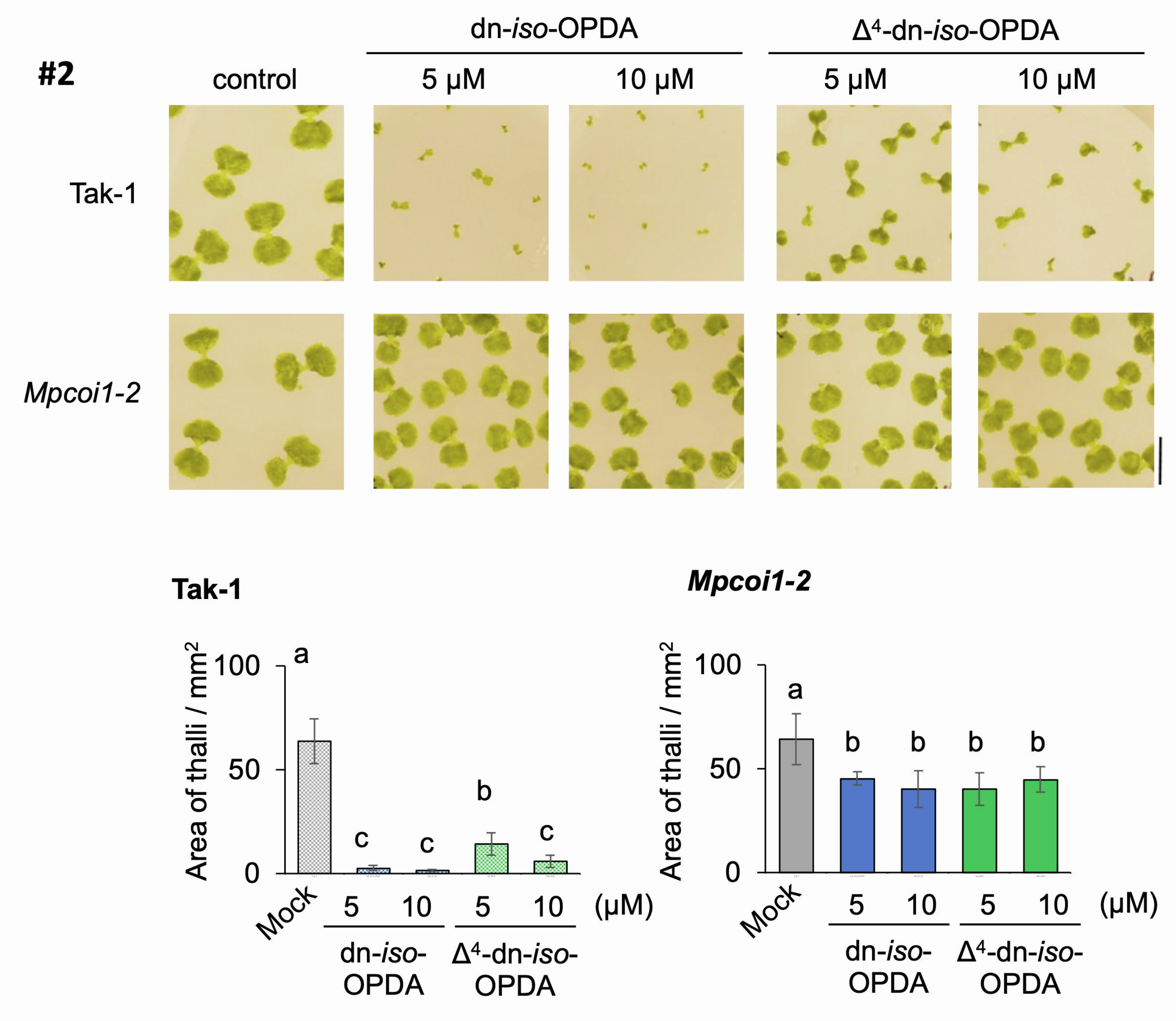

Figure S3. Duplicate of the growth inhibition assay. Effect of various concentrations (5, 10 μM) of dn-*iso*-OPDA and Δ^4-dn-*iso*-OPDA on the growth of WT (Tak-1), *Mpcoi1-2*, related to Figure 4. Experiments were repeated twice with similar results. Scale bar, 1 cm. Different letters indicate statistically significant differences between the percentage of growth in mock, dn-*iso*-OPDA, and Δ^4-dn-*iso*-OPDA treated plants at the indicated concentration (Tukey's HSD [honestly significant difference] ANOVA test; $\alpha = 0.05$, n = 12-18).

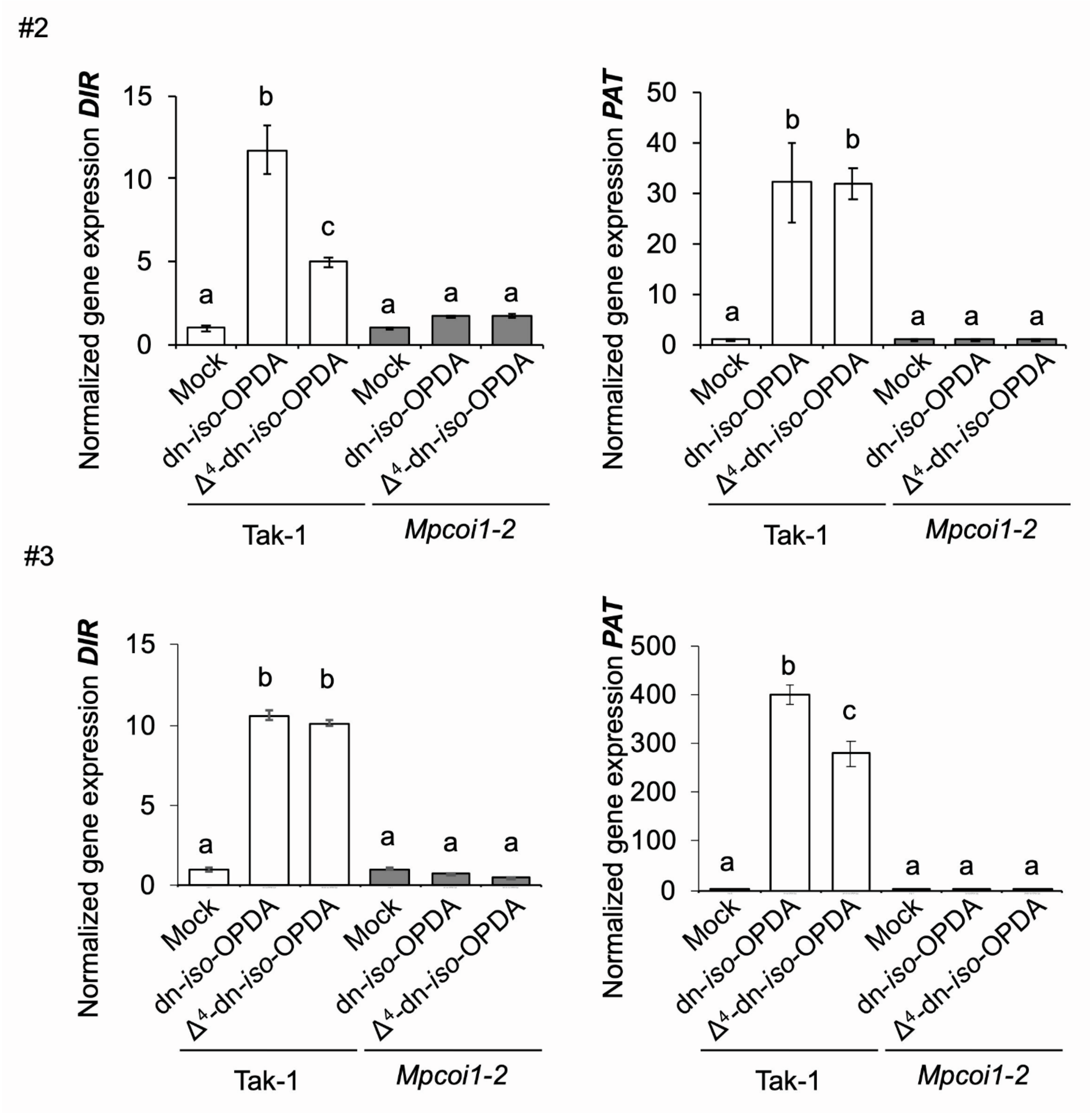

Figure S4. Triplicate of the gene expression analysis of JA-marker genes of *M. polymorpha* against Tak-1 and *Mpcoi1-2*, related to Figure 4. Gene expression analysis by RT-PCR in WT (Tak-1) and *Mpcoi1-2* with or without compounds (dn-*iso*-OPDA, Δ^4-dn-*iso*-OPDA, 5 μM) treatment for 2 hours. Error bars represent SD (n = 3). One-way analysis of variance (ANOVA) with post-hoc Tukey's honestly significant difference (HSD) test ($P < 0.05$) analyses define the significant differences in gene expression.

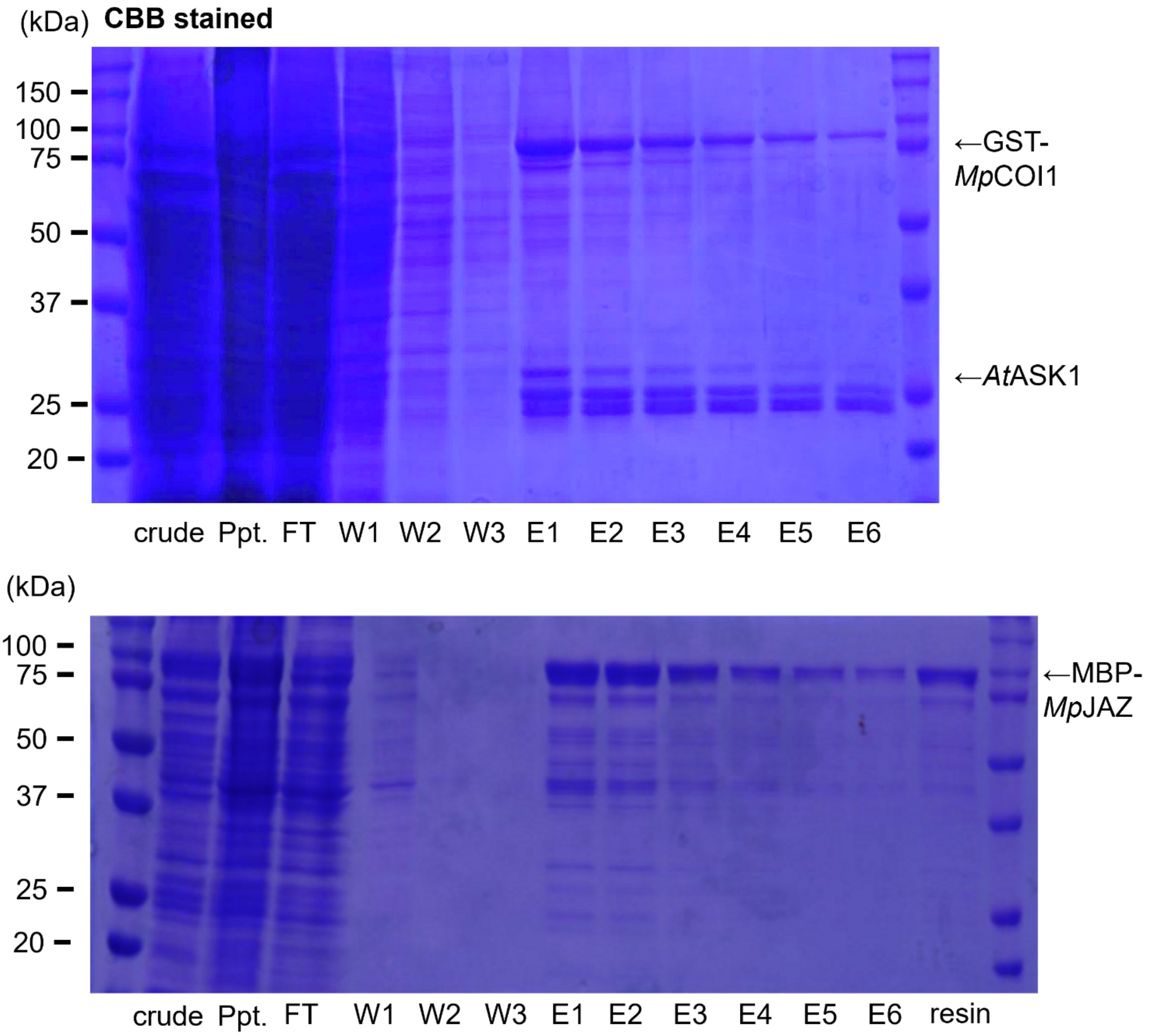

Figure S5 Co-expression of GST-*Mp*COI1 and *At*ASK1 in Sf9 and expression of MBP-*Mp*JAZ in *E. coli*, related to Figure 5.

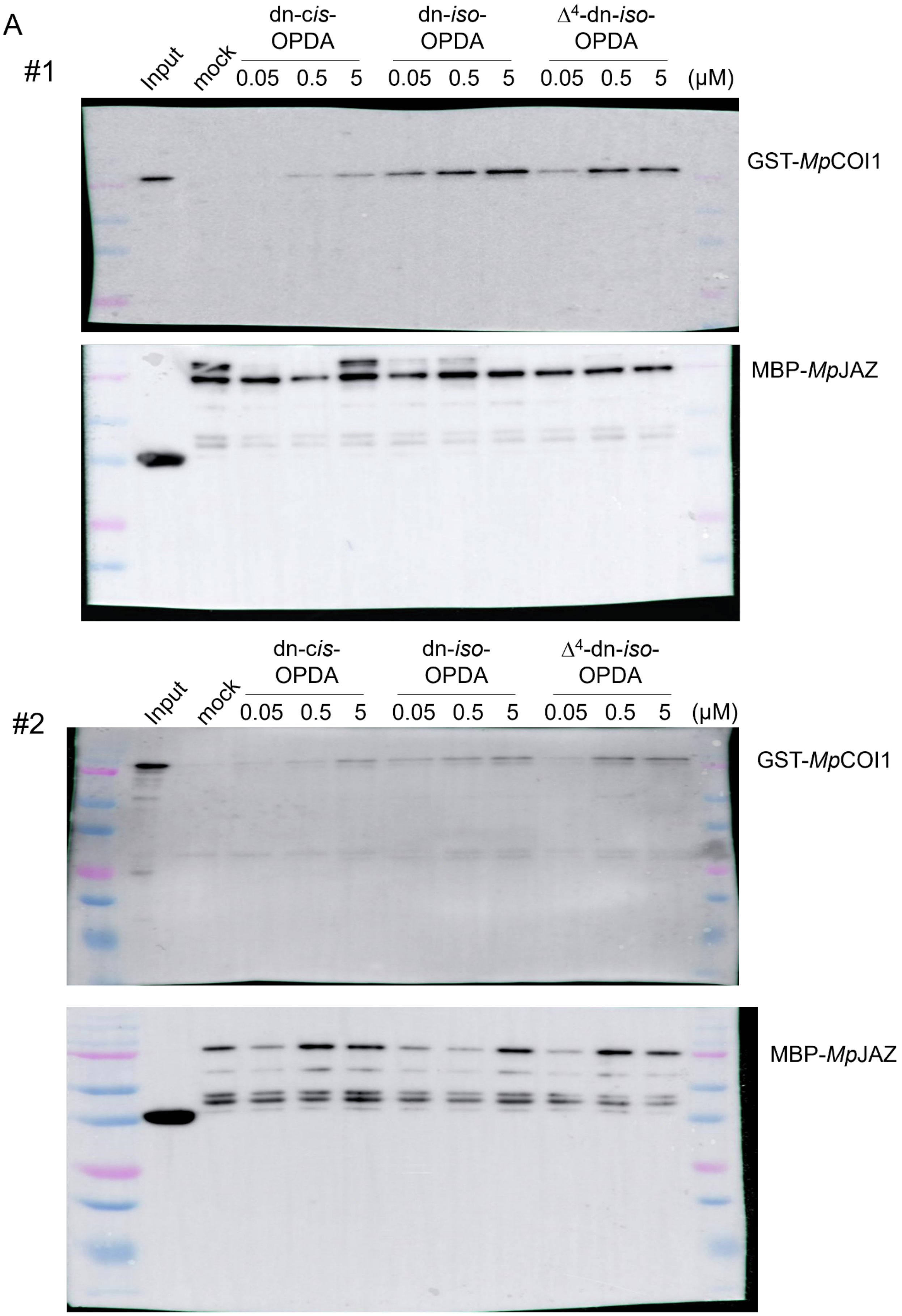
A
#1
Input
mock
dn-cis-OPDA
dn-iso-OPDA
Δ4-dn-iso-OPDA
0.05 0.5 5 0.05 0.5 5 0.05 0.5 5 (μM)
GST-MpCOI1
MBP-MpJAZ
#2
Input
mock
dn-cis-OPDA
dn-iso-OPDA
Δ4-dn-iso-OPDA
0.05 0.5 5 0.05 0.5 5 0.05 0.5 5 (μM)
GST-MpCOI1
MBP-MpJAZ

A

#3

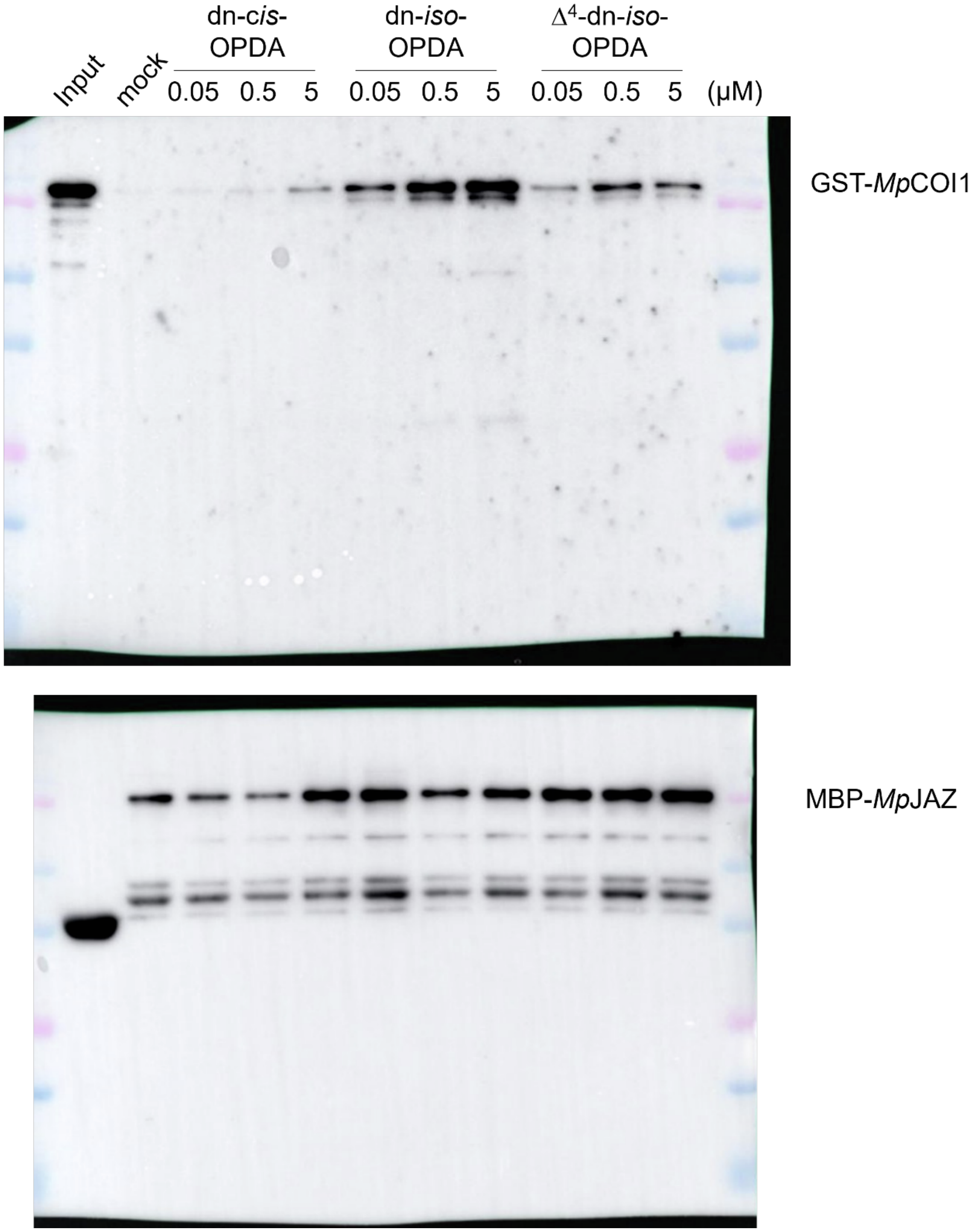

Figure S6 Triplicate results of Pull-down assays, related to Figure 5. (A) Triplicate results of Pull-down assay using GST-*Mp*COI1 and MBP-*Mp*JAZ with dn-*cis*-OPDA, dn-*iso*-OPDA, Δ^4-dn-*cis*-OPDA, Δ^4-dn-*iso*-OPDA, or mock treatment in the pull-down buffer (the indicated concentrations). (Upper) Immunoblot with anti-GST-HRP antibody. (Lower) Immunoblot with anti-MBP and anti-IgG-HRP antibodies.

B

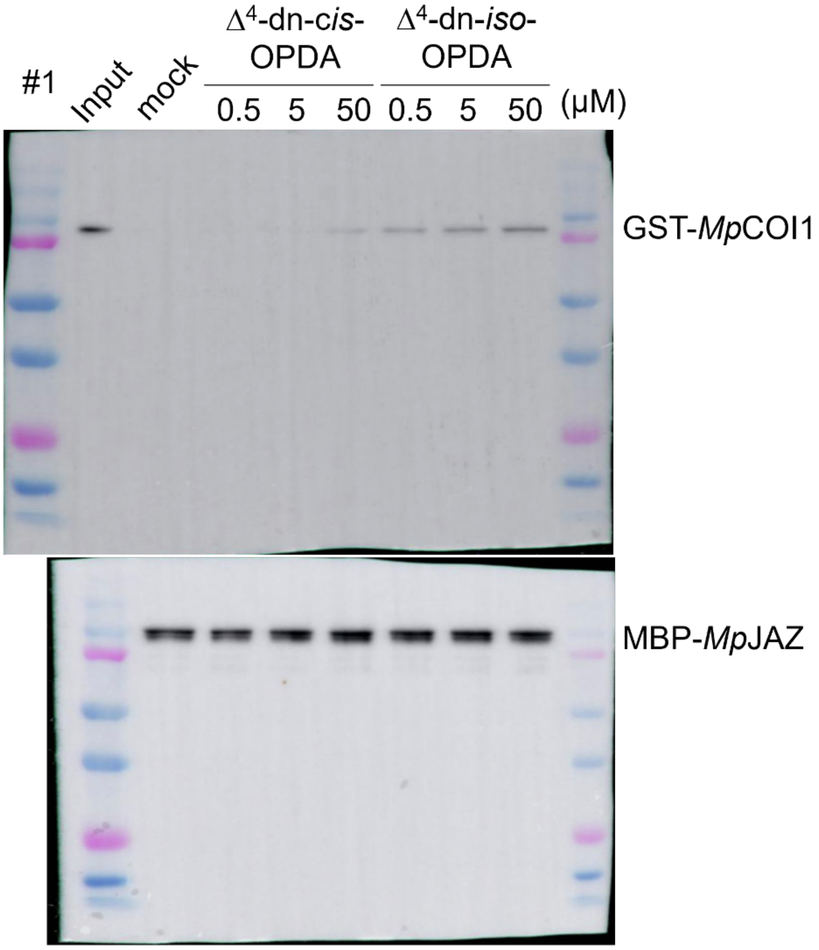
#1
Input
mock
Δ4-dn-cis-OPDA
Δ4-dn-iso-OPDA
0.5
5
50
0.5
5
50
(μM)
GST-MpCOI1
MBP-MpJAZ

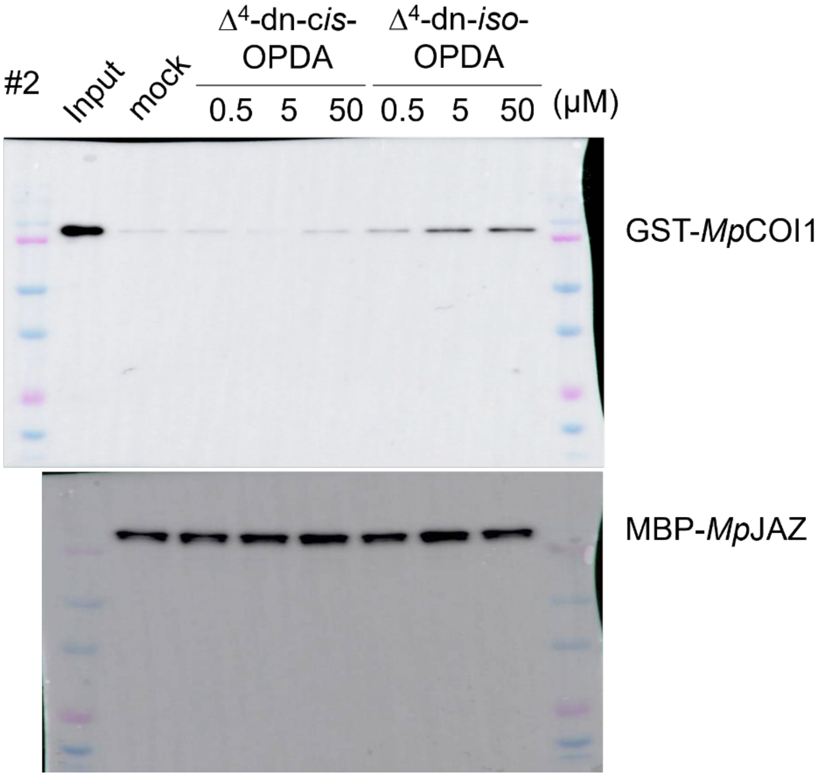
#2
Input
mock
Δ4-dn-cis-OPDA
Δ4-dn-iso-OPDA
0.5
5
50
0.5
5
50
(μM)
GST-MpCOI1
MBP-MpJAZ

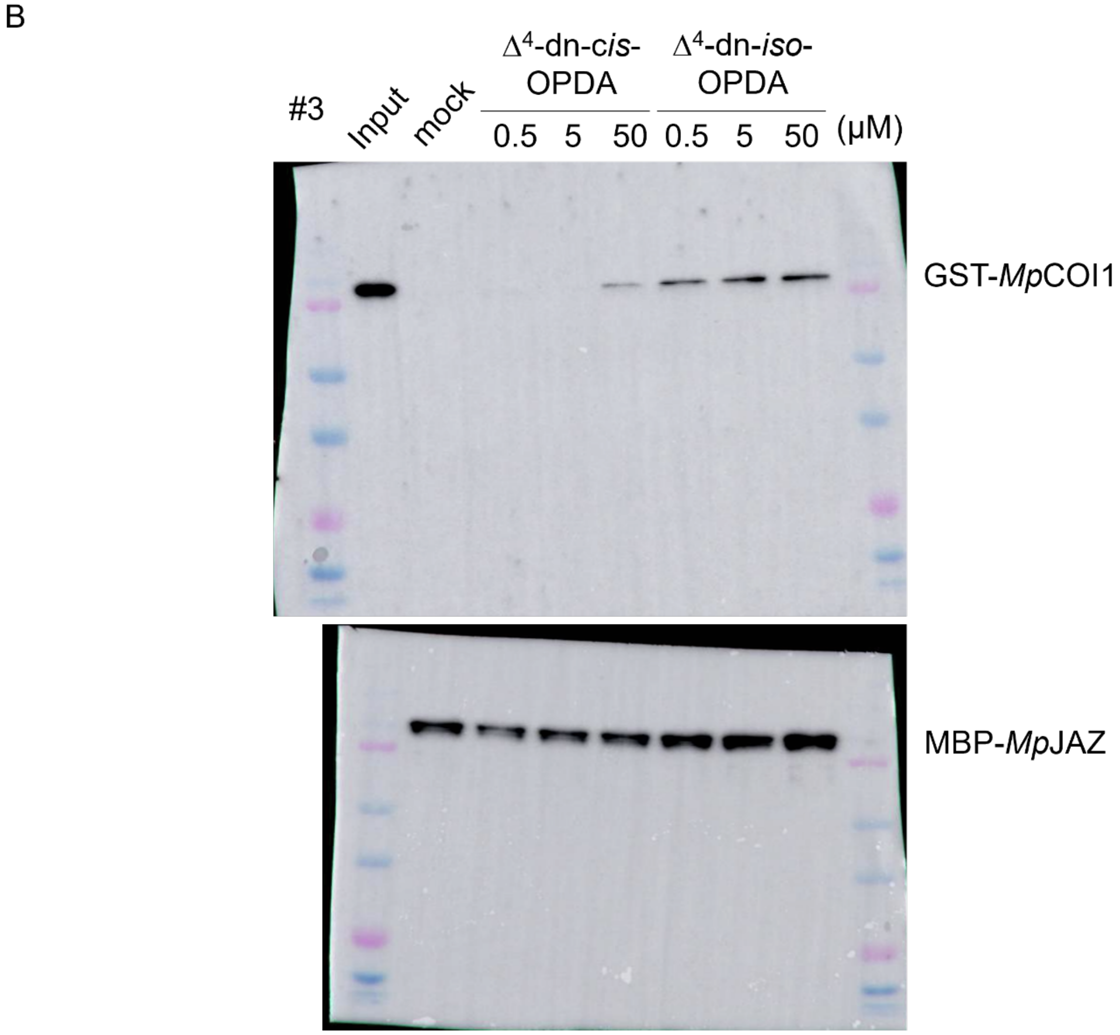

Figure S6 (continued). (B) Triplicate results of Pull-down assay using GST-*Mp*COI1 and MBP-*Mp*JAZ with dn-c*is*-OPDA, Δ^4-dn-c*is*-OPDA, Δ^4-dn-*iso*-OPDA or mock treatment in the pull-down buffer (the indicated concentrations). (Upper) Immunoblot with anti-GST-HRP antibody. (Lower) Immunoblot with anti-MBP and anti-IgG-HRP antibodies.

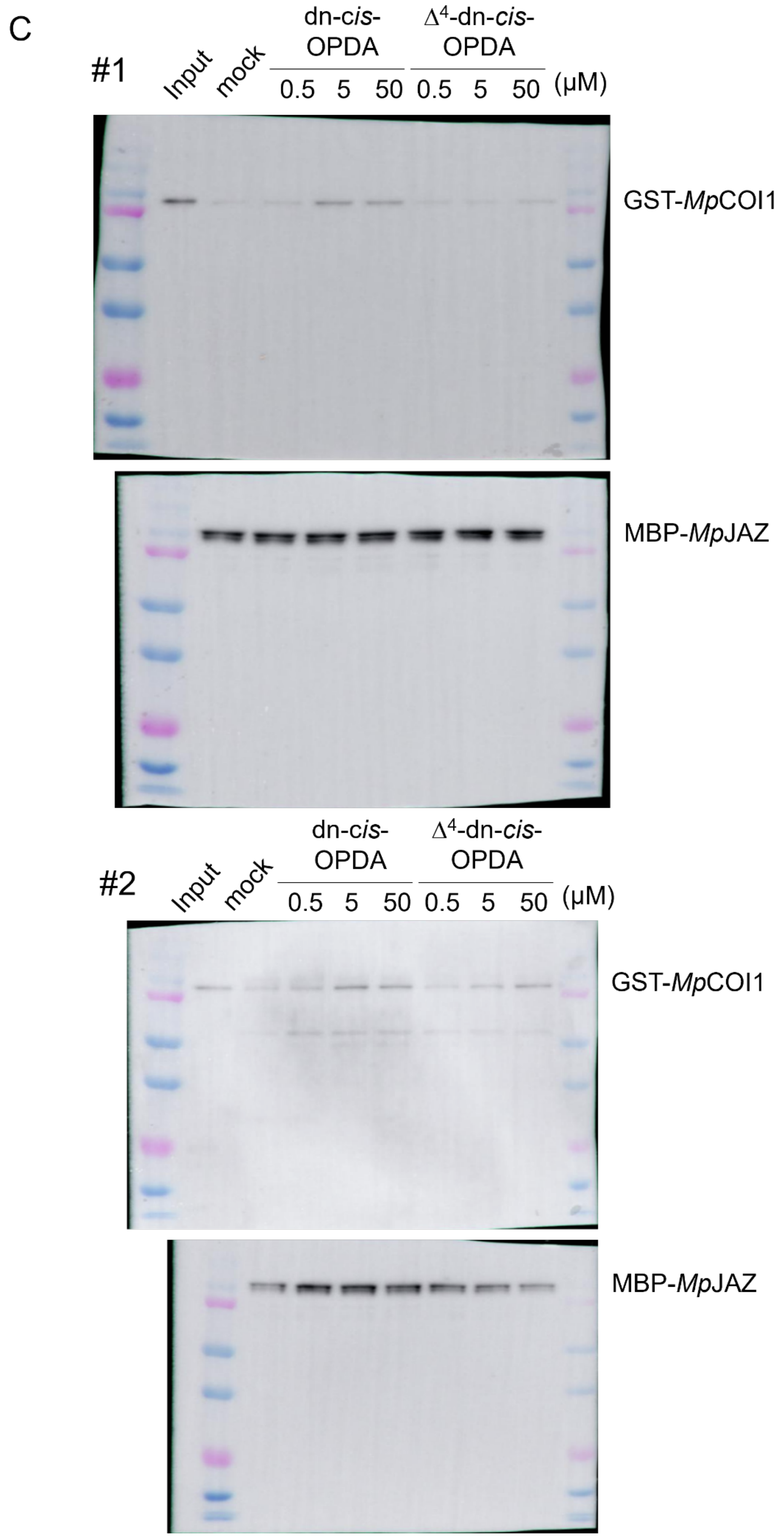
C
#1
Input
mock
dn-cis-OPDA
Δ4-dn-cis-OPDA
0.5 5 50 0.5 5 50 (μM)
GST-MpCOI1
MBP-MpJAZ
#2
Input
mock
dn-cis-OPDA
Δ4-dn-cis-OPDA
0.5 5 50 0.5 5 50 (μM)
GST-MpCOI1
MBP-MpJAZ

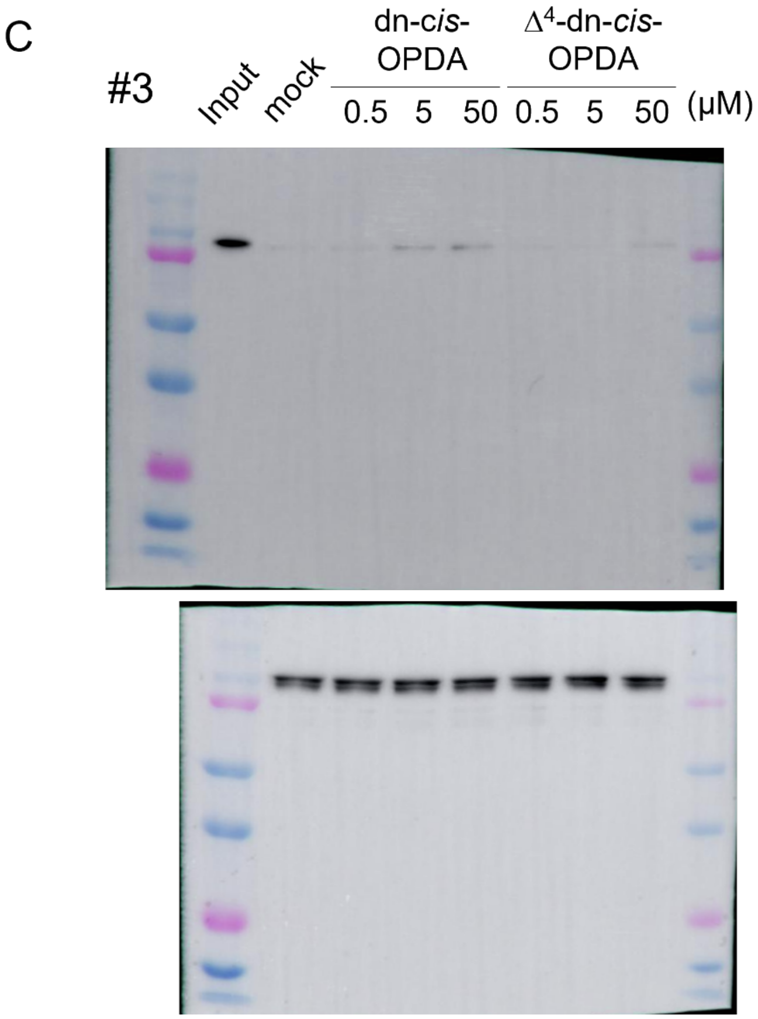

Figure S6 (continued). (C) Triplicate results of Pull-down assay using GST-*Mp*COI1 and MBP-*Mp*JAZ with dn-c*is*-OPDA, dn-c*is*-OPDA, Δ^4-dn-*iso*-OPDA or mock treatment in the pull-down buffer (the indicated concentrations). (Upper) Immunoblot with anti-GST-HRP antibody. (Lower) Immunoblot with anti-MBP and anti-IgG-HRP antibodies.

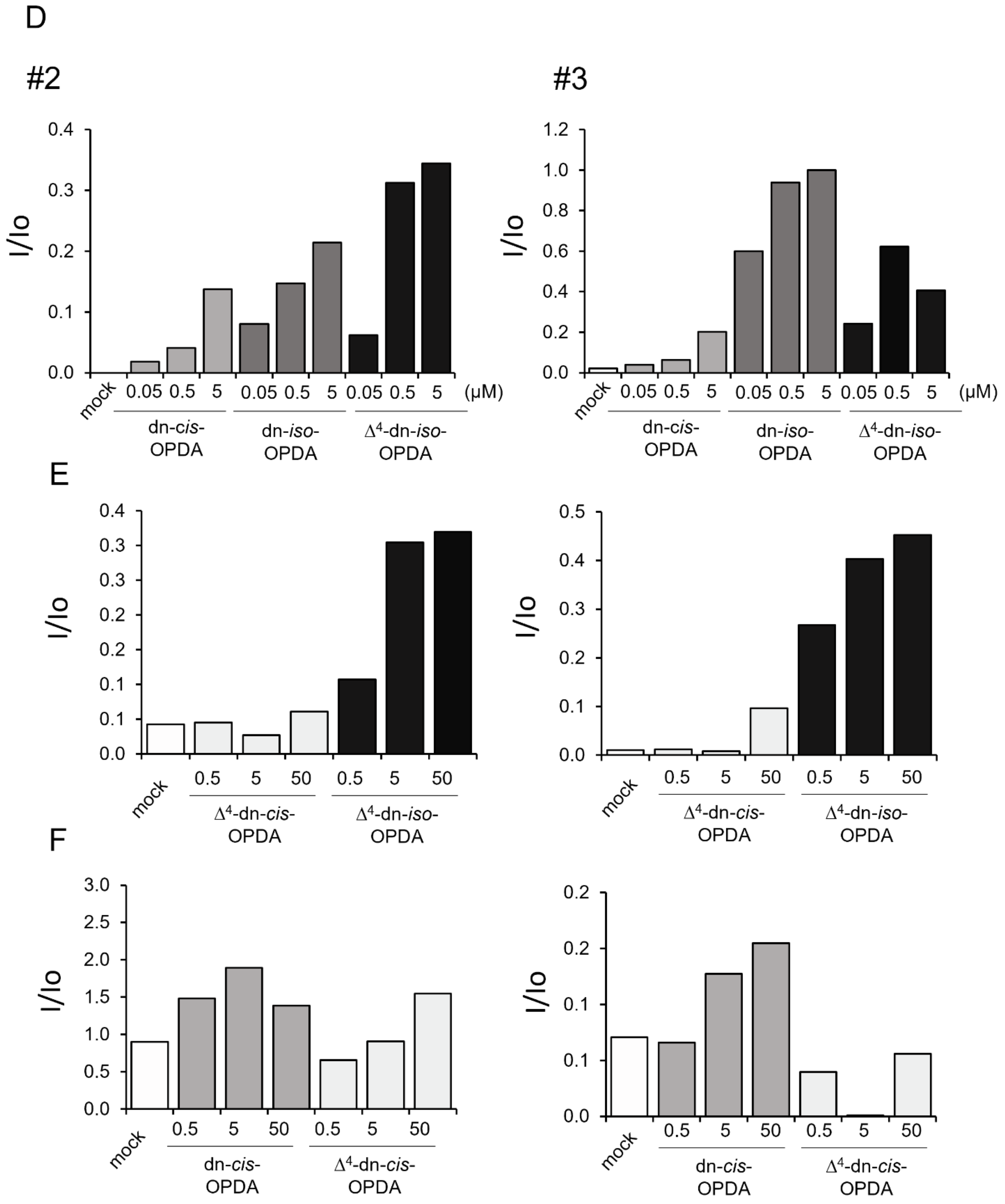

Figure S6 (continued). (**D**)The quantitative data of (**A**). (**E**)The quantitative data of (**B**). (**F**)The quantitative data of (**C**).

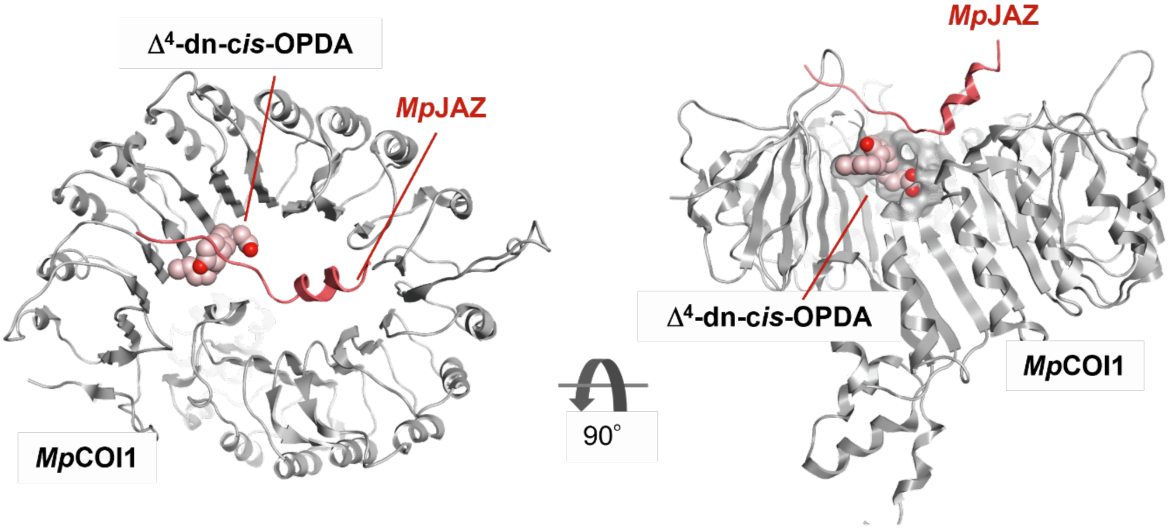

Figure S7 The docking structure of the *Mp*COI1-*Mp*JAZ with Δ^4-dn-*cis*-OPDA, related to Figure 6. **(A)** Top view of the docking structure of the *Mp*COI1-*Mp*JAZ (gray and red ribbons, respectively) and Δ^4-dn-*cis*-OPDA in pink space-fill representation. **(B)** Side view of the docking structure. The sectional view is shown at the ligand binding site for visibility.

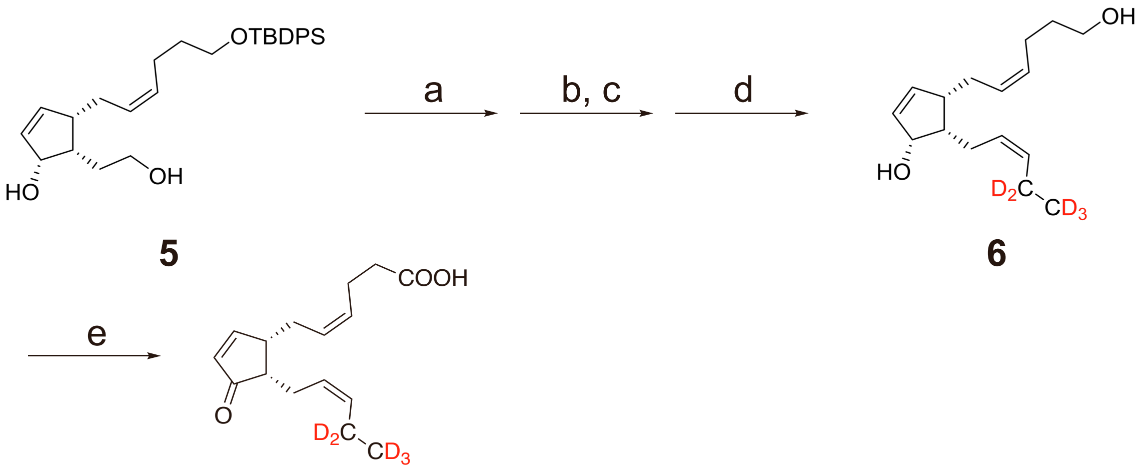

5

6

Δ^4-dn-*cis*-OPDA-*d_5*

Figure S8 Synthesis of Δ^4-dn-*cis*-OPDA-d$_5$, related to Figure 7. (a) TESCl, imidazole, DMF; (b) $(COCl)_2$, DMSO, DCM, -78 ºC; Et_3N, -78 to 0 ºC quant.; (c) $CD_3CD_2(CH_2)_4PPh_3Br$, NaHMDS, THF, –78 ºC; (d) TBAF, THF, 24% in 4 steps from **5**; (e) Jones' reagent, acetone, -10 ºC 89%.

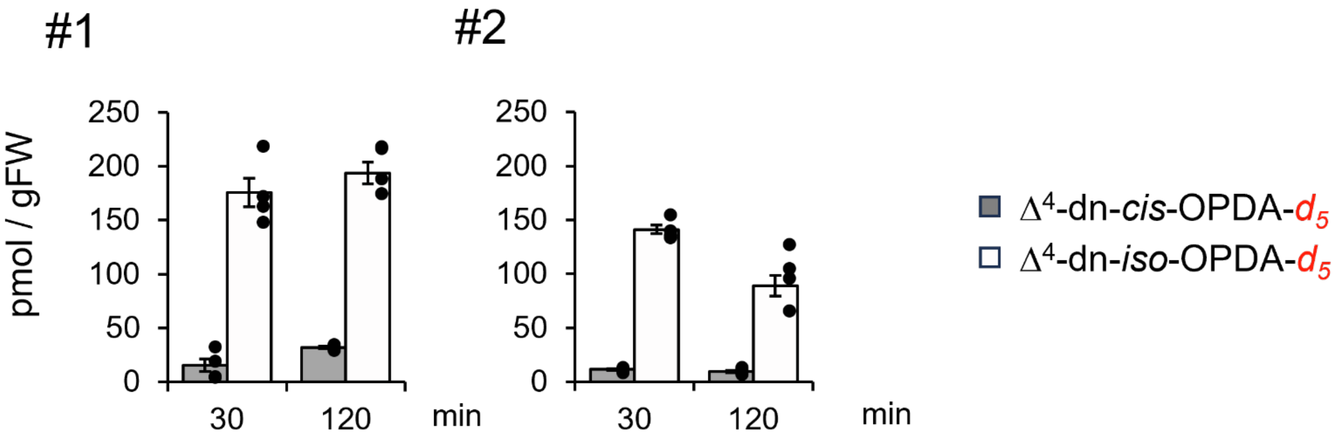

Figure S9. Triplicate results of *cis*-to-*iso* isomerization of Δ^{4}-dn-*cis*-OPDA-d_5 (30 min and 120 min after administration with wounding)**, related to Figure 7.**

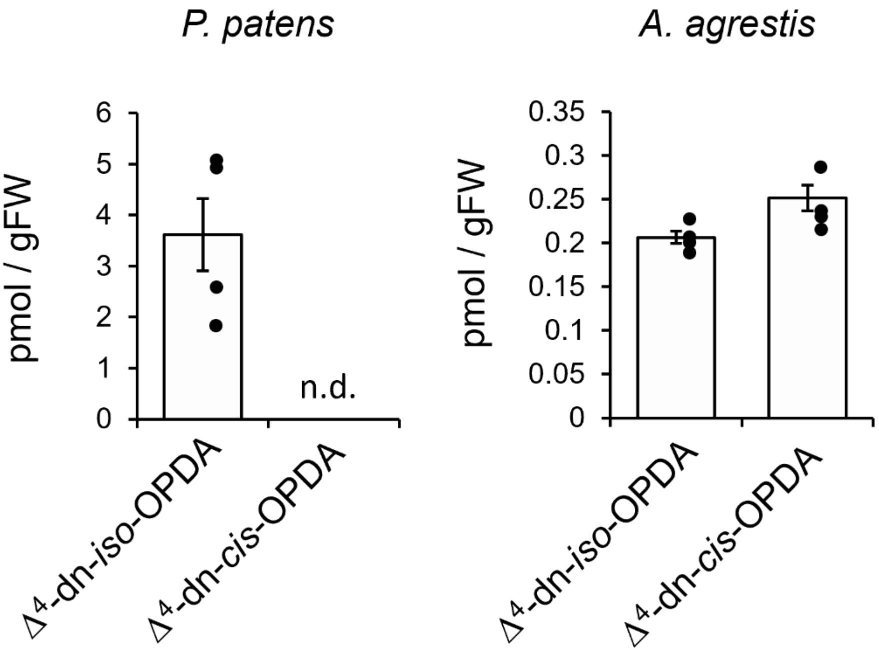

Figure S10. Duplicate results of Δ^4-dn-*cis*/*iso*-OPDA detected in *P.Patens* and *A.angustus*, related to Figure 8.

Table S1. Summarized atom distances of the potential hydrogen bond network in our docking model of Δ^4-dn-*iso/cis*-OPDA with *Mp*COI1-*Mp*JAZ homology model, related to Figure 6. Figures shown in red bold highlight the most deviated distances.

		Δ^4-dn-*iso*-OPDA	Δ^4-dn-*cis*-OPDA
**Donor atom**	**Acceptor atom**	**Distance Å**	**Distance Å**
JAZ Ala306 NH	Δ^4-dn-*iso*-OPDA C=**O**	**3.5**	**4.2**
COI1 Arg489 eNH	Δ^4-dn-*iso*-OPDA C=**O**	**3.5**	**4.0**
COI1 Tyr85 OH	Δ^4-dn-*iso*-OPDA C=**O**OH	1.6	2.0
COI1 Arg348 eNH	Δ^4-dn-*iso*-OPDA C=**O**OH	1.7	1.9
COI1 Arg348 eN'H	Δ^4-dn-*iso*-OPDA C=O**O**H	1.7	2.1
COI1 Arg402 eNH	Δ^4-dn-*iso*-OPDA C=O**O**H	1.8	1.9
COI1 Arg489 eNH'	JAZ Pro304 C=**O**-NH	1.9	1.9
COI1 Tyr379 OH	JAZ Gln305 dC=**O**-NH_2	3.8	3.8
JAZ Ala309 NH	COI1 Glu84 C=**O**	1.9	1.9

Data S1. Spectra of a new compound 1, related to Figures 2.

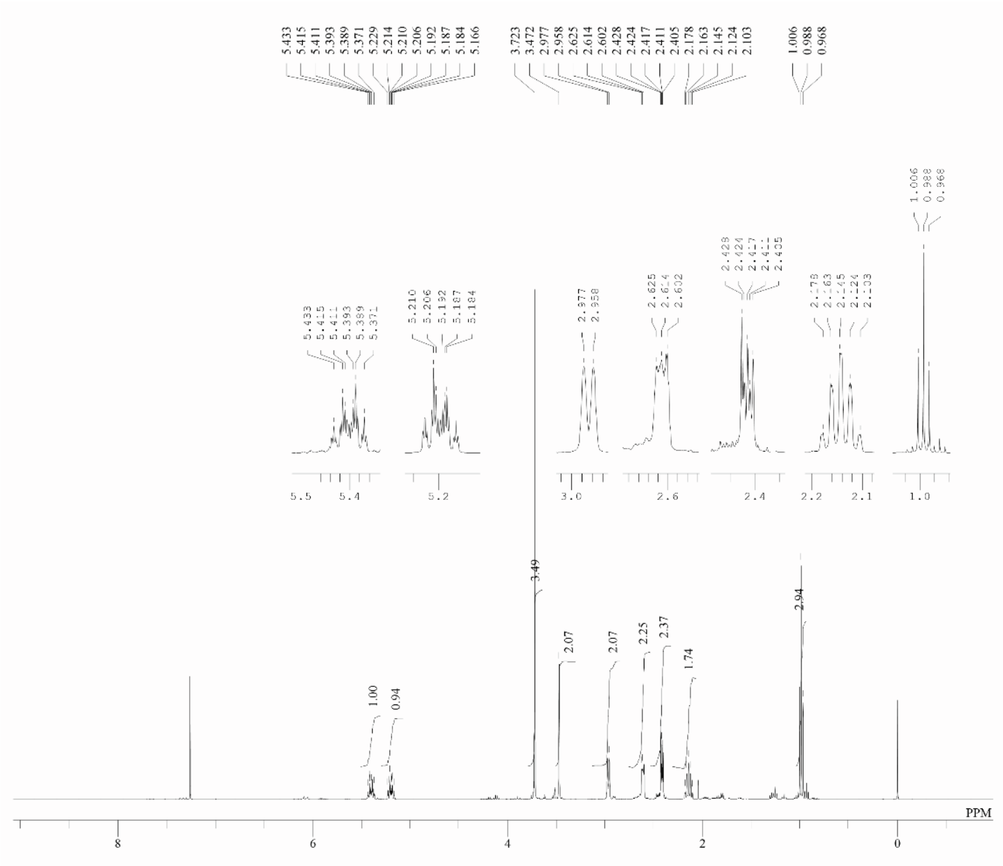

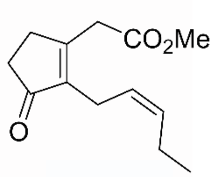

1

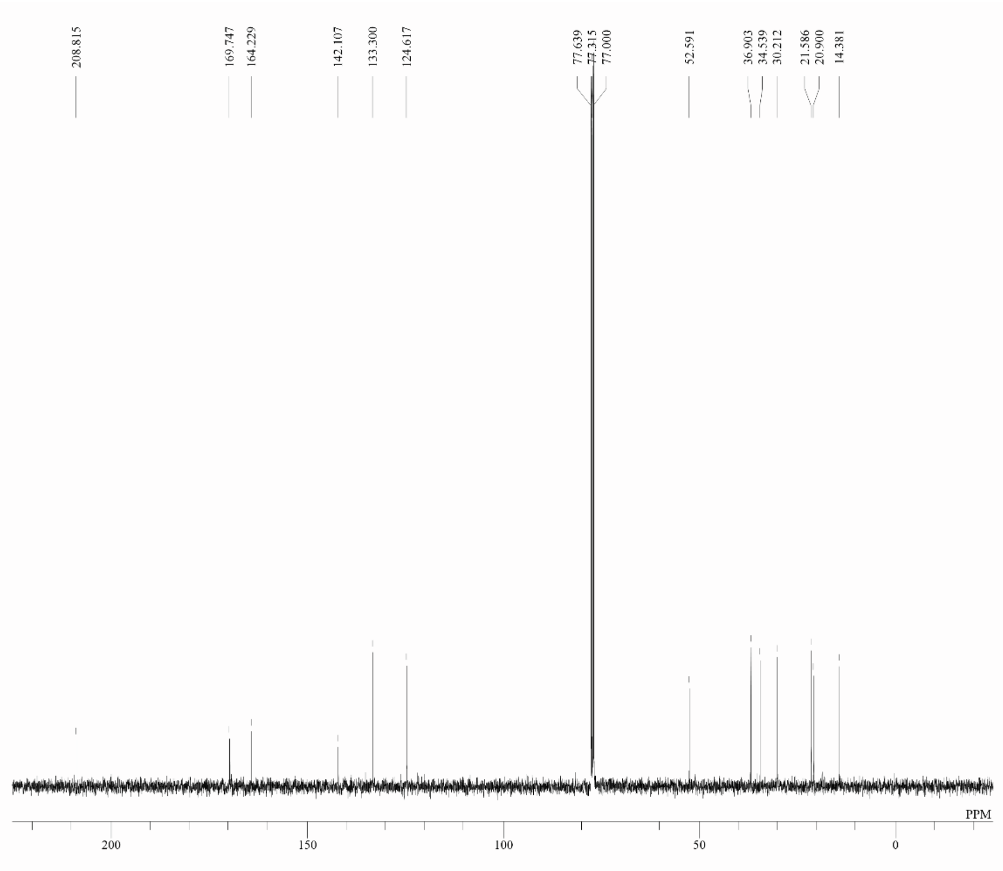

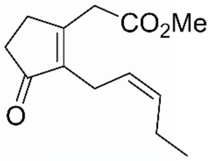

1

Data S2. Spectra of a new compound 2, related to Figures 2.

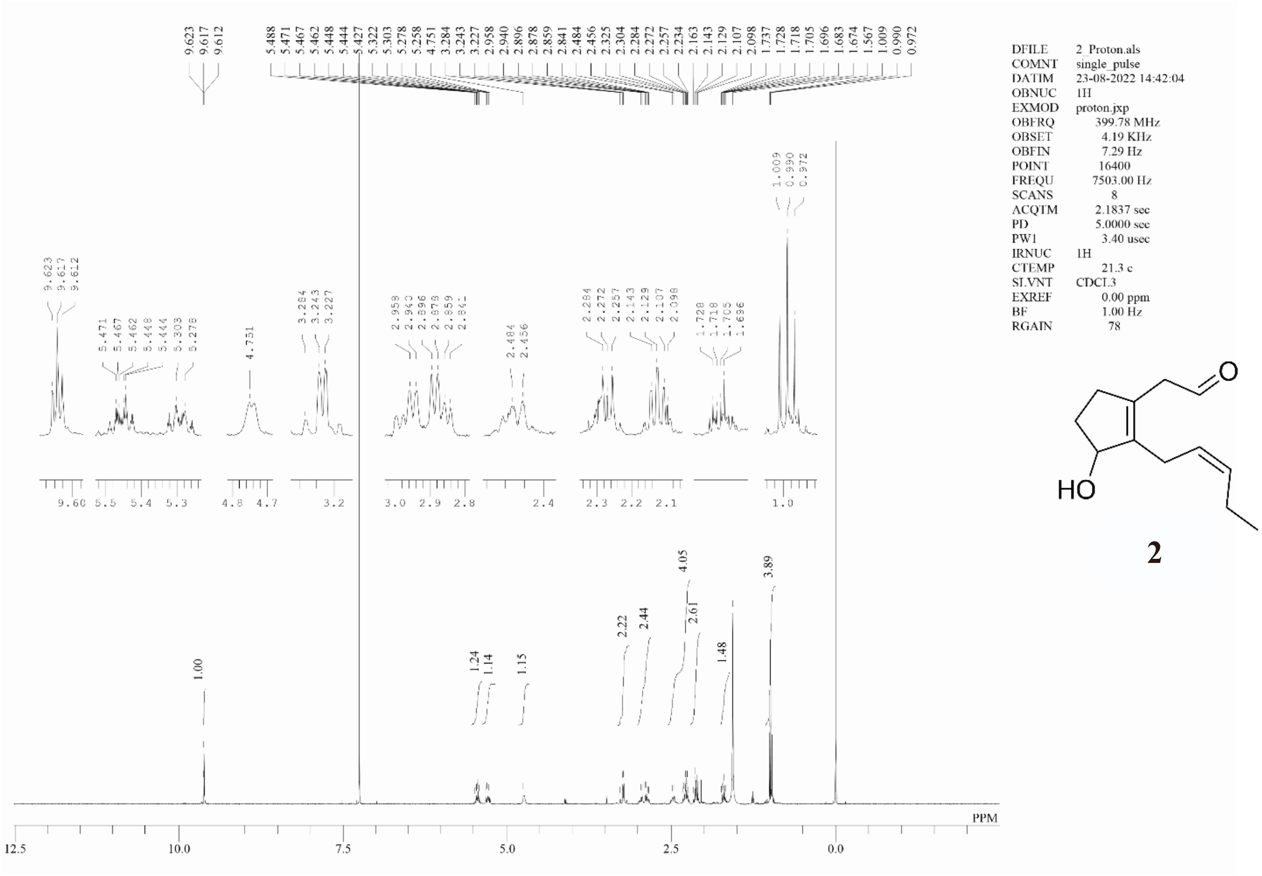

2

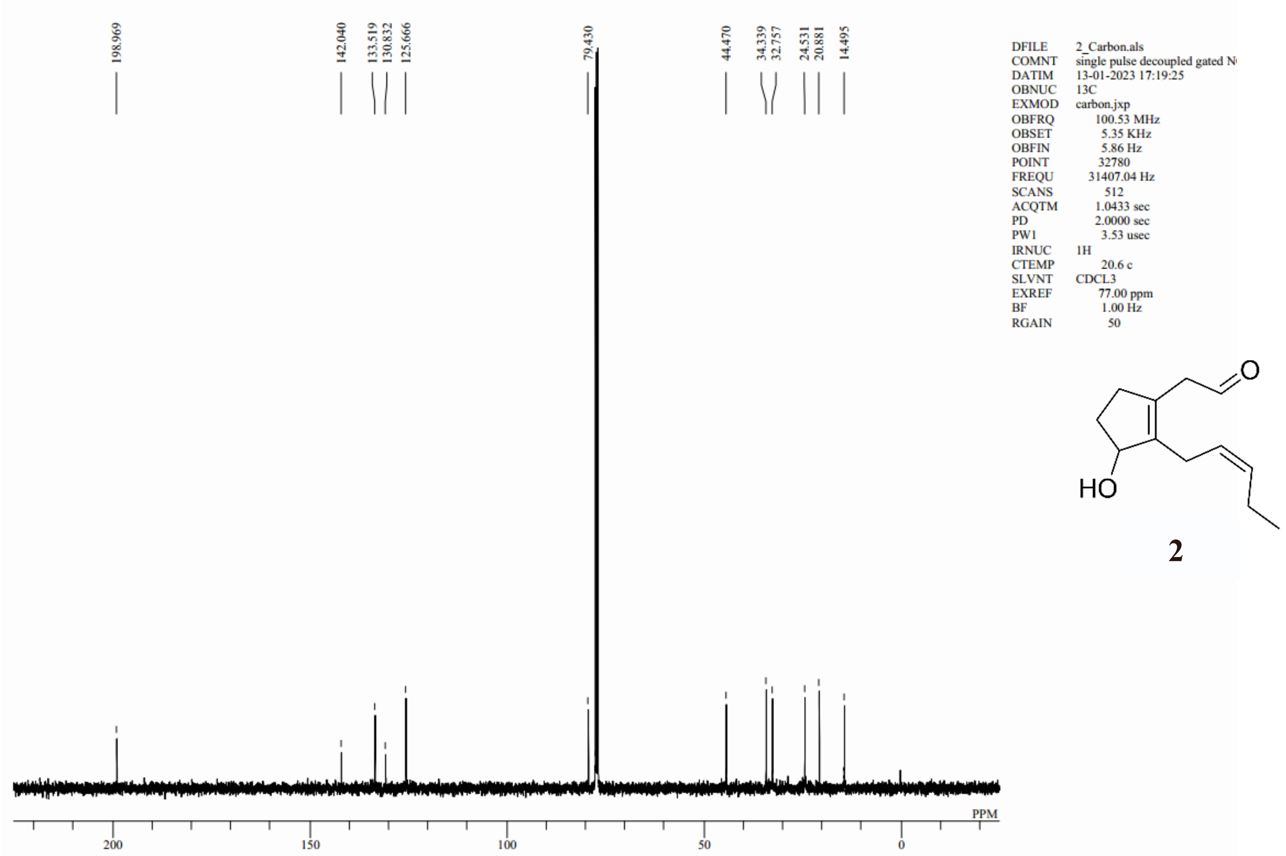

2

Data S3. Spectra of a new compound 3, related to Figures 2 and S2.

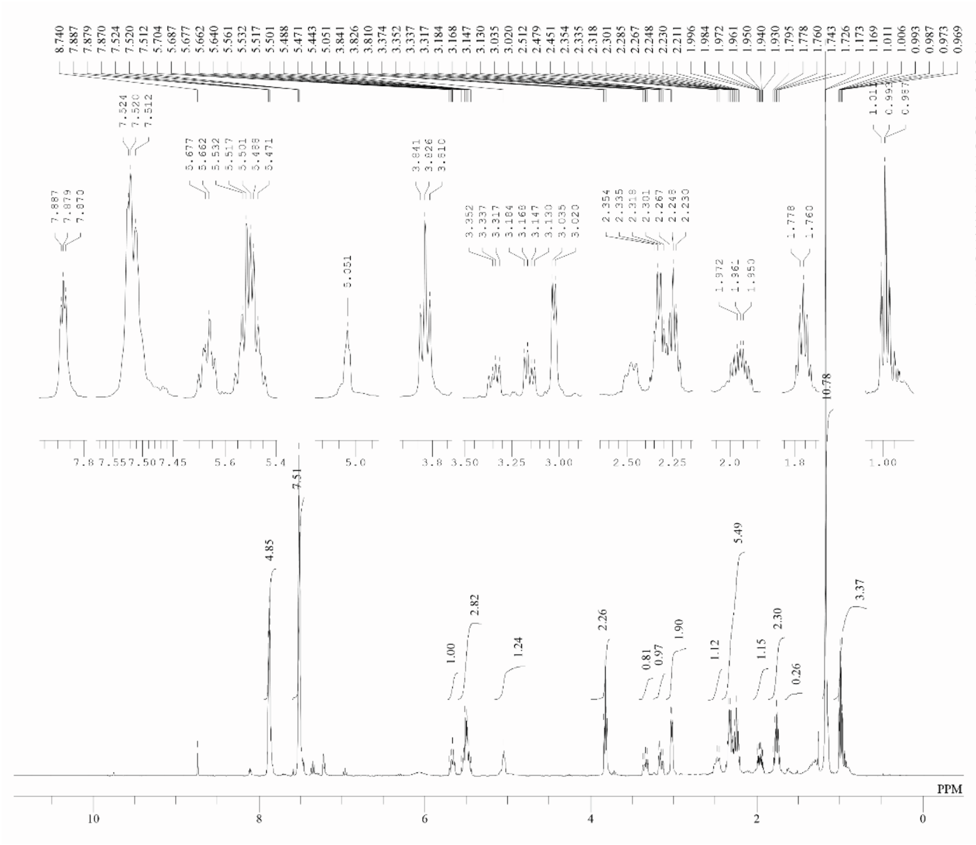

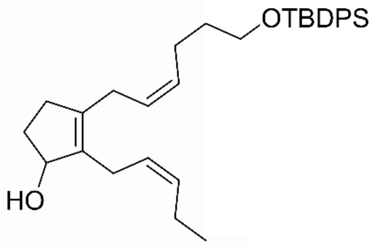

3

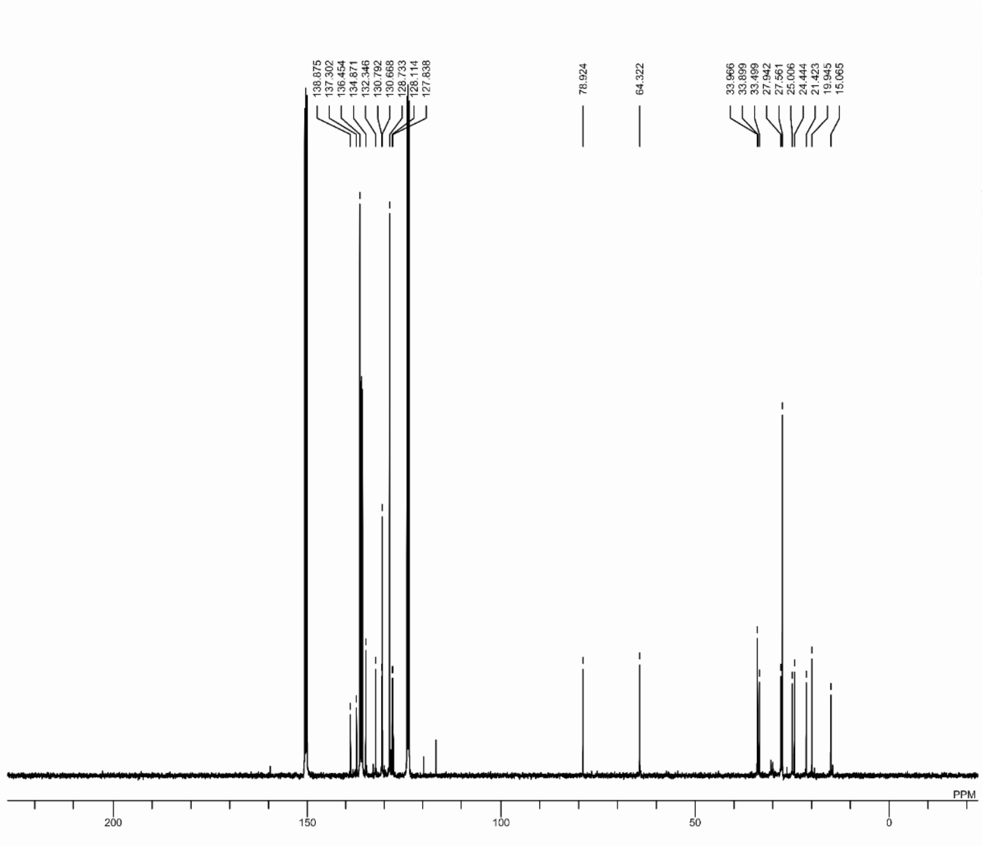

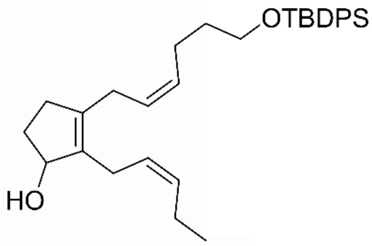

3

COSY

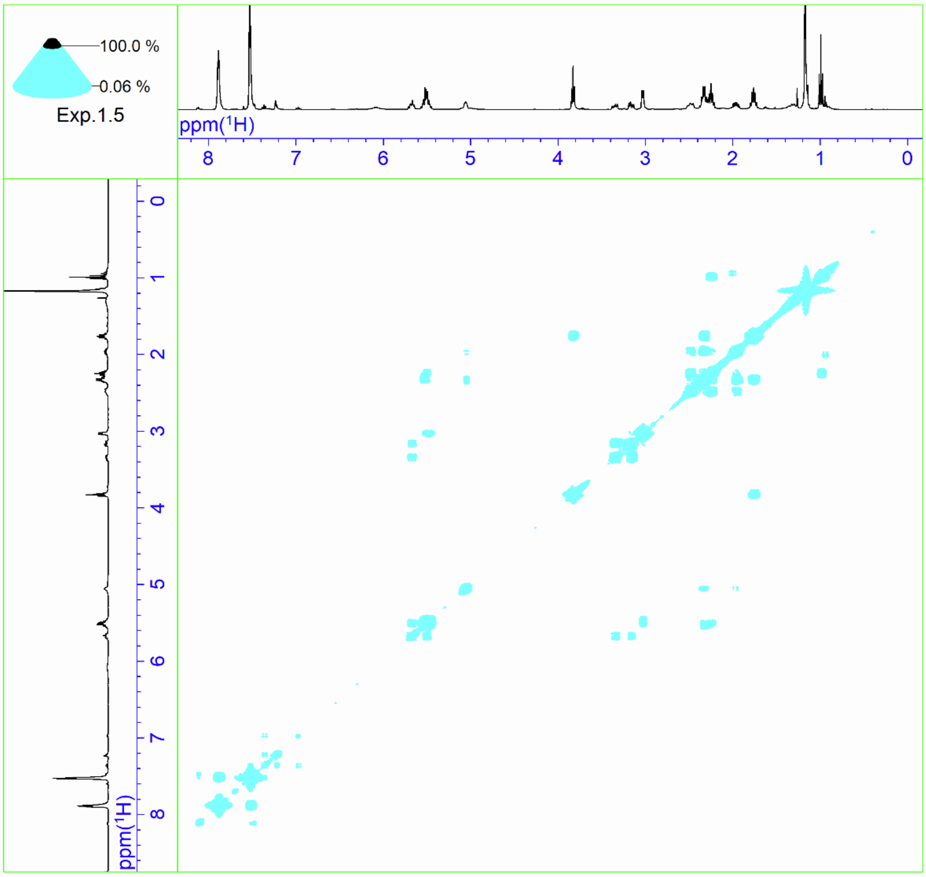
100.0 %
0.06 %
Exp.1.5
ppm(1H)
8
7
6
5
4
3
2
1
0

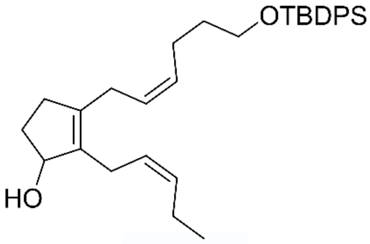
OTBDPS
HO

3

HSQC

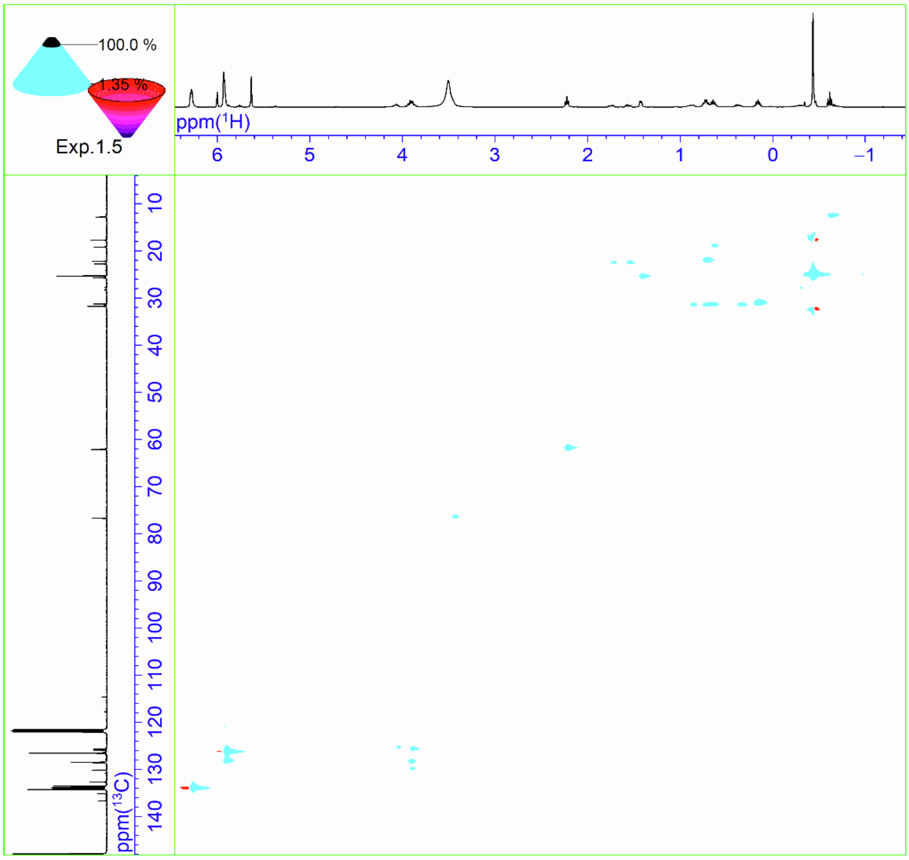
100.0 %
1.35 %
Exp.1.5
ppm(1H)
ppm(13C)

HMBC

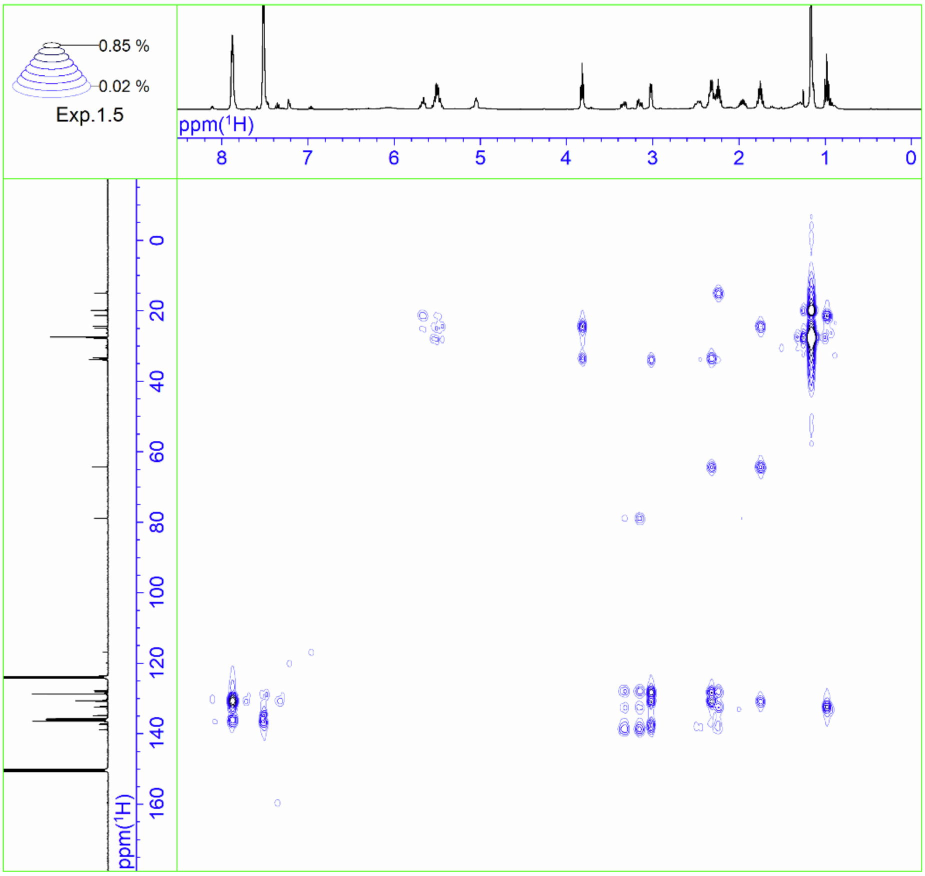

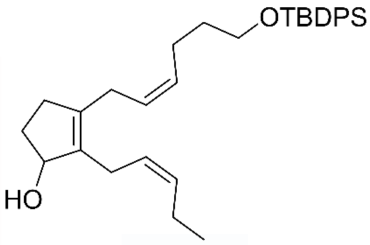

3

Dif. NOE

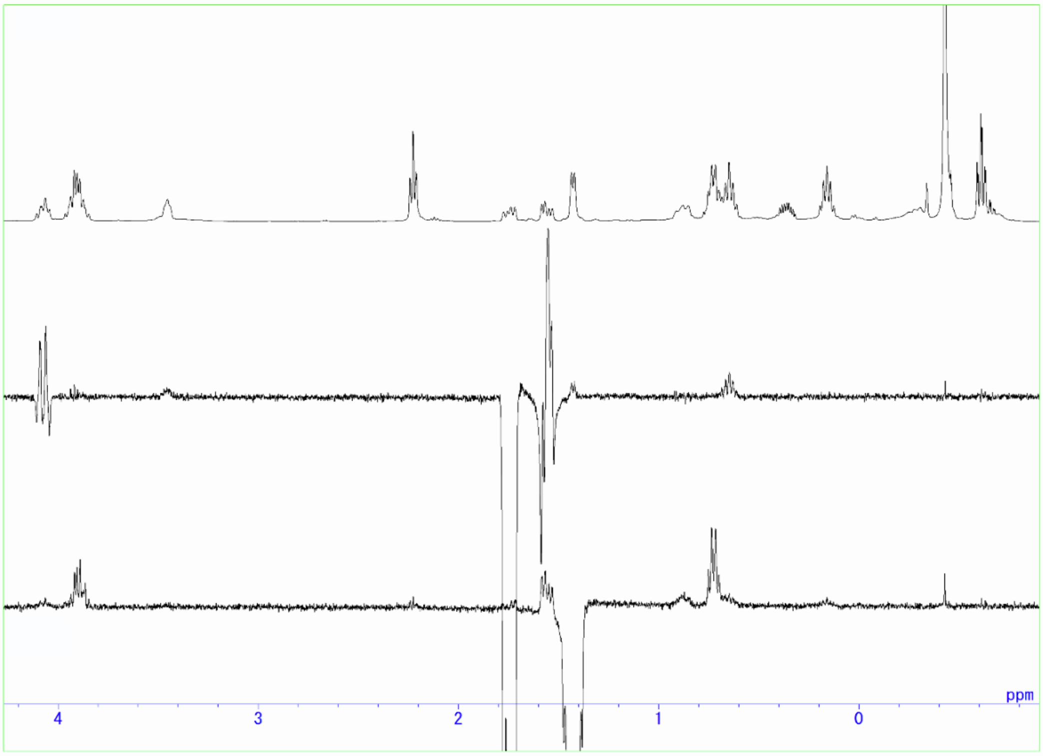

Data S4. Spectra of a new compound 4, related to Figures 2.

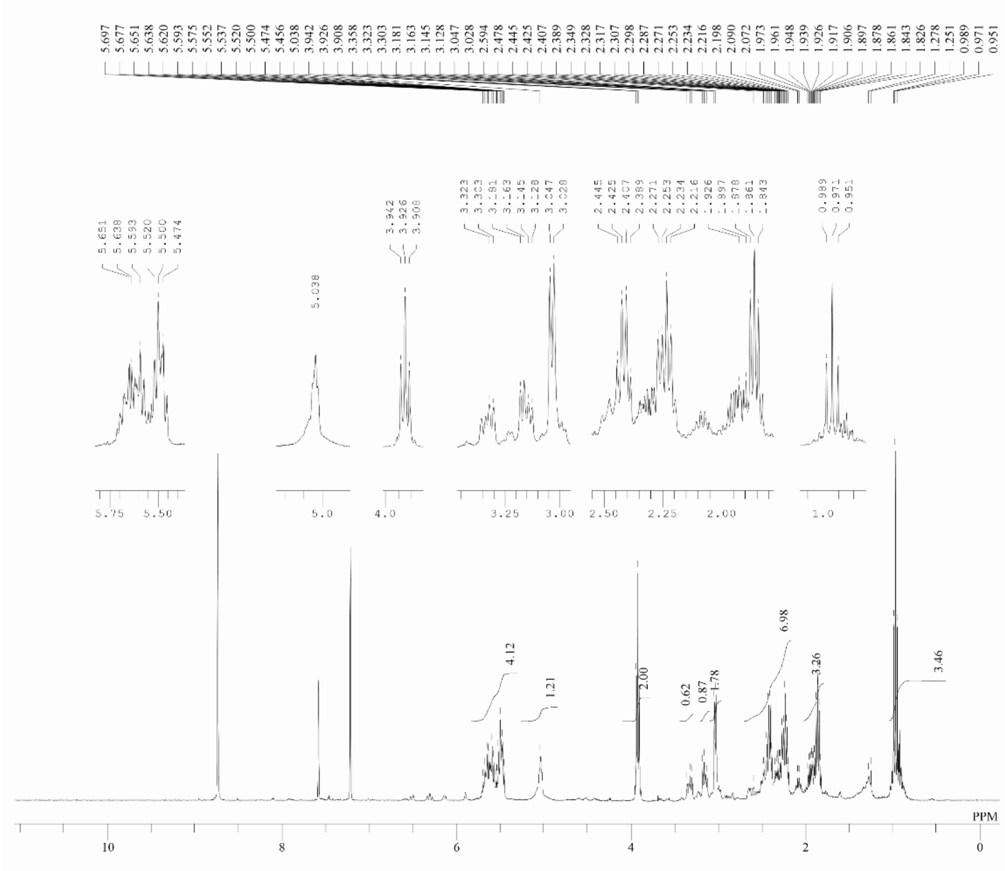

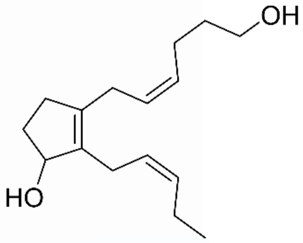

4

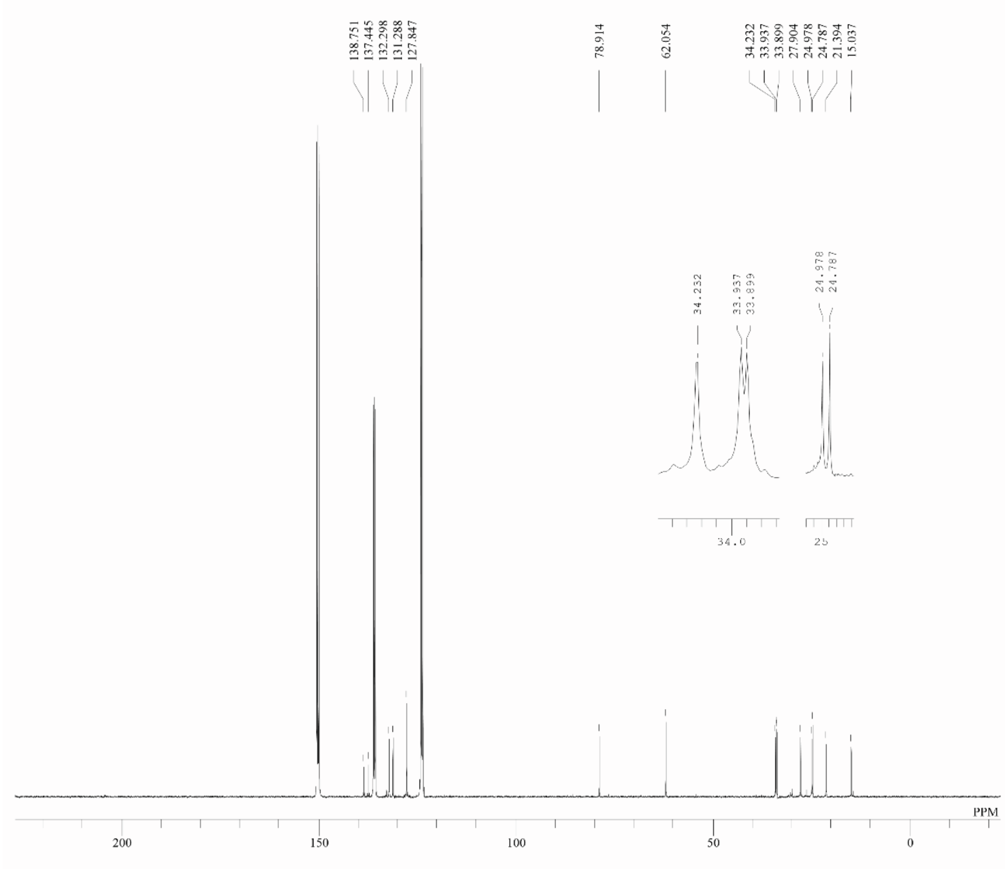

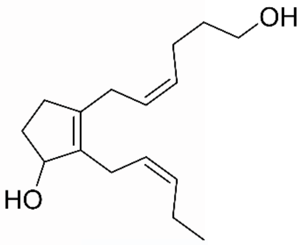

4

Data S5. Spectra of a new compound Δ^4-dinor-*iso*-OPDA, related to Figures 2.

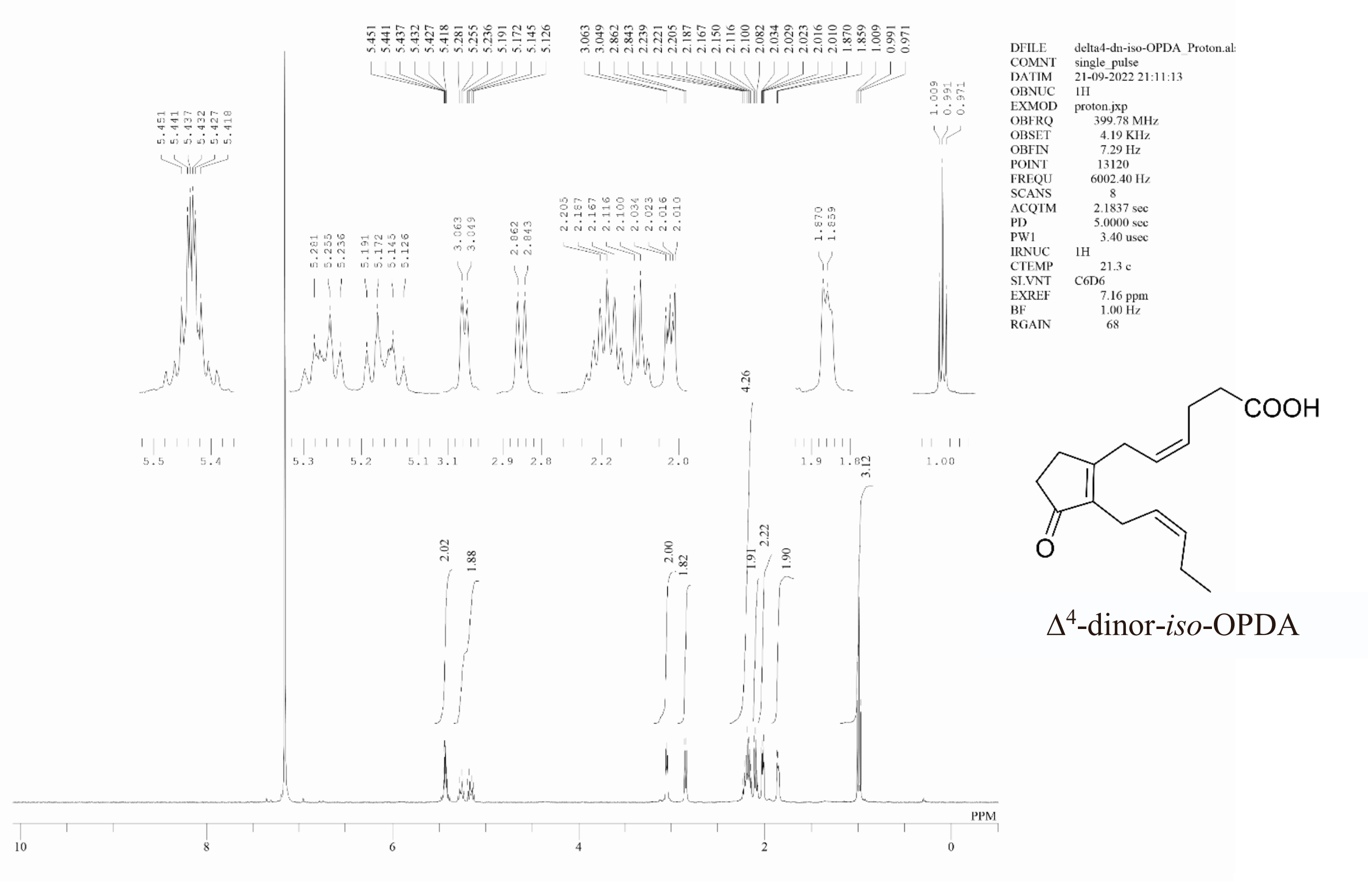

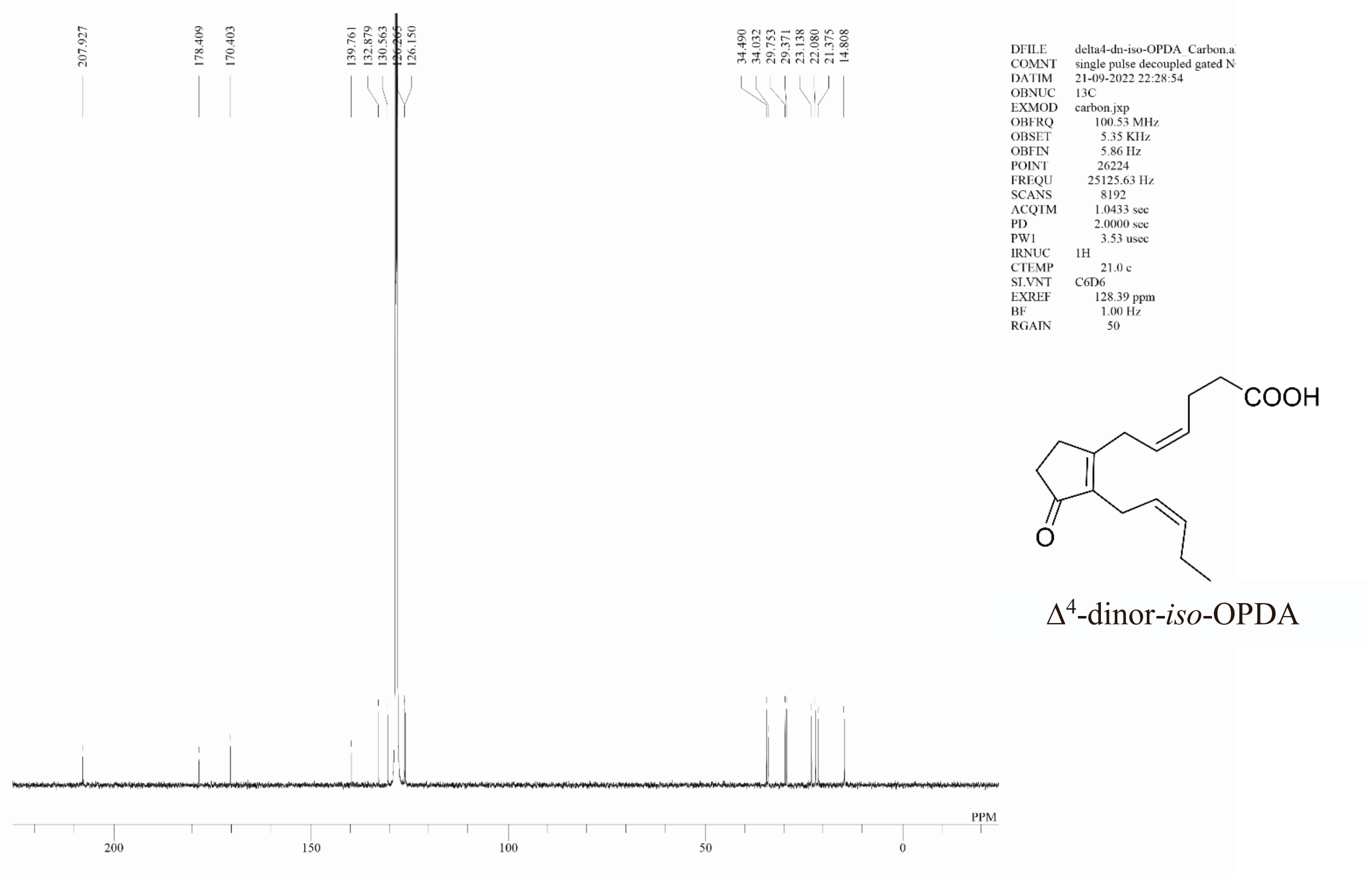

Data S6. Spectra of a new compound 6, related to Figures S8.

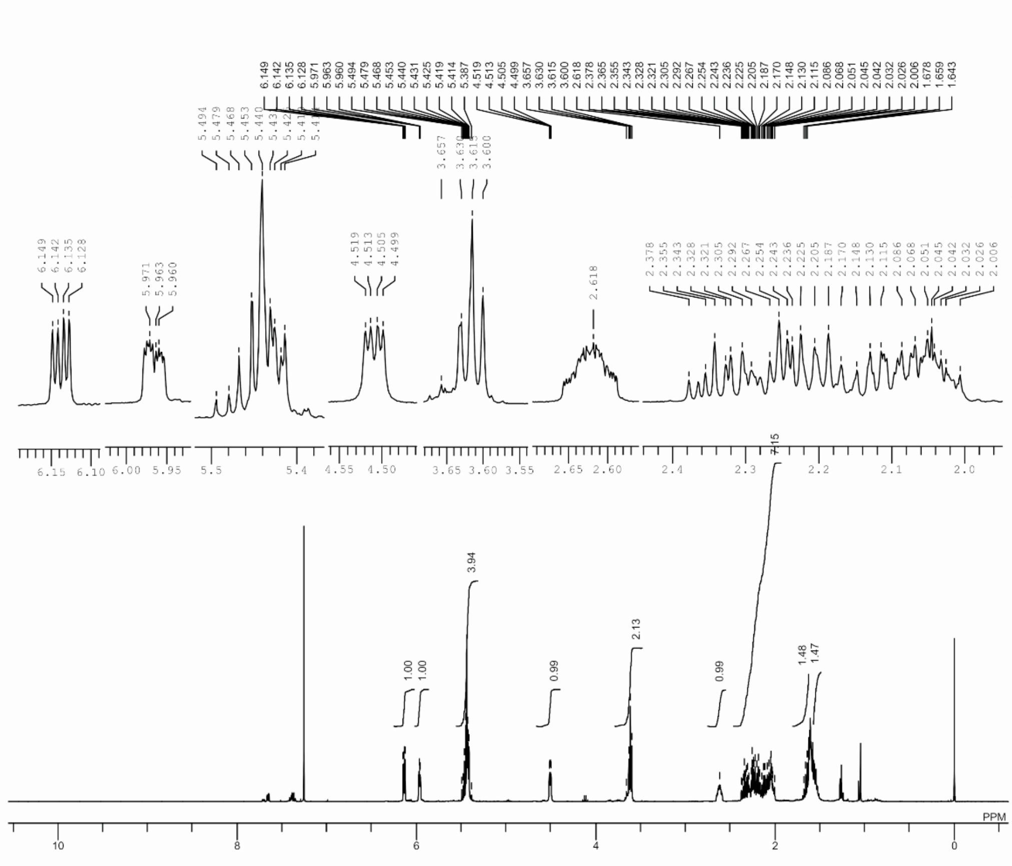

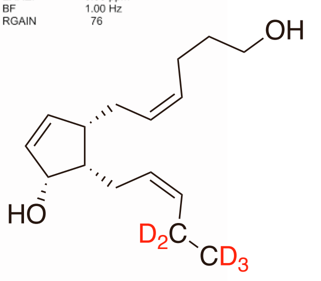

6

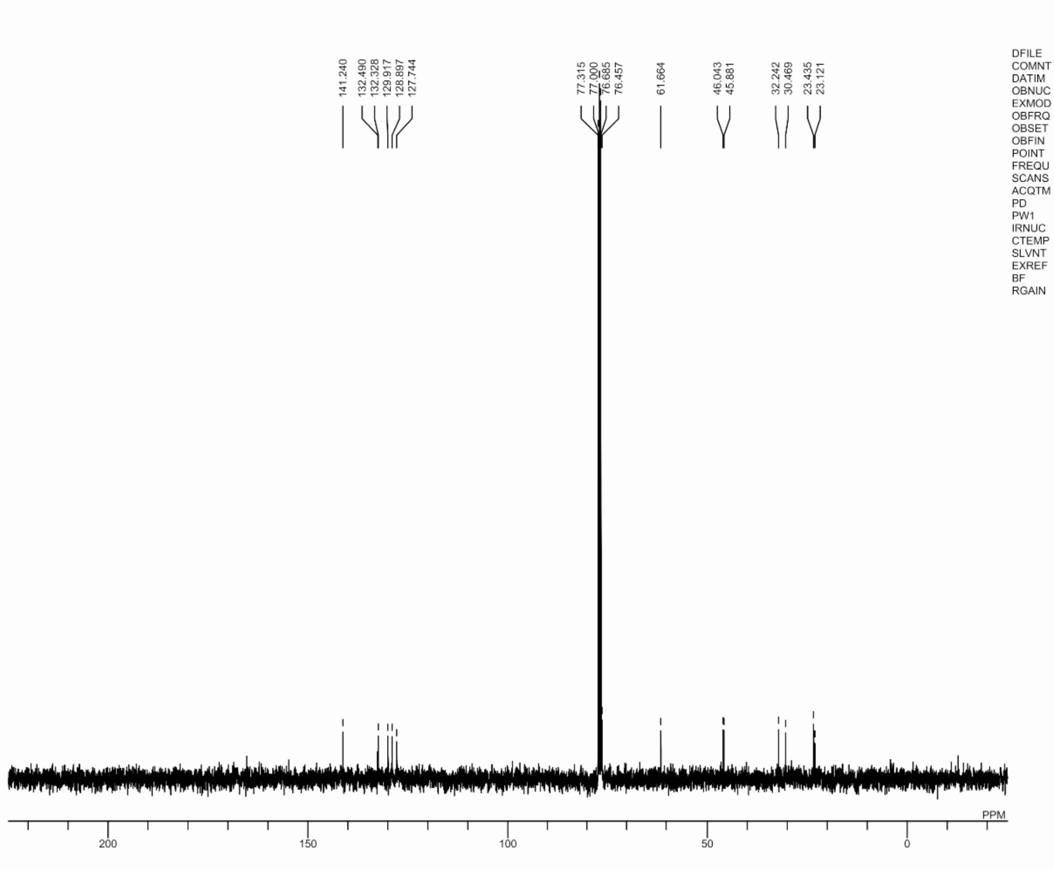

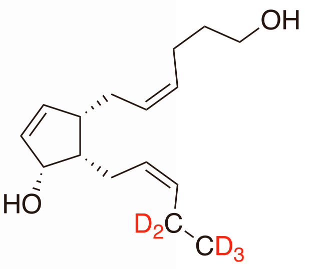

6

Data S7. Spectra of a new compound Δ[4]-dinor-*cis*-OPDA-d_5, related to Figures S8.

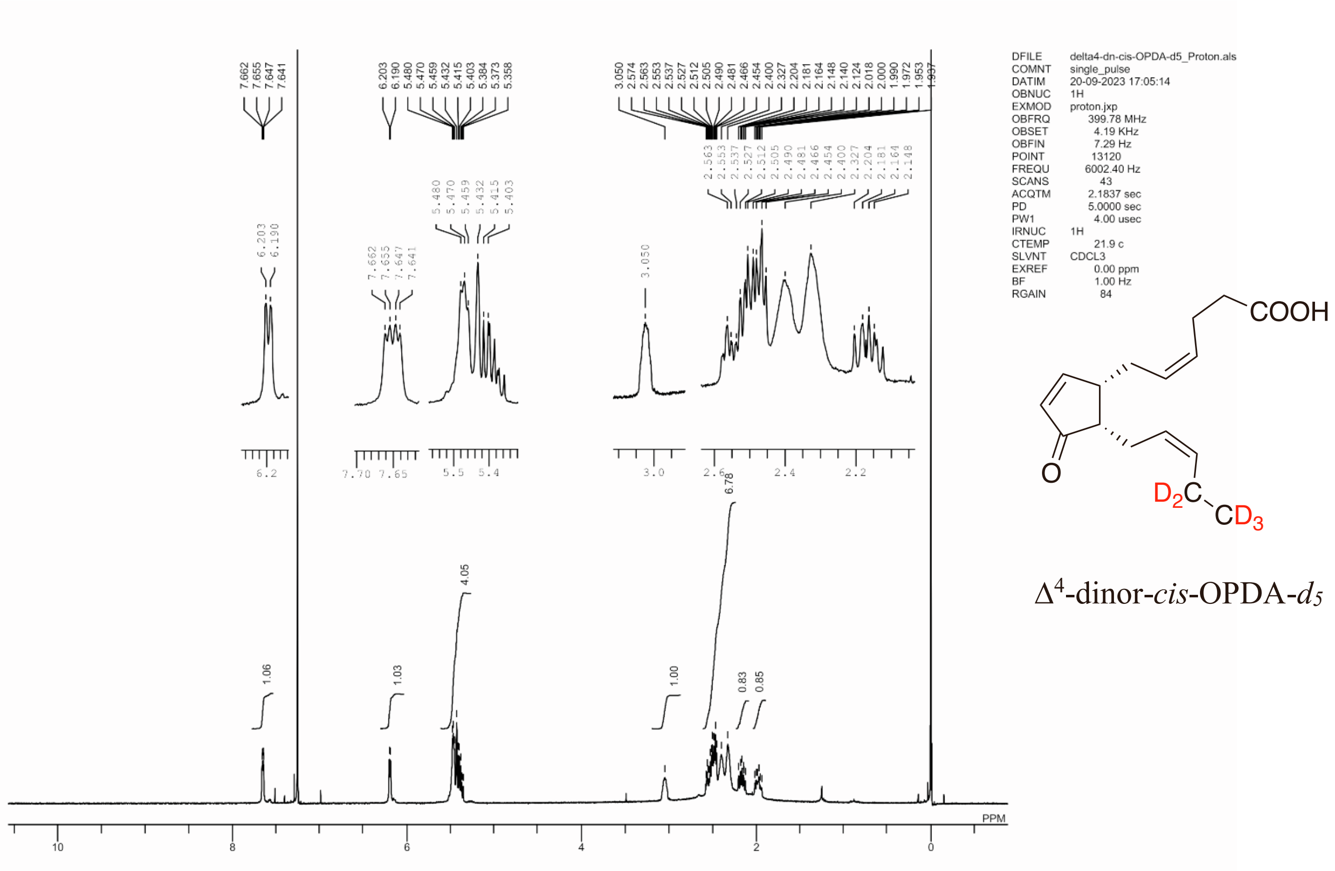

Δ[4]-dinor-*cis*-OPDA-d_5

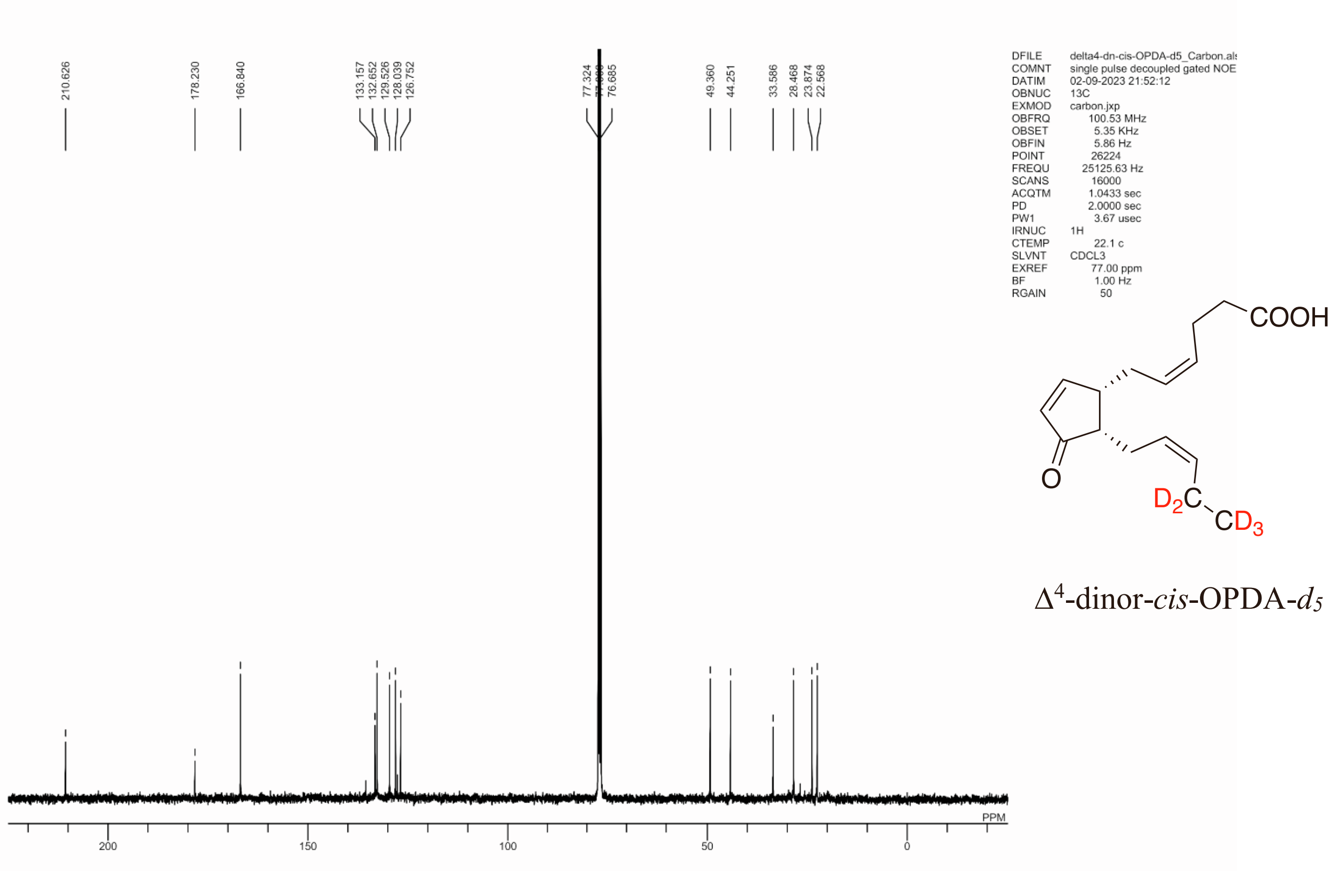

Δ[4]-dinor-*cis*-OPDA-d_5
